# Chemotherapeutic Potential of Chlorambucil-Platinum(IV) Prodrugs against Cisplatin-Resistant Colorectal Cancer Cells

**DOI:** 10.3390/ijms25158252

**Published:** 2024-07-28

**Authors:** Maria George Elias, Angelico D. Aputen, Shadma Fatima, Timothy J. Mann, Shawan Karan, Meena Mikhael, Paul de Souza, Christopher P. Gordon, Kieran F. Scott, Janice R. Aldrich-Wright

**Affiliations:** 1 School of Science, Western Sydney University, Sydney, NSW 2751, Australia; m.elias3@westernsydney.edu.au (M.G.E.); a.aputen@westernsydney.edu.au (A.D.A.); shawan.karan@westernsydney.edu.au (S.K.); c.gordon@westernsydney.edu.au (C.P.G.); 2 Medical Oncology, Ingham Institute for Applied Medical Research, Liverpool Hospital, Liverpool, NSW 2170, Australia; s.fatima@westernsydney.edu.au (S.F.); tim.mann@unsw.edu.au (T.J.M.); kieran.scott@westernsydney.edu.au (K.F.S.); 3 School of Medicine, Western Sydney University, Sydney, NSW 2751, Australia; 4 Mass Spectrometry Facility, Western Sydney University, Sydney, NSW 2751, Australia; m.mikhael@westernsydney.edu.au; 5 Nepean Clinical School, Faculty of Medicine and Health, University of Sydney, Kingswood, NSW 2747, Australia; paul.desouza@sydney.edu.au

**Keywords:** Pt^II^PHEN*SS*, Pt^II^5ME*SS*, Pt^II^56ME*SS*, chlorambucil, platinum(II), platinum(IV), cytotoxicity, cytoskeleton, apoptosis, spheroids

## Abstract

Chlorambucil-platinum(IV) prodrugs exhibit multi-mechanistic chemotherapeutic activity with promising anticancer potential. The platinum(II) precursors of the prodrugs have been previously found to induce changes in the microtubule cytoskeleton, specifically actin and tubulin of HT29 colon cells, while chlorambucil alkylates the DNA. These prodrugs demonstrate significant anticancer activity in 2D cell and 3D spheroid viability assays. A notable production of reactive oxygen species has been observed in HT29 cells 72 h post treatment with prodrugs of this type, while the mitochondrial membrane potential was substantially reduced. The cellular uptake of the chlorambucil-platinum(IV) prodrugs, assessed by ICP-MS, confirmed that active transport was the primary uptake mechanism, with platinum localisation identified primarily in the cytoskeletal fraction. Apoptosis and necrosis were observed at 72 h of treatment as demonstrated by Annexin V-FITC/PI assay using flow cytometry. Immunofluorescence measured via confocal microscopy showed significant changes in actin and tubulin intensity and in architecture. Western blot analysis of intrinsic and extrinsic pathway apoptotic markers, microtubule cytoskeleton markers, cell proliferation markers, as well as autophagy markers were studied post 72 h of treatment. The proteomic profile was also studied with a total of 1859 HT29 proteins quantified by mass spectroscopy, with several dysregulated proteins. Network analysis revealed dysregulation in transcription, MAPK markers, microtubule-associated proteins and mitochondrial transport dysfunction. This study confirms that chlorambucil-platinum(IV) prodrugs are candidates with promising anticancer potential that act as multi-mechanistic chemotherapeutics.

## 1. Introduction

Together, colon and rectal cancers (CRC) rank second in terms of mortality and are the third most prevalent cancer types diagnosed in the United States [1,2]. For early-stage colon cancer, surgery is the usual treatment, either combined with or without adjuvant chemotherapy (such as fluorouracil and cisplatin); nevertheless, drug resistance and reoccurrence occur [2,3,4,5,6,7,8,9]. Addressing the significant challenge of cancer resistance to standard therapies like cisplatin is pivotal for improving patient outcomes and prolonging survival.

Medicinal inorganic chemistry utilises metals to diagnose and treat diseases, where metals can become cations and interact with negatively charged biological molecules, facilitating charge manipulation and hydrolysis reactions [10,11,12,13]. This attribute has increased interest in utilising medicinal inorganic chemistry for synthesising anticancer agents [12,14]. The history of modern metal-based chemotherapy drugs dates back to 1844, when Michele Peyrone synthesised *cis*-diamminedichloroplatinum(II) (cisplatin), although its potential as an anticancer agent was only recognised in 1965 [15]. However, resistance and increased toxicity have made cisplatin’s broad use more difficult. To address these clinical impediments, second and third-generation platinum (Pt) drugs, mainly *cis*-diammine(1,1-cyclobutanedicarboxylato)platinum(II) (carboplatin) and *trans*-L-(1*R*,2*R*-diaminocyclohexane)oxalatoplatinum(II) (oxaliplatin), were developed [16]. In 1989, the Food and Drug Administration (FDA) approved carboplatin, used against head and neck, lung, and ovarian carcinoma cancers [17]. In 2002, the FDA approved oxaliplatin for advanced colorectal cancer [18]. In order to address concerns with toxicity and resistance, scientists are now designing the next generation of platinum-based medications [16,19,20,21,22].

**Scheme 1:**
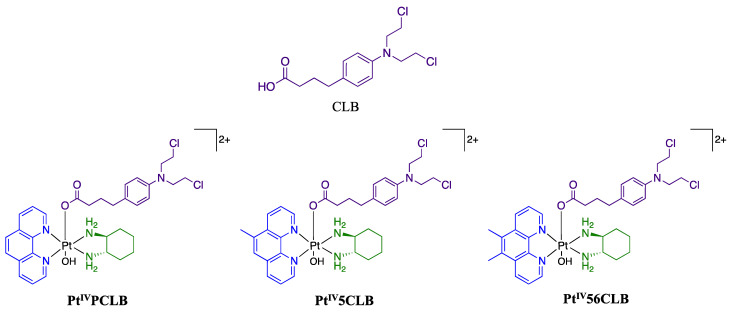
Structures of CLB and platinum(IV)-CLB prodrugs, **Pt^IV^PCLB**, **Pt^IV^5CLB**, and **Pt^IV^56CLB**. Nitrate (NO_3_)_2_ counterions were omitted for clarity.

In the pursuit of innovative platinum coordination complexes for oral administration, which is in part driven by concerns about low solubility and subsequently bioavailability of clinically administered platinum(II) complexes, platinum(IV) complexes have been explored [23,24,25,26]. Octahedral six-coordinate geometry with two axial coordination positions and elevated kinetic inertness, which can reduce off-target effects, make platinum(IV) complexes attractive potential prodrug scaffolds [20,27]. They undergo bioreductive activation in the presence of reductants such as ascorbic acid and glutathione but afford greater stability in the bloodstream allowing more drugs to reach their biological targets [20,24,28,29,30,31,32]. Advancements in understanding resistance mechanisms, tumour uptake, and structure–activity relationships can help advance the development of the next era of platinum chemotherapy complexes [16,33,34]. *Bis*-(acetate)-amine-dichloro-(cyclohexylamine)platinum(IV) (satraplatin), an octahedral platinum(IV) complex, was in clinical phase III stages for hormone-refractory prostate cancer treatment but failed to significantly increase survival benefits [28,35,36]. The literature reports a number of examples of multi-action platinum(IV) prodrugs that incorporate bioactive axial ligands that either target or inhibit polypeptides and enzymes [37,38]; however, limited development has been made towards conjugation of platinum with an axial ligand that functions in DNA alkylation, such as 4-[*bis*(2-chloroethyl)amino] phenylbutyric acid, also known as chlorambucil (CLB) and marketed as Leukeran (Scheme 1) [24,39,40]. The research into platinum prodrugs not only expands the therapeutic arsenal but also assists in shaping future treatment strategies, paving the way for new drug combinations and treatment regimens [16,41,42].

Our team has synthesised and characterised precursor platinum(II) and platinum(IV)-dihydroxy complexes with promising anticancer activity against various cancers [24,43]. Here, we explore the anticancer potential of platinum(IV) prodrugs utilising our non-DNA coordinating platinum(II) complexes as scaffolds; [Pt(1,10-phenanthroline)(1*S*,2*S*-diaminocyclohexane)](NO_3_)_2_ (**Pt^II^PHEN*SS***), [Pt(5-methyl-1,10-phenanthroline)(1*S*,2*S*-diaminocyclohexane)](NO_3_)_2_ (**Pt^II^5ME*SS***) and [Pt(5,6-dimethyl-1,10-phenanthroline)(1*S*,2*S*-diaminocyclohexane)](NO_3_)_2_ (**Pt^II^56ME*SS***), in conjugation with the DNA-alkylating agent, CLB (Scheme 1). The core platinum(II) complexes can be represented as [Pt^II^(H_L_)(A_L_)]^2+^ where H_L_ is the heterocyclic ligand (1,10-phenanthroline, 5-methyl-1,10-phenanthroline or 5,6-dimethyl-1,10-phenanthroline) and A_L_ is the ancillary ligand (1*S*,2*S*-diaminocyclohexane (*S*,*S*-DACH)). CLB is a member of the nitrogen mustard family, which is an older class of anticancer drugs that is effective by binding nucleotides like guanine and adenine, at the N(7) and N(3) positions in DNA which induces DNA crosslinks [44,45,46,47]. CLB has been used to treat leukaemia, Hodgkin’s lymphoma and ovarian cancer; however, its efficacy has been limited due to its low bioavailability and poor selectivity [48,49,50,51]. Using a platinum(IV) scaffold to deliver CLB is a possible approach to increase its bioavailability, as reported for cisplatin/oxaliplatin-CLB prodrugs. Two platinum(IV) derivatives of cisplatin-bearing CLB showed superior cytotoxicity, particularly against cisplatin-resistant cancer cell lines exhibiting potential to overcome chemoresistance [52]. The design also induced increased DNA damage by blocking double-strand break repair. These platinum(IV) derivatives exhibited longer retention times and minimal in vivo toxicities [52,53]. Another team has established that conjugating CLB to cisplatin improves cellular uptake and facilitates passive diffusion through cell membranes, aiding in reducing chemoresistance in cisplatin-resistant cancer cells [54,55]. These studies confirm that a cisplatin(IV) scaffold with two coordinated CLBs can overcome chemoresistance and improve cancer-killing properties. Our study investigates the biological mechanism of action of the CLB-platinum(IV) prodrugs, [Pt(1,10-phenanthroline)(*S*,*S*-DACH)(CLB)(OH)](NO_3_)_2_ (**Pt^IV^PCLB**), [Pt(5-methyl-1,10-phenanthroline)(*S*,*S*-DACH)(CLB)(OH)](NO_3_)_2_ (**Pt^IV^5CLB**), and [Pt(5,6-dimethyl-1,10-phenanthroline)(*S*,*S*-DACH)(CLB)(OH)](NO_3_)_2_ (**Pt^IV^56CLB**), as illustrated in Scheme 1. The proposed mechanism of action of the prodrugs is that they will biologically act like their platinum(II) precursors after bioreductive activation in effectively disrupting the microtubule cytoskeleton, inducing reactive oxygen species (ROS) and reducing mitochondrial function, dysregulating metabolic processes and inducing cell death [43,56,57]. This study aims to investigate the anticancer mechanistic activity against cisplatin-resistant colorectal (HT29) cancer by determining the mode and localisation of uptake, the cellular ROS production, the effect on the mitochondrial membrane potential (MtMP) and the type of cell death and cell cycle arrest by flow cytometry, immunofluorescence, and further understand the mechanism of cell death by western blot together with proteomic studies. The rationale behind the investigation of platinum anticancer prodrugs in cisplatin-resistant colorectal cancer is multifaceted. It fulfils a vital need in the field of oncology, capitalises on the current understanding of platinum drug mechanisms, and shows potential for new therapeutic approaches. By progressing our understanding of these prodrugs, we can significantly impact the management and outcomes of patients with cisplatin-resistant colorectal cancer, ultimately striving towards more effective cancer therapies. Future in vivo investigations are dependent upon the performance of the complexes in vitro.

## 2. Results

### 2.1. Cytotoxicity Evaluation of Chlorambucil-Platinum(IV) Prodrugs in Colorectal Cancer Cells, Normal Epthelial Cells and Spheroid Model of Colorectal Cancer

**Figure 1:**
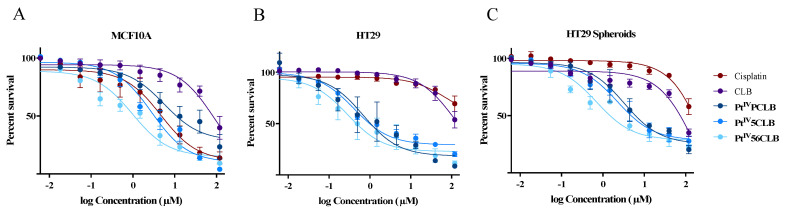
Effect of chlorambucil-platinum(IV) prodrugs on the survival of cancer, normal epithelial cells and spheroids. (**A**) MCF10A, (**B**) HT29 Cells, and (**C**) HT29 spheroids were treated with 3-fold dilutions of the different agents including cisplatin, CLB, **Pt^IV^PCLB**, **Pt^IV^5CLB**, or **Pt^IV^56CLB** starting with a concentration of 150 μM and assayed for cell viability as described in Section 4.3 and Section 4.4. The data for cisplatin was obtained from previously reported data run in parallel [43]. Data points denote mean ± SEM from three independent experiments where samples were run in triplicates.

The cytotoxic effect of the CLB-platinum(IV) prodrugs was examined in HT29 colon cancer cells and spheroids and MCF10A non-tumourigenic immortalised human breast epithelial cells 72 h post-treatment (Figure 1 and Table 1). All prodrugs exhibited exceptional potency compared to cisplatin and their ligand CLB in HT29 cells. The potency of the prodrugs relative to cisplatin was highest in **Pt^IV^56CLB** (277-fold, *p* < 0.0001) > **Pt^IV^5CLB** (190-fold, *p* < 0.0001) > **Pt^IV^PCLB** (114-fold, *p* < 0.0001). The ligand, CLB was less potent than cisplatin (*p* < 0.001). The MCF10A cell line is often used as a “normal” cell line in cytotoxicity studies [58,59]. Although MCF10A expresses markers that indicate it has features of both epithelial and luminal breast cells, it is derived from human mammary gland tissue, is non-tumorigenic (i.e., pre-malignant) and has an epithelial morphology in 2D culture, which makes it a suitable model for comparing the effects of compounds or treatments on non-tumorigenic cells versus cancerous cells [58,60]. Researchers commonly use MCF10A cells to calculate the selective cytotoxicity index or therapeutic index of drugs or experimental treatments, as these cells provide a baseline for comparison against cancer cell lines [58,61,62,63,64,65,66,67,68,69,70]. The selective cytotoxicity index (SCI) (Table 2) was calculated for all the complexes by dividing their half-maximal inhibitory concentration (IC_50_) (Table 1) in the “normal” epithelial cell line MCF10A by that of the cancer cell line. The examined prodrugs showed selectivity in HT29 (cisplatin-resistant) with an SCI > 3 across all treatments relative to the clinical platinum anticancer drug; cisplatin with an SCI of 0.06. Based on this, the selectivity was highest for **Pt^IV^PCLB** (SCI of 8.19) > **Pt^IV^5CLB** (SCI of 6.88) > **Pt^IV^56CLB** (SCI of 3.46). It was anticipated that cisplatin would have the least SCI as HT29 is a cisplatin-resistant cell line.

Spheroids from HT29 were bio-printed, grown and treated for 72 h (Figure 1 and Figure S2) to determine the cytotoxic effect of the prodrugs in a more complex system that closely mimics drug responses in vivo [71]. The cytotoxicity in 3D spheroids was reduced compared to 2D cells, but the order of potency of the prodrugs remained largely unchanged. All remained more potent than either cisplatin or the ligand CLB. The potency of the prodrugs relative to cisplatin on HT29 spheroids was highest in **Pt^IV^56CLB** (635-fold, *p* < 0.0001) > **Pt^IV^5CLB** (196-fold, *p* < 0.0001) > **Pt^IV^PCLB** (1135-fold, *p* < 0.0001). No significant differences were observed in IC_50_ among all complexes in both 2D and 3D growth conditions. Foreseeably, the IC_50_ for all complexes showed an increase in 3D culture relative to 2D culture with a non-significant average 4.2-fold increase in HT29 spheroids.

### 2.2. Cellular Uptake of the Chlorambucil-Platinum(IV) Prodrugs

**Figure 2:**
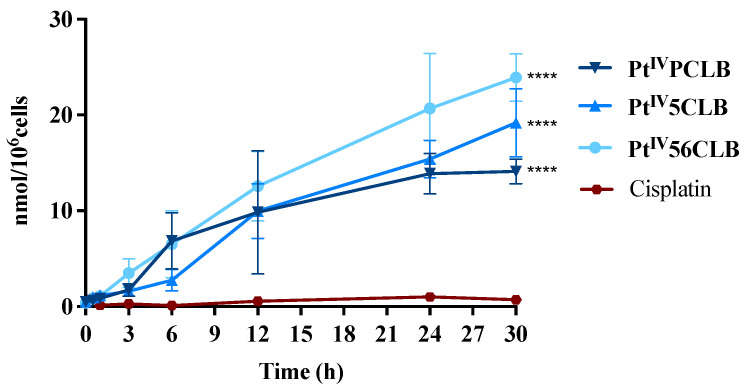
Cellular uptake of **Pt^IV^PCLB**, **Pt^IV^5CLB**, and **Pt^IV^56CLB**. ICP-MS analysis of the uptake of platinum in HT29 cells at 0, 0.5, 1, 3, 6, 12, 24, and 30 h as described in Section 4.5. The data for cisplatin were obtained from previously reported data run in parallel [43]. *n* = 3 from three separate experiments where samples were run in triplicates. Data points denote mean ± SEM and are expressed in nmol/10^6^ cells. **** *p* < 0.0001 in comparison to cisplatin, as measured by two-way ANOVA.

The cellular absorption of platinum in cells treated with **Pt^IV^PCLB**, **Pt^IV^5CLB**, and **Pt^IV^56CLB** relative to cisplatin, each at a concentration of 3 µM, was measured by inductively coupled plasma mass spectrometry (ICP-MS). Cisplatin showed the least cellular uptake compared to the prodrugs in HT29 cells. The cellular uptake of all complexes (Figure 2 and Figure S3) was time-dependent with **Pt^IV^PCLB** plateauing at 24–30 h, while **Pt^IV^5CLB** and **Pt^IV^56CLB** progressively increased up to 30 h in HT29-treated cells. Cisplatin cell uptake remained fairly constant and decreased intracellularly from 24 to 30 h. The mean cellular concentrations (nmol/10^6^ cells) were observed to be highest (0.39 ± 0.14 (0 h) to 23.93 ± 4.32 (30 h)) for **Pt^IV^56CLB** > **Pt^IV^5CLB** (0.51 ± 0.42 (0 h) to 19.18 ± 6.17 (30 h)) > cisplatin (0.36 ± 0.04 (0 h) to 0.71 ± 0.18 (30 h)) in HT29 cells (Table S3). The increased uptake observed in **Pt^IV^56CLB** may be attributed to increased hydrophobic interactions with biomolecules for cell uptake due to the introduction of the methyl groups in **Pt^IV^56CLB** relative to **Pt^IV^PCLB** [72]. Cellular volume is about ~1.7 pL [73], with equal distribution of the complexes inside the cell; the drug’s concentration has been calculated to be approximately 6655 μM, 9044 μM, and 11,280 μM per HT29 cell which is 2218, 3015, and 3760 times the starting concentration (3 μM) for **Pt^IV^PCLB**, **Pt^IV^5CLB**, and **Pt^IV^56CLB**, respectively. The ratio (intracellular/extracellular concentration) was estimated knowing that the extracellular concentration was 3 μM for each platinum complex (Figure S3 and Table S3). A significant accumulation of all prodrugs was observed over all time periods. At 30 h, **Pt^IV^56CLB** recorded the greatest intracellular/extracellular ratio in HT29 (3760.15 ± 1174.25), a result which is correspondingly 34-fold higher than the maximum ratio reported for cisplatin (with a ratio of 112.14 ± 83.99 at 30 h of treatment). The increased cellular concentrations were consistent with an active cell uptake transport mechanism. This phenomenon is observed in the cellular uptake of cisplatin, which is both by active import via membrane transport proteins, particularly the copper transporter (CTR1), and passive diffusion [74]. The relative uptake of the complexes compared to cisplatin and to each other was evaluated by a two-way ANOVA with the first variable being time and the second variable being response of each complex. In HT29 cells, the uptake of **Pt^IV^PCLB**, **Pt^IV^5CLB**, and **Pt^IV^56CLB** were all significant relative to cisplatin (*p* < 0.0001).

### 2.3. Mode of Uptake of Chlorambucil-Platinum(IV) Prodrugs

**Figure 3:**
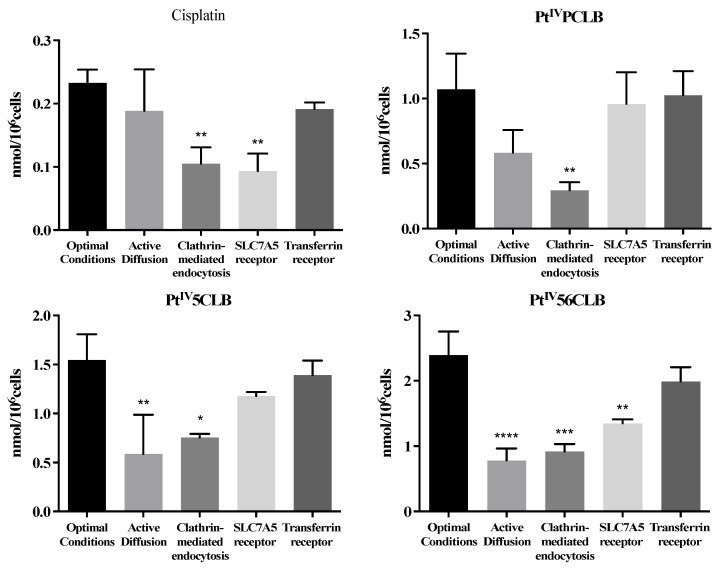
Mode of cellular uptake of platinum in HT29. After incubation at 37 °C or 4 °C, as well as after blockage of the SLC7A5, transferrin receptor, or clathrin-mediated endocytosis, the intracellular amount of platinum was determined by ICP-MS as described in Section 4.6. The data for cisplatin were obtained from previously reported data run in parallel [43]. Data denote mean ± SEM of three independent experiments where samples were run in triplicates. * *p* < 0.05, ** *p* < 0.01, *** *p* < 0.001 and **** *p* < 0.0001 in comparison to the optimal conditions, as measured by one-way ANOVA.

To ascertain whether any crucial transport proteins or uptake processes are involved in the absorption of the prodrugs intracellularly, the cell uptake of **Pt^IV^PCLB**, **Pt^IV^5CLB**, and **Pt^IV^56CLB** was measured by ICP-MS after blocking the mechanism of interest represented in Figure 3 (nmol/10^6^ cells) and Figure S4 (μM/cell). The cell uptake of cisplatin was reduced at 4 °C (active diffusion) compared to 37 °C (optimal condition). Inhibition of clathrin-mediated endocytosis or SLC7A5 (Solute Carrier Family 7 Member 5) resulted in significantly reduced uptake (*p* < 0.01) in HT29 cells. Transferrin receptor (TfR) was also slightly reduced on inhibition. HT29 cells treated with **Pt^IV^PCLB** exhibited a reduction in uptake at 4 °C and a significant reduction in clathrin-mediated endocytosis on inhibition (*p* < 0.01). **Pt^IV^5CLB**-treated cells exhibited a significant reduction in uptake at 4 °C (*p* < 0.01) and in clathrin-mediated endocytosis (*p* < 0.05) on inhibition with a slight decrease in SLC7A5 receptor and TfR. **Pt^IV^56CLB**-treated cells showed a significant reduction in uptake at 4 °C (*p* < 0.0001), in clathrin-mediated endocytosis (*p* < 0.001), in SLC7A5 receptor (*p* < 0.01), and a slight decrease in TfR on inhibition.

### 2.4. Cellular Localisation of Chlorambucil-Platinum(IV) Prodrugs

**Figure 4:**
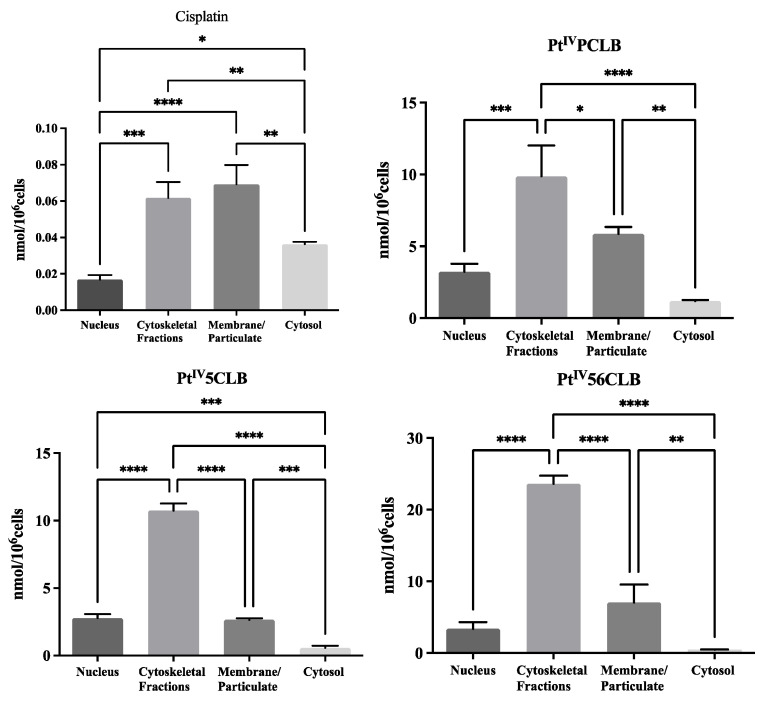
Cellular localisation of platinum in HT29 post 24 h. The intracellular amount of platinum was quantified by ICP-MS after cellular fractionation as described in Section 4.7. The data for cisplatin was obtained from previously reported data run in parallel [43]. Data denote mean ± SEM of three separate experiments with samples run in triplicates. * *p* < 0.05, ** *p* < 0.01, *** *p* < 0.001 and **** *p* < 0.0001 in comparison to each fraction, as measured by one-way ANOVA.

The cellular accumulation of the prodrugs was studied in subcellular fractions of HT29 cells and measured by ICP-MS (Figure 4 and Figure S5). The bar graphs represent the amount of platinum in the nuclear, cytoskeletal, membrane/particulate, and cytosolic fractions. The platinum was largely accumulated in the cytoskeletal fraction, while that of cisplatin was mostly localised to the membrane/particulate fraction (*p* < 0.0001 compared to nucleus) and cytoskeletal fraction (*p* < 0.001 compared to nucleus) in HT29 cells. It has previously been demonstrated that cisplatin is found in the cytosol and particulates in A2780 and ADDP ovarian cancer cell lines [75]. Although it has also been established that comparable platinum-based complexes primarily accumulate within the cytoskeleton, it cannot be assumed that the mechanisms underlying the prodrugs would be the same; therefore, our complexes were investigated [43,57]. The **Pt^IV^PCLB** was observed significantly localised to the cytoskeleton in comparison to the nucleus (*p* < 0.001), the membrane/particulate (*p* < 0.05), and cytosol fraction (*p* < 0.0001). **Pt^IV^5CLB** was also seen most significantly localised to the cytoskeleton in comparison to the nucleus (*p* < 0.0001), the membrane/particulate (*p* < 0.0001) and cytosol fraction (*p* < 0.0001). **Pt^IV^56CLB** was additionally most significantly localised to the cytoskeleton in comparison to the nucleus (*p* < 0.0001), the membrane/particulate (*p* < 0.0001), and cytosol fraction (*p* < 0.01) in HT29 cells. This observation was similarly observed by the prodrugs platinum(II) precursors and platinum(IV) derivatives, suggesting a similar mechanism of action [43].

### 2.5. Cell Death Analysis

**Figure 5:**
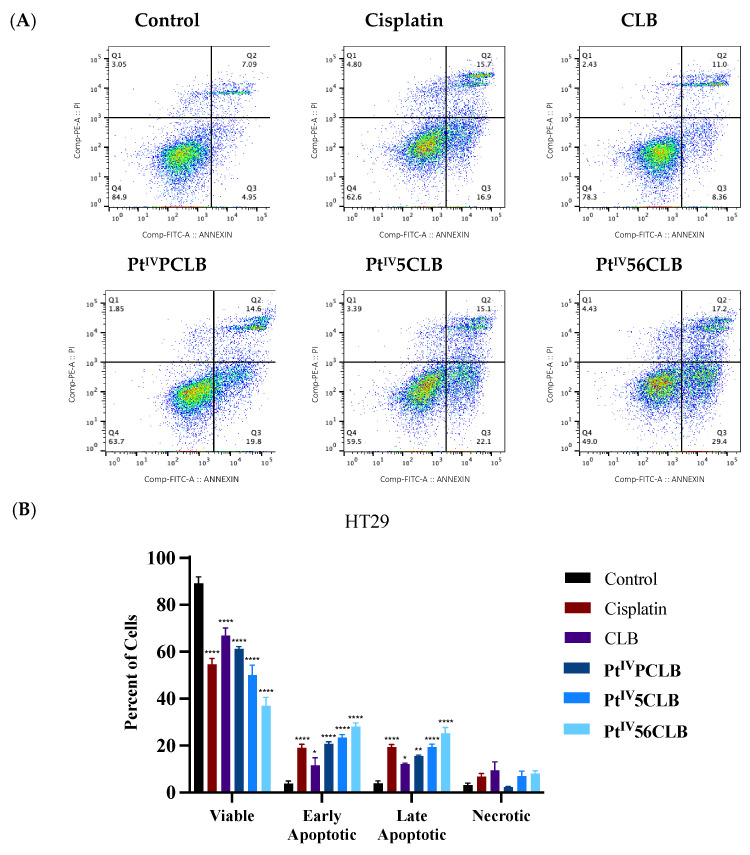
Flow cytometric analysis of cell death mediated by cisplatin, CLB, **Pt^IV^PCLB**, **Pt^IV^5CLB**, and **Pt^IV^56CLB**. HT29 cells were treated with the complex and analysed at 72 h as described in Section 4.8. (**A**). Representative dot plots and (**B**). Bar graphs demonstrating percent viable, early apoptotic, late apoptotic, and necrotic cells. Each treatment condition has been assigned a specific colour, as indicated to the right of the bar graph. The data for cisplatin were obtained from previously reported data run in parallel [43]. Data points represent mean ± SEM. *n* = 3 from three separate experiments with samples run in triplicates. * *p* < 0.05, ** *p* < 0.01 and **** *p* < 0.0001 compared to control group, as measured by one-way ANOVA.

Annexin V/PI staining was used to study the form of cell death after HT29 cells were treated with **Pt^IV^PCLB**, **Pt^IV^5CLB**, and **Pt^IV^56CLB** prodrugs, CLB, or cisplatin (Figure 5A,B). Flow cytometry was performed at 72 h post treatment. Cells treated with **Pt^IV^PCLB**, **Pt^IV^5CLB**, and **Pt^IV^56CLB** significantly underwent early apoptosis and late apoptosis. HT29-treated cells with cisplatin, **Pt^IV^PCLB**, **Pt^IV^5CLB**, and **Pt^IV^56CLB** induced significant early apoptosis (*p* < 0.0001), in addition to CLB (*p* < 0.05) (Figure 5B). Additionally, a significant increase in late apoptosis was observed by cisplatin (*p* < 0.0001), CLB (*p* < 0.05), **Pt^IV^PCLB** (*p* < 0.01), **Pt^IV^5CLB** (*p* < 0.0001), and **Pt^IV^56CLB** (*p* < 0.0001). An increase in necrotic cells was observed by cisplatin, **Pt^IV^5CLBB,** and **Pt^IV^56CLB** with most notable increase observed by CLB (Figure 5B).

### 2.6. Cell Cycle Arrest

**Figure 6:**
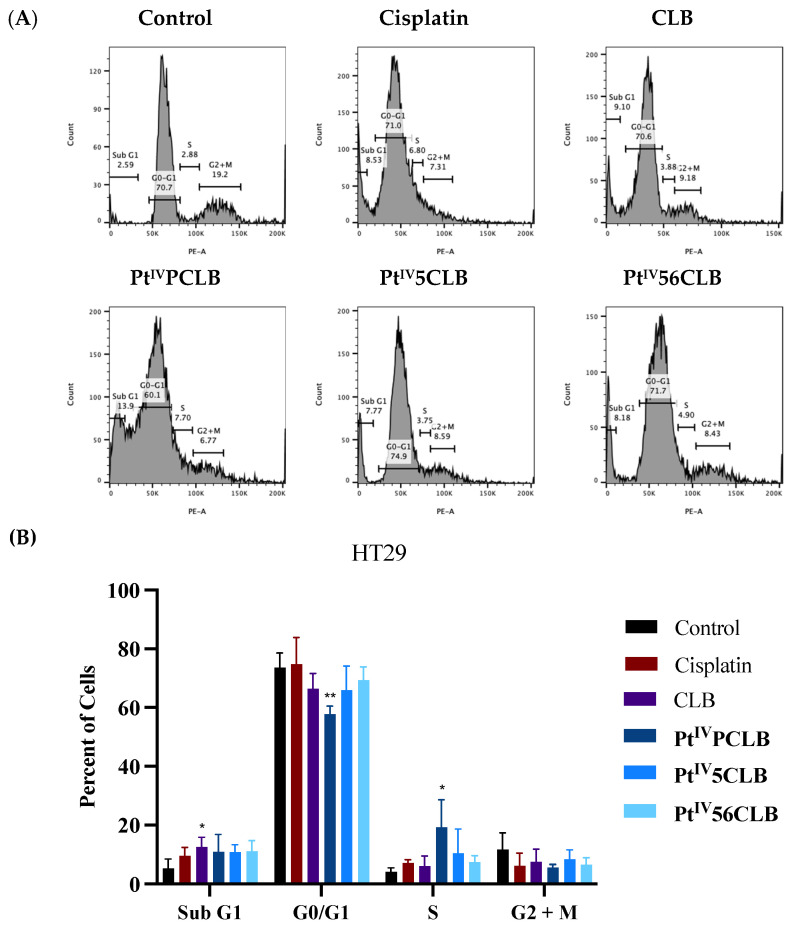
Flow cytometric analysis of cell cycle mediated by cisplatin, CLB, **Pt^IV^PCLB**, **Pt^IV^5CLB**, and **Pt^IV^56CLB**. HT29 cells were treated with IC_30_ concentration (cisplatin (IC_30_ 73.95 ± 1.97 μM), CLB (IC_30_ 61.94 ± 1.87 μM), **Pt^IV^PCLB** (IC_30_ 0.42 ± 1.61 μM), **Pt^IV^5CLB** (IC_30_ 0.30 ± 1.22 μM), **Pt^IV^56CLB** (IC_30_ 0.15 ± 1.62 μM) of each investigated agent and analysed at 72 h as described in Section 4.9. (**A**) Representative histogram plots and (**B**) Bar graphs show percent Sub G1, G0/G1, S and G2+M phases of cell cycle. The data for cisplatin were obtained from previously reported data run in parallel [43]. Data points represent mean ± SEM. *n* = 3 from three separate experiments where samples were run in triplicates. * *p* < 0.05 and ** *p* < 0.01 compared to control group, as measured by one-way ANOVA.

The cell-cycle profiles of HT29 cells treated for 72 h with **Pt^IV^PCLB**, **Pt^IV^5CLB**, **Pt^IV^56CLB**, CLB, or cisplatin, with concentrations equivalent to their IC_30_ values (cisplatin (IC_30_ 73.95 ± 1.97 μM), CLB (IC_30_ 61.94 ± 1.87 μM), **Pt^IV^PCLB** (IC_30_ 0.42 ± 1.61 μM), **Pt^IV^5CLB** (IC_30_ 0.30 ± 1.22 μM), **Pt^IV^56CLB** (IC_30_ 0.15 ± 1.62 μM)) at 72 h are shown in Figure 6. In HT29 treated cells, an increase in S phase of the cell cycle was observed across all complexes with an exceptional increase in **Pt^IV^PCLB** (*p* < 0.05) (Figure 6B). A decrease in G0/G1 (with significance *p* < 0.01 in **Pt^IV^PCLB**) and G2+M phase and increase in S phase of the cell cycle was detected across the prodrugs. A similar observation is seen by their platinum(II) precursors and platinum(IV) derivatives in HT29 cells [43].

### 2.7. Reactive Oxygen Species (ROS)

**Figure 7:**
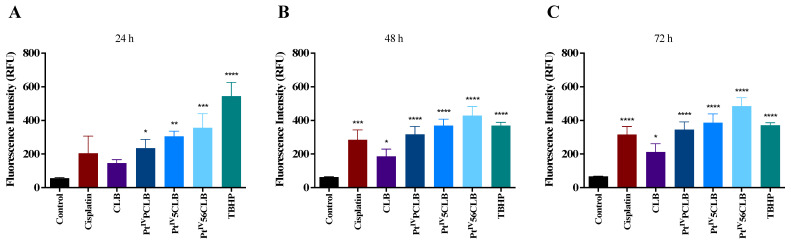
ROS production upon treatment with **Pt^IV^PCLB**, **Pt^IV^5CLB**, **Pt^IV^56CLB** prodrugs, CLB, cisplatin, and TBHP: t-butyl hydroperoxide were measured at (**A**) 24, (**B**) 48, and (**C**) 72 h in HT29 as described in Section 4.10. The data for cisplatin were obtained from previously reported data run in parallel [43]. Data points denote mean ± SEM. *n* = 3 from three separate experiments where samples were run in triplicate. * *p* < 0.05, ** *p* < 0.01, *** *p* < 0.001 and **** *p* < 0.0001 compared to control, as measured by one-way ANOVA.

HT29 cells were treated with **Pt^IV^PCLB**, **Pt^IV^5CLB**, and **Pt^IV^ 56CLB**, CLB, or cisplatin using individual IC_50_ concentration (Table 1) [24]. Upon the production of ROS, the 2’-7’-dichlorodihydrofluorescein diacetate (DCFDA) produced a fluorescent product. The measured fluorescence was accordingly proportional to the produced ROS (Figure 7 and Figure S6, and Table S4). HT29 cells treated with prodrugs **Pt^IV^PCLB**, **Pt^IV^5CLB** and **Pt^IV^56CLB** exhibited a significant increase in ROS starting at 24 h, through to 48 and 72 h (*p* < 0.0001). CLB exhibited increased ROS at 48 (*p* < 0.05) and 72 h (*p* < 0.05) and cisplatin showed increased ROS at 48 (*p* < 0.001) and 72 h (*p* < 0.0001). Tert-butyl hydroperoxide (TBHP: positive control) produced most ROS at 24 h (*p* < 0.0001) and while decreased, it remained significant at 48 and 72 h with *p* < 0.0001. A significant difference was observed in ROS produced by **Pt^IV^PCLB** relative to **Pt^IV^56CLB** (*p* < 0.05) at 72 h.

### 2.8. Mitochondrial Membrane Potential

**Figure 8:**
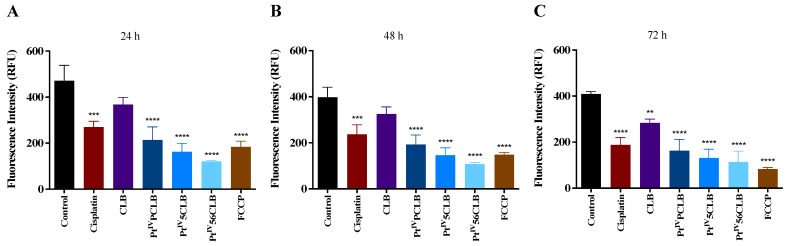
MtMP changes upon treatment with **Pt^IV^PCLB**, **Pt^IV^5CLB**, and **Pt^IV^56CLB** prodrugs, CLB, cisplatin, and FCCP were measured in HT29 cells at (**A**) 24, (**B**) 48 and (**C**) 72 h as described in Section 4.11. The data for cisplatin were obtained from previously reported data run in parallel [43]. Data points denote mean ± SEM. *n* = 3 from three separate experiments where samples were run in triplicate. ** *p* < 0.01, *** *p* < 0.001 and **** *p* < 0.0001 compared with control, as measured by one-way ANOVA.

The mitochondrial membrane potential (MtMP) changes were measured 72 h post-treatment with prodrugs **Pt^IV^PCLB**, **Pt^IV^5CLB**, **Pt^IV^56CLB**, ligand CLB, or cisplatin. The relative fluorescence units (RFU) measured is reported in Figure 8 and Table S5. A significant hypopolarisation in the mitochondrial membrane was induced in HT29 cancer cells for all the platinum complexes including FCCP (carbonyl cyanide 4-(trifluoromethoxy) phenylhydrazone; positive control) at 24 (*p* < 0.0001), 48 (*p* < 0.0001), and 72 h (*p* < 0.0001) compared to the untreated control group. CLB was only reduced slightly at 72 h. Relative to cisplatin, **Pt^IV^56CLB** showed a significant decrease of MtMP at 24 and 48 h (*p* < 0.01) as well as **Pt^IV^5CLB** at 48 h (*p* < 0.05).

### 2.9. Morphological Changes in Microtubule Organisation

**Figure 9:**
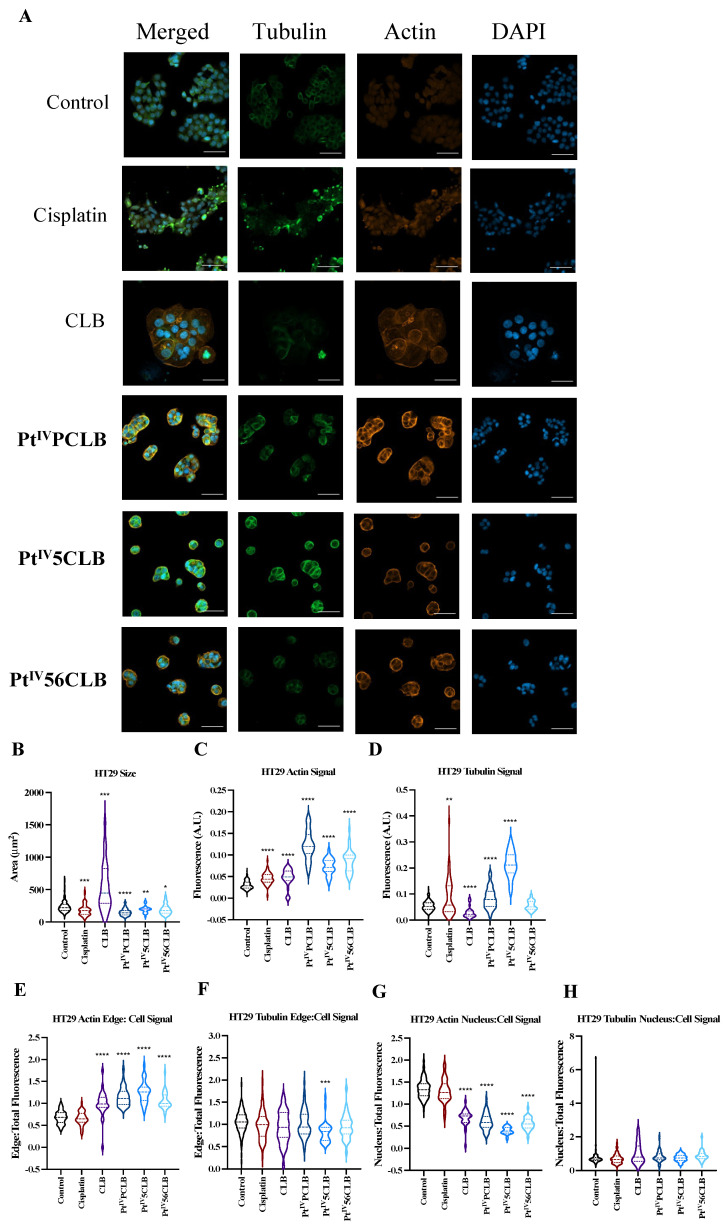
Effects of chlorambucil-platinum(IV) prodrugs, CLB, and cisplatin on β-tubulin and F-actin were measured by immunofluorescence at 72 h post-treatment in HT29 cells as described in Section 4.12. (**A**) HT29 airy scan images at 20×. (**B**) HT29 cell size ($\mu m^{2}$). (**C**) Actin expression in HT29. (**D**) Tubulin expression in HT29. (**E**) Edge/Cell ratio of actin expression in HT29 (**F**) Edge/Cell ratio of tubulin expression in HT29. (**G**) Nucleus/Cell ratio of actin expression in HT29 (**H**) Nucleus/Cell ratio of tubulin expression in HT29. The data for cisplatin were obtained from previously reported data run in parallel [43]. Confocal microscope parameters were constant across all treatments for comparison in expression. Data were analysed from triplicate experiments at *n* = 30 cells per replicate for each treatment. Data points denote mean ± SEM. * *p* < 0.05, ** *p* < 0.01, *** *p* < 0.001 and **** *p* < 0.0001 compared to control, as measured by one-way ANOVA.

HT29 cells were studied by means of confocal microscopy for microtubule changes post 72 h of treatment with the investigated agents, **Pt^IV^PCLB**, **Pt^IV^5CLB**, **Pt^IV^56CLB**, ligand CLB, or cisplatin (Figure 9 and Figure S7). Cell size, tubulin intensity, and actin intensity were measured. Furthermore, the fluorescence signal of actin and tubulin on the edge of the cell as well as the ratio of actin and tubulin in the nucleus to cell expression were measured (Figure 9). Phalloidin staining F-actin exhibited a distinctive aggregation of actin filaments at the cytosol after treatment with **Pt^IV^PCLB**, **Pt^IV^5CLB** and **Pt^IV^56CLB** (Figure 9). Actin total cell expression (Figure 9C) was significantly increased (*p* < 0.0001) across all treatments, which was primarily observed at the edge of the cell (Figure 9E) with considerable increase in, **Pt^IV^PCLB** (*p* < 0.0001), **Pt^IV^5CLB** (*p* < 0.0001), **Pt^IV^56CLB** (*p* < 0.0001) and CLB treatments. Decreased actin nucleus-to-cell ratio in **Pt^IV^PCLB** (*p* < 0.0001), **Pt^IV^5CLB** (*p* < 0.0001), **Pt^IV^56CLB** (*p* < 0.0001) and CLB treatments (Figure 9G) was also observed. The observed actin changes are characteristics observed by apoptotic-induced cell death [76,77,78]. The tubulin total cellular expression was relatively increased in cisplatin (*p* < 0.01), **Pt^IV^PCLB** (*p* < 0.0001), and **Pt^IV^5CLB** (*p* < 0.0001) treatments while interestingly reduced expression in **Pt^IV^56CLB**- and CLB-treated cells was observed (Figure 9A,D). The tubulin signal on the edge of the cell was decreased compared to control (Figure 9F) with no major change to the nuclear-to-cytosolic ratio of tubulin in treated cells (Figure 9H). Subsequently, the HT29-treated cells exhibited morphological changes in the cytoskeleton, exhibiting an increase in aggregation compared to the normal filamentous distribution of actin and tubulin in the untreated (control) cells (Figure 9A and Figure S7). A significant reduction in cell size was observed across the cells treated with the investigated prodrugs—**Pt^IV^PCLB** (*p* < 0.0001), **Pt^IV^5CLB** (*p* < 0.01), **Pt^IV^56CLB** (*p* < 0.05), and cisplatin (*p* < 0.001)—while that of CLB induced an increase in cell size (*p* < 0.001) (Figure 9B). Membrane blebbing was also seen across all investigated agents as indicated using yellow arrows in Figure S7, in all suggesting the activation of apoptotic cell death [76].

### 2.10. Wound Healing Assay

**Figure 10:**
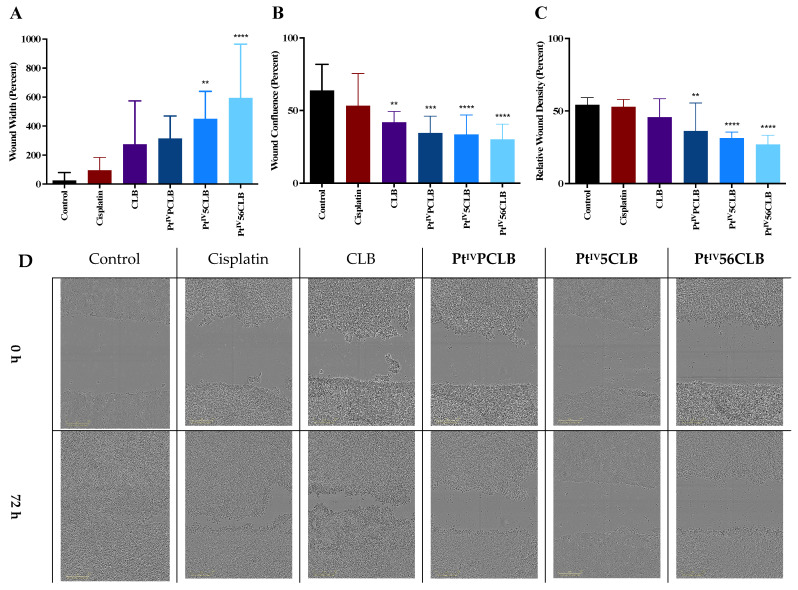
Wound Closure. Percentage wound width (**A**), wound confluence (**B**), relative wound density (**C**), and representative microscopic (10×) images (**D**) measured upon treatment with **Pt^IV^PCLB**, **Pt^IV^5CLB**, and **Pt^IV^56CLB** prodrugs, as well as CLB and cisplatin in HT29 cells up to 72 h as described in Section 4.13. The data for cisplatin were obtained from previously reported data run in parallel [43]. Representative microscopic images are enlarged in Figure S8. Data points denote mean ± SEM. *n* = 3 from three separate experiments where samples were run in triplicate. ** *p* < 0.01, *** *p* < 0.001 and **** *p* < 0.0001 compared with control, as measured by one-way ANOVA.

The first step of tumour metastasis is when cancer cells invade surrounding tissue and vessels, which allows the cancer cells to metastasise chemotactically and adhere to proliferate [79,80,81,82]. A 2D monolayer was wounded and monitored for 72 h post treatment with **Pt^IV^PCLB**, **Pt^IV^5CLB**, **Pt^IV^56CLB**, CLB, or cisplatin (Figure 10D) to understand the effect of these drugs on artificial wound closure. The percentage wound width, wound confluence and relative wound density were measured (Figure 10 and Figure S9). HT29 cells treated with **Pt^IV^PCLB** exhibited significant confluence (*p* < 0.001) and reduced wound density (*p* < 0.05) (Figure 10B,C), also observed in **Pt^IV^5CLB** and **Pt^IV^56CLB** treatments (*p* < 0.0001). An increase in wound width was observed across all treatments with a significant increase in **Pt^IV^5CLB**- (*p* < 0.01) and **Pt^IV^56CLB**- (*p* < 0.0001) treated cells (Figure 10C). Compared to cisplatin, the relative wound density of **Pt^IV^PCLB**, **Pt^IV^5CLB**, and **Pt^IV^56CLB** decreased significantly: *p* < 0.01, *p* < 0.001 and *p* < 0.0001, respectively. Additionally, the wound confluence of **Pt^IV^PCLB**, **Pt^IV^5CLB**, and **Pt^IV^56CLB** was significantly decreased relative to cisplatin: *p* < 0.05, *p* < 0.05 and *p* < 0.01, respectively. The wound width of **Pt^IV^5CLB** and **Pt^IV^56CLB** remained significantly increased compared to cisplatin at *p* < 0.01 and *p* < 0.0001, respectively.

### 2.11. Western Blot

**Figure 11:**
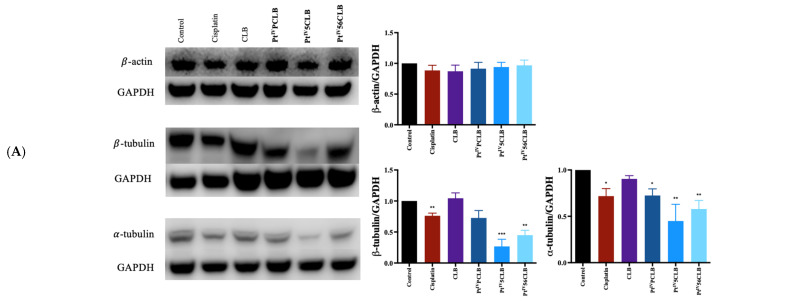

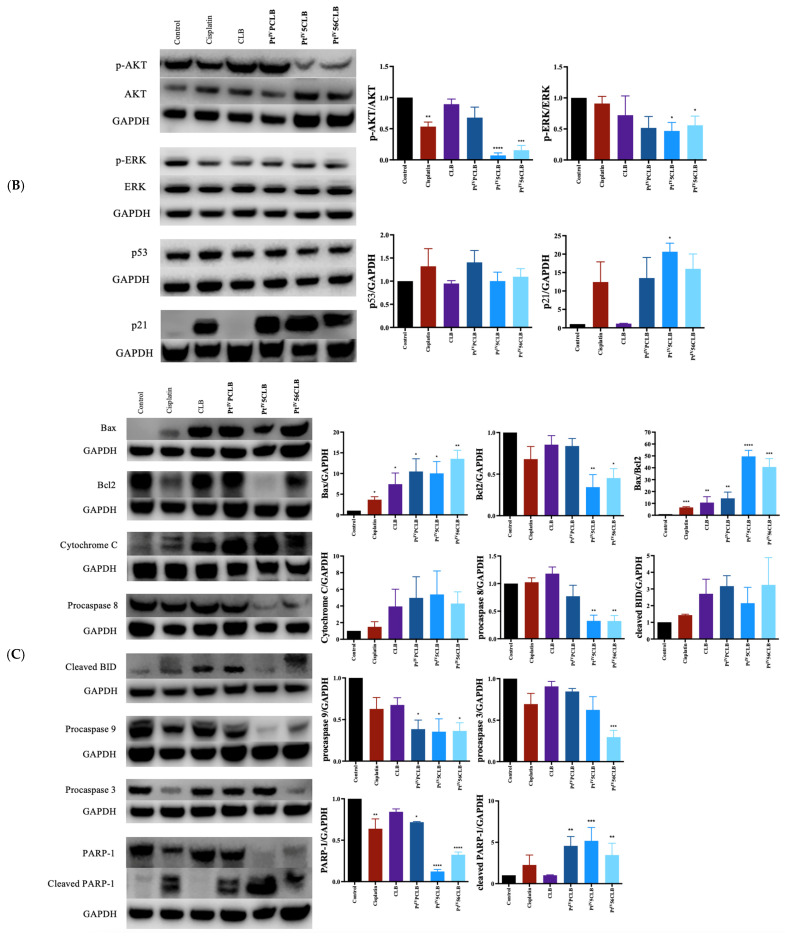

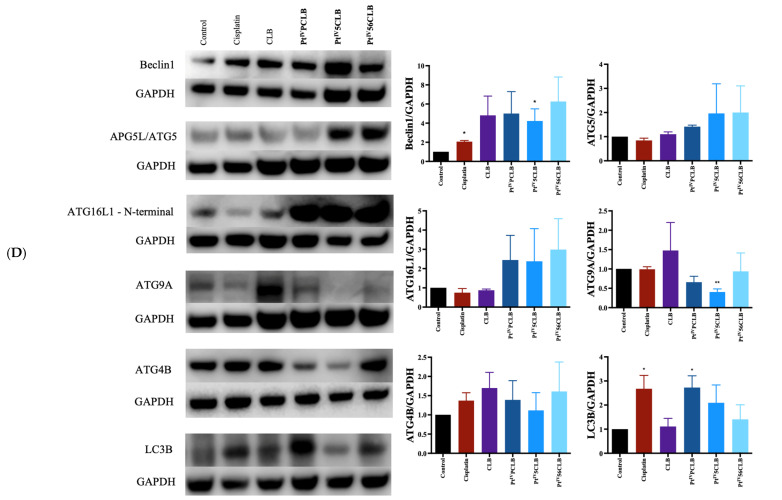
Protein expression upon treatment with **Pt^IV^PCLB**, **Pt^IV^5CLB**, and **Pt^IV^56CLB** prodrugs, as well as CLB and cisplatin in HT29 cells at 72 h as described in Section 4.14. (**A**) Microtubule cytoskeleton markers (**B**) Cell proliferation markers (**C**) Intrinsic and extrinsic apoptotic cell death markers (**D**) Autophagy markers. Data points denote mean ± SEM. *n* = 3 from three separate experiments. * *p* < 0.05, ** *p* < 0.01, *** *p* < 0.001 and **** *p* < 0.0001 compared with control, as measured by unpaired Student’s *t*-test. The full uncropped western blot, with its corresponding molecular markers, is represented in Figures S15–S18.

The protein expression changes in microtubule cytoskeleton, cell proliferation, and key apoptotic and autophagic proteins in HT29 cells were measured post 72 h of treatment with prodrugs **Pt^IV^PCLB**, **Pt^IV^5CLB**, **Pt^IV^56CLB**, ligand, CLB, and cisplatin (Figure 11). Results have shown some modifications to the microtubule cytoskeleton markers with a significant decrease in β-tubulin in **Pt^IV^5CLB** (*p* < 0.001), **Pt^IV^56CLB** (*p* < 0.01), and cisplatin (*p* < 0.01) treatments and in α-tubulin in cisplatin and **Pt^IV^PCLB** (*p* < 0.05), **Pt^IV^5CLB** and **Pt^IV^56CLB** (*p* < 0.01) treatment (Figure 11A). Cellular proliferation marker expression was also affected by prodrug treatment (Figure 11B). A significant reduction in p-AKT/AKT in **Pt^IV^5CLB** (*p* < 0.0001) and **Pt^IV^56CLB** (*p* < 0.001) treatments was observed compared to cisplatin (*p* < 0.01) in addition to a decrease in p-ERK/ERK across **Pt^IV^5CLB** and **Pt^IV^56CLB** (*p* < 0.05). An increase in p53 was observed in cisplatin and **Pt^IV^PCLB**-treated cells while p21 showed an increase in cisplatin and all prodrugs with significance in **Pt^IV^5CLB** (*p* < 0.05) treatment. The mitochondrial intrinsic and extrinsic mechanism of cell death was studied (Figure 11C). The pro-apoptotic marker BAX was increased in all treatments with significance while the expression of the anti-apoptotic marker Bcl2 was decreased with significance in **Pt^IV^5CLB** (*p* < 0.01) and **Pt^IV^56CLB** (*p* < 0.05), which exhibited a significant increase in Bax/Bcl-2 in cisplatin, CLB and **Pt^IV^PCLB** (*p* < 0.01), **Pt^IV^5CLB** (*p* < 0.0001), **Pt^IV^56CLB** (*p* < 0.001), and cisplatin (*p* < 0.001), indicating a pro-apoptotic mechanism. Cytochrome c was notably increased across all treatments. Procaspase 8 was significantly decreased in **Pt^IV^5CLB** and **Pt^IV^56CLB** (*p* < 0.01) with an observed increase in cleaved BID in all prodrugs suggesting the extrinsic pathway to cell death is activated. The executionary caspases 9 and 3 were also active indicated by a reduction in expression across all treatments in **Pt^IV^PCLB**, **Pt^IV^5CLB**, and **Pt^IV^56CLB** (*p* < 0.01) in procaspase 9 and in **Pt^IV^56CLB** (*p* < 0.001) in procaspase 3. The levels of PARP-1 were reduced across all treatments with a significant reduction in **Pt^IV^PCLB** (*p* < 0.05), **Pt^IV^5CLB** (*p* < 0.0001), **Pt^IV^56CLB** (*p* < 0.0001), and cisplatin (*p* < 0.01) and cleaved PARP-1 was also observed to increase across all treatments with a significant increase in **Pt^IV^PCLB** (*p* < 0.01), **Pt^IV^5CLB** (*p* < 0.001), and **Pt^IV^56CLB** (*p* < 0.01). Markers of autophagy were also studied in HT29-treated cells (Figure 11D). Beclin-1 was increased across all treatments with significance observed in the cisplatin and **Pt^IV^5CLB** (*p* < 0.05) treatment. ATG5 and ATG16L1 expression levels were observed to increase with prodrug treatments. Meanwhile, ATG9A showed a reduced expression in prodrug treatments with significance in **Pt^IV^5CLB** (*p* < 0.01). ATG4B showed an increase in CLB and **Pt^IV^56CLB** treatments. The levels of LC3B were significantly increased in cisplatin- (*p* < 0.05) and **Pt^IV^PCLB**- (*p* < 0.05) treated cells, while still showing an increase in **Pt^IV^5CLB** and **Pt^IV^56CLB**.

### 2.12. Proteomics

**Figure 12:**
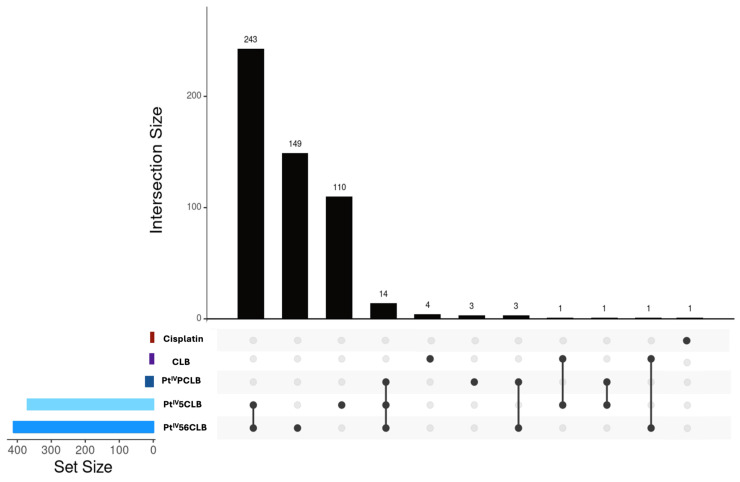
Differential proteins upon treatment with **Pt^IV^PCLB**, **Pt^IV^5CLB**, **Pt^IV^56CLB** prodrugs, CLB, and cisplatin in HT29 cells at 72 h. UpSet plot summarises the prodrugs and ligands differential protein expression analysis. The total number of proteins with variations in the log 2-fold change expression for each complex is displayed in the horizontal bar graph located at the bottom left of each picture. Joined black circles to the right of these bar graphs indicate that the same differentially expressed proteins were common to the complex comparisons shown on the left. The number of proteins with comparable log 2-fold change expression differences in the drug comparisons is quantified in the vertical bar graph at the top.

**Figure 13:**
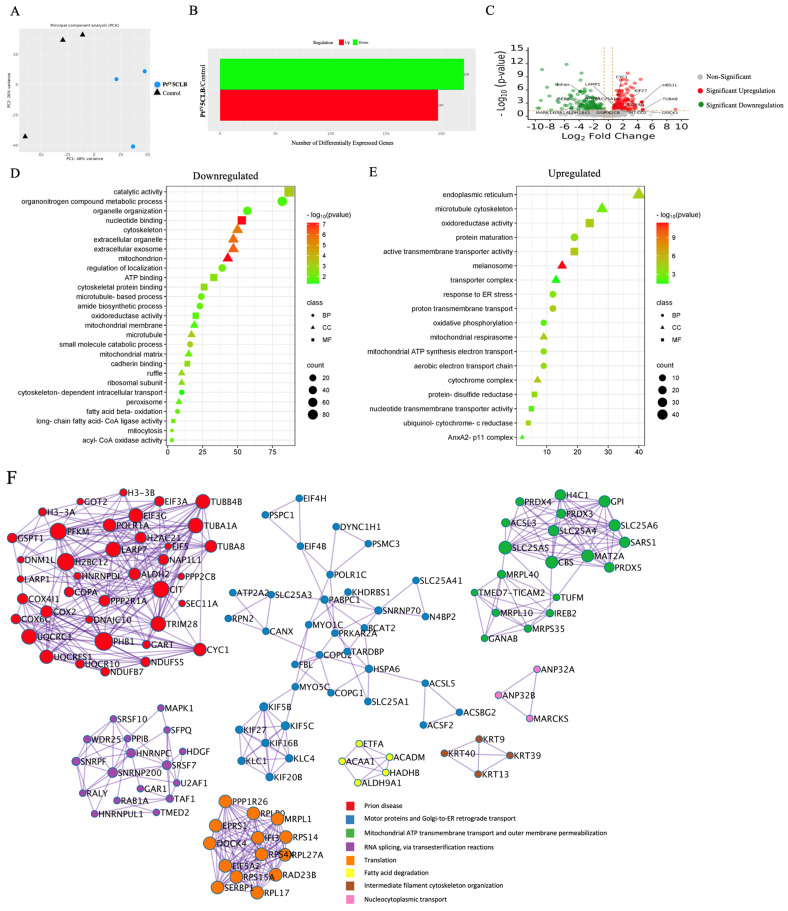
Proteomic analysis of HT29 upon treatment with **Pt^IV^5CLB**. (**A**) HT29 principal component analysis. (**B**) HT29 number of differentially expressed proteins (DEPs). (**C**) HT29 volcano plot of DEPs upregulated (red) and downregulated (green). GO enriched biological processes, cellular components, and molecular function in HT29, (**D**) Downregulated, and (**E**) Upregulated proteins. (**F**) HT29 pathway enrichment and gene act network analysis in the most significant of dysregulated pathways. Data points denote mean ± SEM. *n* = 3 from three separate experiments. For clarity, a larger representation is provided in the Supplementary Figure S12.

**Figure 14:**
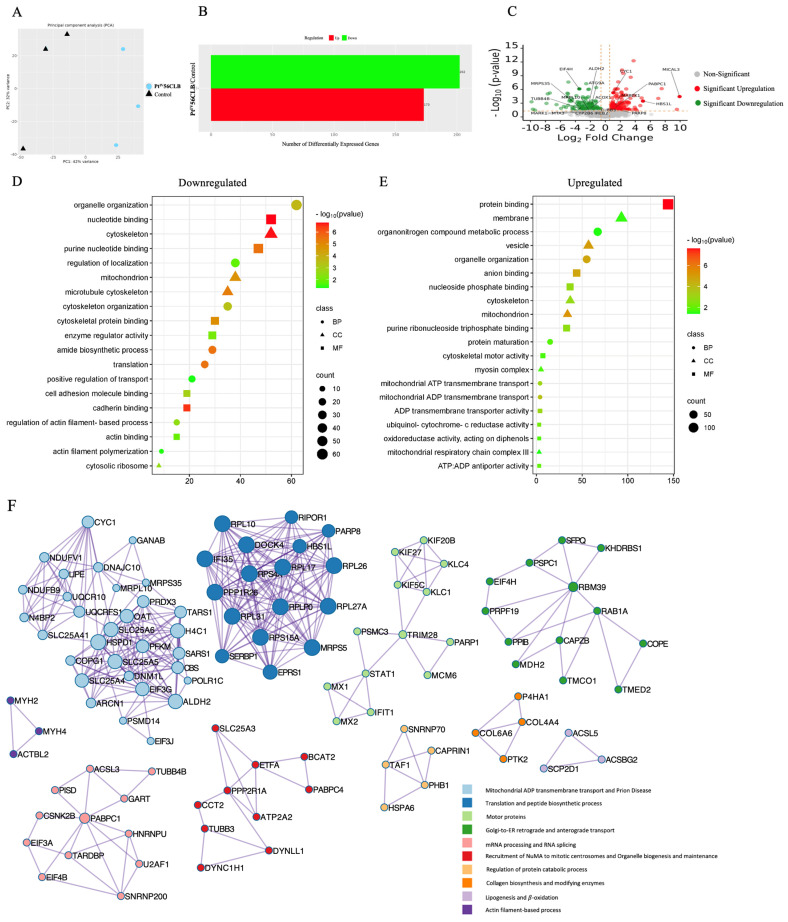
Proteomic analysis of HT29 upon treatment with **Pt^IV^56CLB**. (**A**) HT29 principal component analysis. (**B**) HT29 number of differentially expressed proteins (DEPs). (**C**) HT29 volcano plot of DEPs upregulated (red) and downregulated (green). GO enriched biological processes, cellular components, and molecular function in HT29, (**D**) Downregulated, and (**E**) Upregulated proteins. (**F**) HT29 pathway enrichment and gene act network analysis in the most significant of dysregulated pathways. Data points denote mean ± SEM. *n* = 3 from three separate experiments. For clarity, a larger representation is provided in the Supplementary Figure S13.

**Figure 15:**
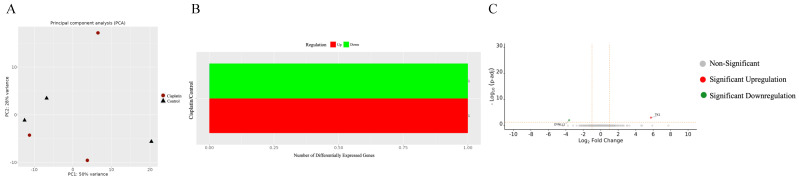
Proteomic analysis of HT29 upon treatment with cisplatin. (**A**) HT29 principal component analysis. (**B**) HT29 number of differentially expressed proteins (DEPs). (**C**) HT29 volcano plot of DEPs upregulated (red) and downregulated (green). Data points denote mean ± SEM. *n* = 3 from three separate experiments. For clarity, a larger representation is provided in the Supplementary Figure S14.

The global proteome profile in HT29 cells treated with IC_50_ concentration (Table 1) of each investigated agent was studied at 72 h using quantitative proteomic analysis (Figure 12, Figure 13, Figure 14 and Figure 15). In total, 1859 HT29 proteins were identified across all paired samples that passed quality control in all three replicates of each drug treatment. Initially, principal component analysis (PCA) was done to screen and compare the proteome profiles among treated vs. untreated cells for further analysis. PCA plots in Figure 13A, Figure 14A and Figure 15A show the treated samples are separated, indicating overall differences in proteome expression profiles in treated samples compared to control. The number of up- and downregulated proteins are shown across all the treatments (Student’s unpaired *t*-test, *p* < 0.05, fold change > 2) (Figure 13B, Figure 14B and Figure 15B). Of all the investigated agents, **Pt^IV^5CLB** and **Pt^IV^56CLB** expressed significant dysregulation in proteins post-treatment supporting the high potency of these prodrugs. The **Pt^IV^PCLB** (Figure S11), ligand CLB (Figure S10), and cisplatin (Figure S15) did not exhibit a similar effect to **Pt^IV^5CLB** and **Pt^IV^56CLB** at 72 h post treatment. Cisplatin-treated HT29 cells did not have many differently expressed proteins, TK1 was upregulated and DYNLL1 was downregulated (Figure 15C).

Notably, MAP2K1 and CYC1, markers of proliferation and mitochondrial-mediated cell death, were upregulated in **Pt^IV^5CLB**- and **Pt^IV^56CLB**-treated HT29 cells, while MAPK1 was downregulated (Figure 13C and Figure 14C). Furthermore, cytoskeletal organization, RNA splicing, biosynthetic processes, and translation were dysregulated in **Pt^IV^5CLB** and **Pt^IV^56CLB** treatments (Figure 13D and Figure 14D).

The number of dysregulated proteins affected by treatment, from highest to lowest, was **Pt^IV^5CLB** (Figure 13B) > **Pt^IV^56CLB** (Figure 14B) > **Pt^IV^PCLB** (Figure S11) > CLB (Figure S10) > cisplatin (Figure 15B). Differentially expressed proteins (DEPs) identified as intersecting across all treatments in HT29 are reported in an UpSet plot (Figure 12). There were 14 DEPs common to all treatments with prodrugs. There were 243 DEPs shared between **Pt^IV^5CLB**- and **Pt^IV^56CLB**-treated cells. **Pt^IV^56CLB** treatment resulted in the most DEPs with 149 > **Pt^IV^5CLB** with 110 DEPs > CLB with 4 DEPs > **Pt^IV^PCLB** with 4 DEPs > cisplatin with 1 DEP when used alone.

Next, based on GO annotations, the functional ontology enrichment analysis of drug-specific up- and downregulated proteins was examined. Figure 13D,E and Figure 14D,E show the findings from each of the three GO biological processes, molecular function, and cellular components. GO analysis identified that the downregulated pathways across **Pt^IV^5CLB** and **Pt^IV^56CLB** treatments in HT29 were linked to microtubule cytoskeleton, organelle organisation, mitochondrial matrix, nucleotide binding, and biosynthetic processes (Figure 13D and Figure 14D). Mitochondrial ATP synthesis electron transport, cytochrome complex, and oxidoreductase activity were increased commonly in **Pt^IV^5CLB** and **Pt^IV^56CLB** treatments (Figure 13E and Figure 14E).

## 3. Discussion

The biological mechanisms of the novel CLB-platinum(IV) prodrugs were investigated. It has recently been demonstrated that these prodrugs exhibit antineoplastic potency on a number of different cancers including colorectal, breast, lung, skin and brain [24]. Investigating the distinct mechanisms of action of the prodrugs on cellular death is essential to inform future in vivo research. Cytotoxicity was again investigated on HT29 cancer cells and MCF10A non-tumourigenic immortalised human breast epithelial cells, showing the same trend in response across the complexes as previously observed [24]. **Pt^IV^56CLB** was most potent with observed cytotoxicity in decreasing order > **Pt^IV^5CLB** > **Pt^IV^PCLB** > cisplatin > CLB (Figure 1 and Table 1).

Platinum(IV) complexes can circumvent the standard harmful effects of platinum(II) complexes by preferentially reducing and activating the prodrug intracellularly in hypoxic and low-pH milieus of cancer cells [32,83,84,85]. Non-tumourigenic immortalised human breast epithelial cells (MCF10A) were used to test the selectivity of each complex to cancer cells (Table 1 and Table 2). The SCI was higher in **Pt^IV^PCLB**, **Pt^IV^5CLB**, and **Pt^IV^56CLB** despite the IC_50_ being in the low micromolar range, which suggests the investigated agents have a better selectivity to cancer cells, as depicted by their greater SCIs which ranged from 3 to 8 compared to cisplatin and CLB which ranged from 0 to 1. The cytotoxic effect of the complexes was then investigated in 3D bioprinted HT29 spheroids, for a better understanding of translated drug response in vivo. Although the cytotoxicity of the platinum prodrugs decreased in HT29 spheroids compared to 2D monolayer HT29, as expected, it was still more effective than cisplatin (>24-fold) (Table 1). Additionally, the order of potency remained the same for the prodrugs with **Pt^IV^56CLB** most potent with observed cytotoxicity in decreasing order > **Pt^IV^5CLB** > **Pt^IV^PCLB**, while that of CLB proceeded cisplatin in potency on HT29 spheroids.

At 3 µM, the cellular uptake of the platinum complexes was evaluated (Figure 2 and Figure S3, and Table S3). The CLB-platinum(IV) prodrugs all exhibited increased uptake compared to their platinum(II) precursors and platinum(IV) scaffolds as previously observed [43]. Compared to cisplatin, **Pt^IV^PCLB**, **Pt^IV^5CLB**, and **Pt^IV^56CLB** were significantly increased at 30 h and depicted a high intracellular/extracellular ratio (Table S3). **Pt^IV^56CLB** showed the greatest mean cell concentration after 3 h of treatment and the highest uptake at 30 h of treatment compared to **Pt^IV^PCLB** and **Pt^IV^5CLB**. The significant cytotoxicity observed by the **Pt^IV^56CLB** prodrug compared to cisplatin may be explained, at least in part by increased cellular uptake. While **Pt^IV^5CLB** demonstrated increased cell uptake it was less than that of **Pt^IV^56CLB** (Figure 2). The cell uptake of the prodrugs is kinetically time-dependent which explains the continual increase in uptake without a plateau except for that observed in **Pt^IV^PCLB** at 24–30 h. This is rationalised by the platinum prodrugs’ cytotoxic effect and mechanism analysis at 72 h, where reductive activation of the prodrugs is required for activity [24]. The high intracellular/extracellular ratio indicates that the prodrugs investigated use an active mechanism of transport (Table S3).

It is plausible that platinum prodrugs would be cellularly absorbed by means of many transport mechanisms, including endocytosis, passive or facilitated diffusion and active transport via transport proteins that need ATP for energy [86,87]. The transport mechanism was studied by quantifying the drug uptake via ICP-MS after inhibiting the mechanism of interest and comparing it to optimal conditions at 37 °C. Active transport was investigated by inhibiting active mechanisms of transport at 4 °C [88]. The CLB-platinum(IV) prodrugs exhibited reduced uptake at 4 °C indicating the use of active transport for cellular drug uptake. Despite that, the intracellular concentration at 4 °C was much lower than that observed by the optimal conditions; for all prodrugs examined, it was still 92-fold higher than the extracellular concentration, suggesting the presence of additional transport mechanisms, compared to optimal conditions [89,90]. After preventing clathrin-mediated endocytosis, there was also a notable reduction in cell uptake with **Pt^IV^PCLB** (*p* < 0.01), **Pt^IV^5CLB** (*p* < 0.05), **Pt^IV^56CLB** (*p* < 0.001), and cisplatin (*p* < 0.01) indicating the prodrugs can be internalised by clathrin-mediated endocytosis, as has been previously reported for cisplatin [91]. The SLC7A5 alias LAT1 receptor is upregulated in cancer cells because proliferation requires nutrients such as amino acids [92,93,94]. SLC7A5 receptor uptake was reduced across all treatments after blocking this route with a significant reduction in cisplatin treatment and **Pt^IV^56CLB**. TfR is a membrane receptor that binds to the primary iron-carrying protein, transferrin, to control the quantity of iron that enters the cell. Iron is highly essential for the growth of cancer cells [95,96,97]. Although blocking the transferrin receptor showed some reduced amount of prodrugs in HT29 cells, it was not as evident as previously reported with their platinum(II) precursors and platinum(IV) scaffolds [43]. This suggests that the prodrugs could prefer another active transport mechanism in HT29 cells. According to this study, there may be more than one intracellular pathway that the platinum complexes under investigation use, with active transport serving as the main means of cell uptake (Figure 3).

To investigate the build-up of platinum in the nuclear, cytoskeletal, membrane/particulate, and cytosolic fractions, the cellular localisation experiment (Figure 4) was undertaken. The platinum prodrugs investigated were profoundly localised to the cytoskeleton when compared to all other fractions; this correlates with previous observations with the platinum(II) precursors and platinum(IV) scaffolds [43] and is different to cisplatin, which localises mostly in the membrane/particulate fraction [43,98]. This implies that the platinum complexes investigated may attach to filamentous cytoskeletal proteins, disrupting the structural integrity of the cell and ultimately leading to apoptosis. The observed buildup of the investigated agents in the nucleus may be connected to non-covalent interaction with DNA [99].

Flow cytometry was utilised to determine the percentage of cell death positive or negative for Annexin-V and PI staining in order to evaluate the mechanism of cell death connected to the cytotoxicity of each complex at 72 h. The assay is dependent on the translocation of phosphatidylserine (PS) to the outer membrane leaflet, which occurs in both the early and late stages of apoptosis [100,101]. Furthermore, cells in necrosis and late apoptosis (also known as post-apoptotic necrosis) lose their membrane integrity, making them more permeable to the DNA-binding PI stain [102,103]. The cells are divided into four quadrants by the flow sorter. Cells that have undergone early apoptosis in quadrant 3 will be positive for only Annexin-V, but the percentage of viable cells in quadrant 4 will be negative for both Annexin-V and PI. Necrotic cells will be PI positive and Annexin-V negative in quadrant 1, while cells that have undergone late apoptosis will be positive for both markers in quadrant 2 (Figure 5). There was a noticeable decline in viable cells after 72 h of treatment for all regimens (Figure 5A,B). Early and late apoptotic cells were notably increased across treatments in **Pt^IV^PCLB**, **Pt^IV^5CLB**, and **Pt^IV^56CLB**, which was comparable to that previously observed in HT29-treated cells with platinum(II) precursors greater than those observed by the platinum(IV) scaffolds, supporting the reductive activation of the prodrugs to their platinum(II) precursors. An increase in necrotic cells was also observed across all treatments with most produced by CLB treatment in HT29 cells. Subsequently, the cell cycle arrest profile was studied (Figure 6). A decrease in G0/G1 phase was observed with a notable increase in the S phase of the cell cycle across the investigated prodrugs, similarly observed by their relative platinum(II) precursor [43]. Meanwhile, cisplatin-treated cells remained predominately in the G0/G1 phase. Cell death may depend on signalling for DNA damage, as suggested by cellular arrest in the S phase [104].

ROS production was studied to reassess its activity at the observed IC_50_ (Table 1) at 72 h (Figure 7) compared to previous observations at their GI_50_ concentration [24]. ROS inhibitors like NAC were investigated in the previous study to ensure the method implemented did not lead to cell death [24]. Here, we measured the production of ROS in response to IC_50_ (Table 1) treatment with the investigated agents. As previously reported, the production of ROS across all prodrugs was significant at 24, 48, and 72 h at their IC_50_ concentration [24]. As a result of excessive cellular ROS, inducing damage to proteins, lipids, organelles, and nucleic acids, this supports the theory that the mechanism of cell death is linked to apoptosis [105]. ROS-inducing anticancer drugs exploit cancer cells’ heightened sensitivity to oxidative stress, aiming to elevate ROS levels selectively within tumours to toxic thresholds while minimizing damage to healthy cells [106,107]. Despite efforts to mitigate the impact on healthy tissues, some level of influence remains due to ROS’s inherent nature and the systemic effects of the drugs [108,109,110]. Understanding the potential induction of ROS and the differing sensitivities of cancer versus normal cells is crucial for optimising treatments and reducing unintended consequences [110,111]. It has been demonstrated that metal complexes generate hydroxyl radicals and ROS, which result in oxidative DNA adducts [112,113,114]. Previously reported ROS production by cisplatin has been linked to the disturbance of the MtMP [56,115,116]. The mitochondria, the cell’s engine, provide energy for metabolic processes that keep cells functioning normally [117]. Chemotherapeutics, including metallodrugs, cause disruption in the MtMP which triggers the activation of the intrinsic mitochondrial apoptotic pathway [118]. One of the most important indicators of a bioenergetic cell stressor, MtMP, reflects the electrical potential difference between a cell’s extracellular and intracellular surroundings [119,120]. Our platinum(II) and (IV) complexes, scaffolds for the prodrugs, exhibited a significant reduction in MtMp in HT29 cells, as previously reported [43]. In treated HT29 cells, the MtMP was considerably decreased at 24, 48, and 72 h for all prodrugs including their ligand CLB (Figure 8).

Actin and tubulin fluorophore antibodies were used in immunofluorescence to further examine the impact of the prodrugs on the microtubule cytoskeleton (Figure 9). Cell morphology changes were seen in the HT29-treated cells (Figure 9A), including cell shrinkage, membrane blebbing and cytoskeletal collapse, which are all characteristics of apoptotic cell death occurring [121]. All investigated prodrugs exhibited a significant decrease in HT29 cell size, including cisplatin while CLB showed an increase in cell size (Figure 9B and Figure S7) that may be indicative of necrosis, which includes a characteristic of cell swelling [122]. This is confirmed by the increase in necrotic cells observed after treatment with CLB by Annexin V/PI using flow cytometry (Figure 5B). Actin plays a role in controlling the breakdown of DNA during apoptosis. The cleavage of caspase 3 causes membrane blebbing, which is mediated by the actin-myosin complex [78,123]. The observed changes in phalloidin (F-actin) expression after treatment with the investigated agents support the proposed mechanism. Its intensity is significantly increased at the edge of the cell (Figure 9C,E and Figure S7). Tubulin has been shown to also contribute to cellular stress responses [124]. α and β-tubulin heterodimers make up microtubules which are necessary for mitosis, intracellular trafficking, and cell motility. This makes tubulin a target for chemotherapeutic agents. Anti-microtubule agents can induce damage to the mitotic spindle triggering mitotic arrest (G2+M phase) and subsequently cell death [124,125,126]. A filamentous distribution of tubulin was observed in the control HT29 cells, while treated cells exhibited changes in the architecture of the microtubule network. An increase of tubulin aggregates at the perinuclear region and at the edge of the cell was observed and this indicated increased tubulin expressed in the treated cells relative to the control (Figure 9D). This has previously been demonstrated with microtubule targeting agents, which suggests the prodrugs have the ability to alter microtubule dynamics, which implies a G2+M arrest may also be induced as previously reported for the **Pt^II^56ME*SS*** platinum(II) precursor in MDA-MB-231 cells [56,57,127,128,129].

The earliest stage of tumour metastasis is characterised by the ability of cancer cells to spread by chemotaxis, infiltrating nearby tissues and blood vessels while being guided by protrusive activity from the cell membrane, adhering to the extracellular matrix (ECM) [130,131,132]. Therefore, the prodrugs under study were used to examine the spread and adhesive capabilities of HT29 cells in comparison to the untreated control group (Figure 10 and Figure S9). At 72 h post treatment, the wound width remained significantly wide in **Pt^IV^5CLB** and **Pt^IV^56CLB**, with reduced confluency across all treatments, showing the cells’ reduced ability to close the artificial wound in monolayer, which is associated with their decreased propensity to spread.

Western blot analysis was used to evaluate the expression of important apoptotic, cell proliferation, microtubule, and autophagy markers in HT29 cells 72 h after treatment to investigate the pathways involved in cell death (Figure 11). All protein markers were normalised to GAPDH and to their relative no treatment control per gel. The intrinsic mitochondrial route leading to apoptosis is significantly influenced by proteins belonging to the Bcl2-family, Bax the pro-apoptotic, and Bcl2 the anti-apoptotic marker. Bcl2 levels drop and Bax is released when this route is activated. Bax permeabilises the mitochondrial membrane and releases cytochrome c, another protein that is elevated in intrinsic apoptosis [122,133,134]. All treatments in HT29 exhibited an increase in Bax/Bcl2 ratio indicating a proapoptotic mechanism with a prominent use by **Pt^IV^5CLB**- and **Pt^IV^56CLB**-treated cells (Figure 11C). Cytochrome c also increased across all treatments indicating its release from the mitochondria.

Transmembrane receptor-mediated interactions, which include death receptors belonging to the tumour necrosis factor (TNF) family and protein indicators like caspase 8 are part of the extrinsic signalling route that initiates programmed cell death. BID is cleaved by caspase 8 activation, which then travels to the mitochondria and activates Bax [135]. HT29 treated with the prodrugs showed a reduced expression in procaspase 8 compared to control with a significant decrease in **Pt^IV^5CLB** and **Pt^IV^56CLB**, suggesting the activation of caspase 8 forming cleaved caspase 8 which activates BID. This was observed by a detected increase in BID expression including all treatments compared to the control.

The markers of the executionary pathway are shared by the intrinsic mitochondrial and extrinsic pathways. These markers include executionary caspases 9 and 3, which are activated to cleaved form, holding effector activity to downstream proteins, such as activation of PARP-1 (Poly[ADP-ribose] polymerase 1), whose downstream cleavage is linked to an increase in DNA fragmentation in the nucleus, triggering apoptosis in cells [133,136,137,138]. Western blot quantifications have exhibited a notable decrease in procaspase 3 and 9 in the prodrug-treated cancer cells with a significant decrease of procaspase 9 in **Pt^IV^PCLB**-, **Pt^IV^5CLB**-, and **Pt^IV^56CLB**-treated cells and a decrease in procaspase 3 in **Pt^IV^56CLB**. PARP-1 activity was also notably decreased across all prodrug treatments and cisplatin with an increase in cleaved PARP-1, with significance in **Pt^IV^PCLB**-, **Pt^IV^5CLB**-, and **Pt^IV^56CLB**-treated cells. Based on these data, the CLB-platinum(IV) prodrugs trigger both the mitochondrial intrinsic and extrinsic signal transduction mechanism of programmed cell death.

Additionally, p53 and p21, tumour suppressor proteins, were studied (Figure 11B). p53 accumulates in the nucleus in response to DNA damage, which sets off the transcription of p21 and other response genes. This accumulation of p21 in the nucleus activates the DNA-damage cell cycle checkpoints G1/S, which can lead to cell cycle arrest through cyclin-dependent kinases [139,140]. Both p53 and p21 were quantified with increased expression in HT29-treated cells. A notable increase in p53 was observed in cisplatin and **Pt^IV^PCLB** treatments. All investigated agents exhibited a distinguished increase in p21 except for the ligand CLB.

The ability to control cellular invasion and migration as well as the survival of cells has been associated with the PI3K-AKT and Ras-ERK cell proliferation pathways [141,142]. Effectors involved in apoptosis are linked to downstream effectors in both pathways. p21, Bax, and Bid are the substrates that ERK regulates. When ERK (p-ERK) is phosphorylated and activated, it translocates to the nucleus and initiates the transcription of survival genes [143,144,145]. Thus, blocking p-ERK is associated with pro-apoptotic protein activity, with p-ERK remaining in the cytoplasm [145,146]. Additionally, the generation of ROS has been linked to DNA damage, rising p53, and p21 activity, all of which are ERK-regulated [147,148]. The p-ERK/ERK quantification in HT29-treated cells was reduced across all treated cells. By phosphorylating BAX’s Ser184 site to hinder its pro-apoptotic effect and BAD’s (Bcl2-associated death promoter) Ser136 site to cause it to separate from Bcl2, AKT effectively inhibits apoptosis and stops the release of cytochrome c from the mitochondria [149,150]. Together with the inhibition of MDM2, an oncoprotein that downregulates p53, phosphorylated activation of AKT also contributes to the reduction of caspase 9 activity [151]. The p-AKT/AKT quantification in HT29-treated cells was significantly decreased in **Pt^IV^5CLB**- and **Pt^IV^56CLB**-treated cells.

Microtubule cytoskeletal protein markers were further studied using Western Blot analysis (Figure 11A). β-actin expression was not impacted; however, a reduced protein expression was observed in α-tubulin and β-tubulin markers across all treatments with significant reduced expression in α-tubulin in **Pt^IV^5CLB**- and **Pt^IV^56CLB**-treated cells. This further suggests the prodrugs have the ability to alter microtubule dynamics, as previously reported by the **Pt^II^56ME*SS*** platinum(II) precursor in MDA-MB-231 cells [57].

Another mechanism of cell death that the prodrugs may be inducing is autophagy as has been observed previously in their platinum(II) precursors (Figure 11D) [43]. Lysosomes disassemble unneeded proteins and organelles during the catabolic cellular process known as autophagy. The suppression of autophagy-linked cell death is associated with the Bcl2 complex and Beclin-1, an autophagy marker. The complex is open to crosstalk between autophagy and apoptosis when Bcl2 is downregulated [152]. AKT, which phosphorylates and obstructs TSC1/2, which stimulates mTOR and cellular survival, is connected to autophagy signal pathways. Thus, the suppression of PI3K signalling pathways suggests that mTOR may have been suppressed, activating markers associated with autophagy [153,154]. The core proteins of ATG (autophagy-related proteins) are classified into functional classes. The components of the class III phosphatidylinositol 3-kinase complex include ATG14, PIK3C3/VPS34, PIK3R4/p150/VPS15, and BECN1/Beclin-1. The proteins ATG7, ATG10, ATG12, ATG16L1, and ATG5 are part of the ATG12 conjugation system. The ATG4, ATG7, ATG3, WIPI2 proteins, and the LC3 protein family are components of the microtubule-associated protein 1 light chain 3 (LC3) conjugation system. In addition, the ATG9 trafficking system, includes markers, WIPI4, ATG2A and ATG2B, and the transmembrane protein; ATG9A [155,156,157]. ATG are key markers of autophagy. ATG5 crosstalks with the apoptosis pathway [155,158]. ATG5 is involved in both the creation of vesicles during autophagy and the subsequent monitoring of the quality of the mitochondria following oxidative damage and cellular lifespan. Together with other ATGs, ATG5 forms a complex with ATG16L1 that directs the lipidation and synthesis of additional autophagy-related proteins [159]. ATG9A also regulates the finer points of directed migration, lipid utilisation from lipid droplets, and cell protrusions [160]. The interaction of ATG4B with LC3B directs the autophagosome to fuse with lysosomes [161,162,163]. The relationship between ATG4B and Bcl2 is also crucial for apoptosis and autophagy crosstalk, as it reduces Bcl2 expression and increases ATG4B after mitochondrial injury. Moreover, ATG4B may dissociate the Beclin-Bcl2 complex to initiate autophagy [164,165]. Beclin-1 expression was increased in all treatments compared to control with significance in **Pt^IV^5CLB** and cisplatin. Noting the significant decrease in Bcl2 for **Pt^IV^5CLB** treatment may suggest that apoptosis and autophagy crosstalk is present. While no significant changes were observed in APG5L/ATG5 protein **Pt^IV^5CLB** and **Pt^IV^56CLB** were most increased. ATG16L1 was most increased by the prodrugs **Pt^IV^PCLB**, **Pt^IV^5CLB**, and **Pt^IV^56CLB**, which may indicate the activation of biosynthetic processes for autophagy. ATG9A marker was not changed compared to control; however, notably, reduced expression was observed in **Pt^IV^5CLB** treatment. ATG4B expression was not significantly changed but exhibited an increase in CLB and **Pt^IV^56CLB**. LC3B exhibited an increase in expression compared to control with significance observed in cisplatin- and **Pt^IV^PCLB**-treated cells.

These CLB-platinum(IV) prodrugs exhibit an overall multi-mechanistic potential that could be helpful in reducing cancer therapy resistance. Combination medications with various modes of action have been shown to improve therapeutic efficacy in clinical settings. Thus, delivering a combination treatment in one entity as a prodrug may be successful in using either mechanistic pathway of cell death and avoiding development of resistance [166,167].

The proteome profile of all investigated agents was studied. Across all treatments, each PCA plot identified differences relative to control. The most potent prodrugs were identified to be **Pt^IV^5CLB** and **Pt^IV^56CLB**, given the observed high number of dysregulated proteins. Meanwhile, **Pt^IV^PCLB** did not exhibit a similar effect at 72 h of treatment similar to the ligand CLB and cisplatin. It is understood that the main mechanism via which traditional platinum complexes induce apoptotic cell death is transcriptional suppression [113]. TK1 is a frequently increased gene associated with a marker of DNA damage and observed in cisplatin-treated cells [168]. Notably, MAP2K1 was upregulated, while MAPK1 was downregulated in both **Pt^IV^5CLB** and **Pt^IV^56CLB** in HT29-treated cells which is consistent with the inhibited effect identified by western blot analysis and indicates its activation in response to cellular stress [169]. CYC1 and PARP1 were significantly upregulated in **Pt^IV^5CLB** and **Pt^IV^56CLB** treatments, which was also observed by western blot analysis. This confirms the ability of these prodrugs to induce intrinsic apoptotic cell death. Microtubule-associated markers such as TUBA8, TUBB3, and TUBB4B, were significantly dysregulated in HT29 **Pt^IV^5CLB**- and **Pt^IV^56CLB**-treated cells, supporting the inhibitory effect of alpha and beta tubulin identified in western blot analysis. Furthermore, GO annotations summarise the drug-specific up- and downregulated protein-associated functions. It further confirms the dysregulation observed in the microtubule cytoskeleton with a decrease in function. Mitochondrial function was also decreased in addition to organelle organization and biosynthetic processes. In response, increased activity was observed in cytochrome complex, mitochondrial ATP synthesis electron transport, and oxidoreductase activity in **Pt^IV^5CLB** and **Pt^IV^56CLB** treatments. Protein network analysis identify overall dysregulated pathways of **5CLB** and **56CLB** treatments in HT29, which again included mitochondrial transport disfunction, outer membrane permeabilisation, RNA splicing, translation, actin and intermediate cytoskeletal filament organization, protein catabolic and peptide biosynthetic processes. This suggests a subsequent effect observed in response to cellular damages induced by **Pt^IV^5CLB** and **Pt^IV^56CLB** treatments activating ROS induced DNA damage [170,171]. **Pt^IV^56CLB** treatment exhibited most DEPs of 149 in HT29 cells distinguishing it as the most potent multi-mechanistic complex for chemotherapy.

## 4. Materials and Methods

### 4.1. Chemicals and Reagents

Dulbecco’s Modified Eagle’s Medium (DMEM), (with 4.5 g/L glucose/L-glutamine/sodium bicarbonate/sodium pyruvate, liquid, sterile-filtered) (Invitrogen #11995-073), Dulbecco’s Modified Eagle’s Medium (DMEM), no phenol red (Invitrogen #21063-029), Dulbecco’s Modified Eagle Medium/Nutrient Mixture F-12 HEPES (DMEM/F-12) (Invitrogen #11330-057), Dulbecco’s Phosphate Buffered Saline (PBS) 1× (with MgCl and CaCl_2_) (Invitrogen #14040-182), Foetal bovine serum (FBS) (Invitrogen #26140-079), horse serum (HS) (Invitrogen #16050-122), trypsin-EDTA 10× (Invitrogen #15400-054), penicillin-streptomycin (P/S) solution (5,000 U/mL) (Invitrogen #15070-063), and page ruler prestained protein ladder (Invitrogen #26617) were purchased from ThermoFisher Scientific (Brisbane, Australia), unless otherwise stated. MEGM^TM^ Mammary Epithelial Cell Growth Medium SingleQuots^TM^ Kit (#CC-4136) was purchased from Lonza, Sydney, Australia. The deionized water (d.i.H_2_O) used for the experiments was obtained from a MilliQ^TM^ system (Millipore Australia Pty Ltd., Sydney, Australia). All chemicals and reagents were of spectroscopic grade and used without further purification. Methanol was purchased from Honeywell Research Chemicals, Morris Plains, USA. Ammonium bicarbonate, dithiothreitol (DTT), chloroform, RNase A, triton X-100, sodium chloride (NaCl), tris base, bovine serum albumin (BSA), sucrose, and *D*-phenylalanine were purchased from Sigma-Aldrich, Sydney, Australia. Cell proliferation reagent WST-1 was from Roche Diagnostics, Indianapolis, USA. 10× Tris-buffered saline (TBS10×),10× tris/glycine/SDS (TGS10×), 10× tris/glycine buffer (TG10×) and 4× laemmli sample buffer were purchased from Bio-Rad (Gladesville, Australia). Anti-Rabbit secondary antibody (horseradish peroxidase (HRP) Linked) (#7074S) and Anti-Mouse secondary antibody (HRP-linked) (#7076S) were purchased from Cell Signalling, Danvers, USA. All antibodies were purchased from Abcam (Cambridge, MA, USA) unless otherwise stated. All cell culture flasks and plates were purchased from Corning Inc. (Gilbert, AZ, USA) unless otherwise specified.

### 4.2. Cell Line and Complex Synthesis

Colorectal Cancer Cells (HT29) (ATCC, USA—HTB-38) and non-tumourigenic Immortalised Human Breast Epithelial Cells (MCF10A) (ATCC, Manassas, VA, USA—CRL-10317) were purchased from the American Type Culture Collection. HT29 cells were cultured in DMEM supplemented with 10% Foetal Bovine Serum (FBS) and 1% penicillin–streptomycin in a humidified cell incubator with 5% CO_2_ at 37 °C. MCF10A were cultured in DMEM/F12 supplemented with 5% Horse Serum, 1% penicillin–streptomycin, EGF (20 ng/mL), Hydrocortisone (0.5 mg/mL), Cholera Toxin (100 ng/mL), Insulin (10 µg/mL), Pen/Strep (100× solution,) (MEGM kit without gentamicin/amphotericin solution) and in a humidified cell incubator with 5% CO_2_ at 37 °C. Synthesis and characterisation of **Pt^IV^PCLB**, **Pt^IV^5CLB**, and **Pt^IV^56CLB** (Scheme 1) were afforded using published methods as reported in Supplementary Method S1 and Result S1 [24,172,173,174].

### 4.3. Cytotoxicity of Chlorambucil-Platinum(IV) Prodrugs

Cells (HT29 or MCF10A, 1000 cells/well) were cultured in 96-well flat bottom plates and incubated overnight. The platinum prodrugs, as well as their bioactive ligand and cisplatin were assessed for their individual cytotoxicity using 3-fold dilutions starting at 150 to 0.007 µM, as previously described [43]. Three separate experiments were implemented, and each sample was run in triplicates. The selective cytotoxicity index (SCI) for **Pt^IV^PCLB**, **Pt^IV^5CLB**, **Pt^IV^56CLB**, ligand, and cisplatin were calculated by dividing the IC_50_ (Table 1) values of the complexes or ligands in the non-tumourigenic immortalised human breast cell line MCF10A by their IC_50_ in the cancerous colorectal cell line HT29. The SCI identifies the therapeutic window between cytotoxicity and anticancer action [175,176]. The bigger the SCI the greater the selectivity and therapeutic activity towards cancer cells [24].

### 4.4. Cytotoxicity Evaluation of Chlorambucil-Platinum(IV) Prodrugs in a Cancer Spheroid Model

To evaluate the cytotoxic effect of platinum prodrugs, CLB, and cisplatin in 3D cell culture, cells were bioprinted with the non-contact drop-on-demand 3D RASTRUM bioprinter (Inventia Life Science, Alexandria, Australia) [71,177]. The 3D large plug cell model with polythene glycol bioink formulations was selected for this investigation (Inventia Life Science, #Px02.09 for HT29 spheroids, Table S1). After the automated priming of all fluids into the printer nozzles, 10,000 cells/well were printed into tissue culture plates (Nunc^TM^ MicroWell^TM^ 96-Well, Nunclon Delta-Treated, Flat-Bottom Microplate, ThermoFisher #167008), with RASTRUM creating the 3D Large Plug across the 96-well plate. Using multichannel pipettes, DMEM culture medium was added to each well. The plate was incubated for a full week as previously described [43]. After spheroids grew, the media was removed and 100 μL was replenished. The platinum complexes, as well as bioactive ligands and cisplatin individual cytotoxicity, were then assessed using 3-fold dilutions starting at 150 to 0.007 μM. The cytotoxicity was then evaluated using the WST-1 assay as previously described [43].

### 4.5. Cellular Uptake of Chlorambucil-Platinum(IV) Prodrugs

A final concentration of 10^6^ HT29 cells/well was seeded in 6-well plates and incubated overnight to adhere. The cells were then treated with a final concentration of 3 μM of **Pt^IV^PCLB**, **Pt^IV^5CLB**, or **Pt^IV^56CLB** for 0, 0.5, 1, 3, 6, 12, 24, or 30 h. The media was then removed, and cells were washed with cold PBS thrice. The following day, 400 μL of 69% HNO_3_ was added to each well for complete digestion for 1 h and 30 min. The digests were moved to trace-metal-free 15 mL centrifuge tubes (Labcon, Petaluma, CA, USA), to which 7 mL milliQ water was then added. A concentration of 50 ppb ^193^Ir in 2% HNO_3_ solution was used for the Perkin Elmer NexION^®^ Inductively Coupled Plasma Mass Spectrometer (ICP-MS). Mixing and injection of sample and internal standard solution was conducted using an ESI 7 port Valve system (Elemental Scientific, Omaha, NE, USA) as previously described [43]. The ICP-MS was tuned daily, and its performance optimised to a set of suppliers recommended settings. All machine parameters are described in Table S2. The results are averages of three different experiments run in triplicates (±SEM) and expressed as nmol/10^6^ cells or μM/cell. Quantification of the cellular uptake of Pt was based on external standards (Certified Standard from Sigma Merck, Sydney, Australia) (Figure S1 for calibration curve) containing Internal standard Ir. The calibration curve is shown in Figure S1.

### 4.6. Mode of Uptake of Chlorambucil-Platinum(IV) Prodrugs

A final concentration of 10^6^ HT29 cells/well was seeded in 6-well plates and incubated overnight to adhere. Cells were then treated with 3 μM of **Pt^IV^PCLB**, **Pt^IV^5CLB**, or **Pt^IV^56CLB** prodrugs under different conditions, to identify the mode of uptake as previously described [43]. The findings of exploratory tests were used to define the incubation times. To investigate the effect of temperature, prodrugs were added to cells in DMEM and incubated for 2 h at 4 °C or 37 °C. For 2 h, cells were pre-treated with 1 μg/mL of anti-transferrin antibody to inhibit transferrin receptor-mediated uptake. This was followed by a further 2 h of incubation at 37 °C with 3 μM of each individual prodrug in the presence of the antibody. The cells were pre-treated with 0.45 M of sucrose in serum-free medium for 30 min and then incubated with 3 μM of each prodrug for 2 h to block Clathrin-mediated endocytosis. To block the SLC7A5 transporter, cells were treated with 1 mM *D*-phenylalanine for 2 h, followed by 3 μM **Pt^IV^PCLB**, **Pt^IV^5CLB**, or **Pt^IV^56CLB** and incubated for 2 h. Following the procedure outlined in Section 4.5, the medium was eliminated, and the intracellular absorption of platinum was assessed using ICP-MS.

### 4.7. Cellular Localisation of Chlorambucil-Platinum(IV) Prodrugs

HT29 cells (10^6^ cells/well) were seeded in 6-well plates and incubated overnight. Cells were then treated with 3 μM of either **Pt^IV^PCLB**, **Pt^IV^5CLB**, or **Pt^IV^56CLB** for 24 h. Following the removal of the media, the cells were trypsinised after three ice-cold PBS washes. The isolation of individual fractions was carried out using the Fraction-PREP Cell Fractionation kit (Abcam, Cambridge, USA) as previously described [43]. After resuspension of the cell pellet in ice-cold PBS, it was transferred to Eppendorf tubes for a 5 min 700 × *g* spin cycle to extract the supernatant. After that, the pellet was suspended in 400 μL of cytosol extraction buffer mix and left to sit on ice for 20 min. The samples were centrifuged at 700 × *g* for 10 min and the supernatant was collected as the cytosolic fraction. The pellet was then resuspended in 400 μL of ice-cold membrane A extraction buffer mix and vortexed for 10 s. Then, membrane B extraction buffer mix was added and incubated on ice for 1 min. The sample was next vortexed and centrifuged for 5 min at 1000 × *g*. The supernatant having the membrane/particulate fraction was then transferred to chilled Eppendorf tubes. The pellet was then suspended in 200 μL of ice-cold nuclear extraction buffer, vortexed for 20 s and left to sit on ice for 40 min, while vortexing every 10 min. Next, samples were centrifuged for 10 min at maximum speed. The supernatant was collected into chilled Eppendorf tubes as it contained the nuclear fraction, while the pellet was the cytoskeletal fraction. The sample buffers of all fractions were then evaporated at 100 °C and then incubated with 180 μL of nitric acid for 1 h and 30 min. Milli-Q water was then added to a final volume of 3 mL. Platinum cell localisation was then measured via ICP-MS, as described in Section 4.5. All experiments were carried out in triplicate.

### 4.8. Cell Death Analysis

At 72 h after each investigated agent treatment, the cells were examined using Annexin V-fluorescein isothiocyante (Annexin V-FITC) and Propidium Iodide (PI) staining (Abcam, Cambridge, USA) as previously described [43]. In a 6-well plate, HT29 cells (2 × 10^5^ cells/well) were seeded and treated with the IC_50_ concentration (Table 1) and incubated for 72 h. The supernatant from each well was next transferred to labelled tubes. 300 μL of trypsin was then added per well. The separated cells were then transferred to designated tubes. The cells were then counted with trypan blue using an inverted microscope (Nikon Eclipse TS100, Sydney, Australia) and diluted to 500 cells/μL which was centrifuged at 500× *g* for 5 min at 4 °C. The pellet was immersed in 100 μL of 1× Annexin V Binding Buffer. Each sample was transferred to round-bottomed polystyrene tubes (Interpath Services, Somerton, Australia). The study involved adding 2 μL of each Annexin V-FITC and PI to each tube, incubating for 10 min, and analysing each cell using a benchtop flow cytometer (Biosciences, Erembodegem, Belgium). The Annexin V/PI data was processed using FlowJoTM v10.9 software, on a scatter plot of FL1-H vs. FL2-H. Three experiments were conducted in triplicate.

### 4.9. Cell Cycle Arrest

HT29 cells were plated at 2 × 10^5^ cells/well in a 6-well plate, treated with IC_30_ concentration (cisplatin (IC_30_ 73.95 ± 1.97 μM), CLB (IC_30_ 61.94 ± 1.87 μM), **Pt^IV^PCLB** (IC_30_ 0.42 ± 1.61 μM), **Pt^IV^5CLB** (IC_30_ 0.30 ± 1.22 μM), **Pt^IV^56CLB** (IC_30_ 0.15 ± 1.62 μM) at 72 h, a dose that was established for each complex’s treatment. At 72 h post treatment, the supernatant from each well was transferred into correspondingly labelled tubes. A total of 300 μL of trypsin was then added per well. The separated cells were then transferred to designated tubes. Following two PBS washes, the cells were resuspended in 70% ethanol and kept at 4 °C overnight. Following another two PBS washes, the fixed cells were stained for 45 min with PI diluted to 50 μg/mL in 10 mM Tris-Cl, pH 8.0, 10 mM NaCl, 0.1% Triton X-100, and 100 μg/mL RNase A. After that, the BD FACSCanto II Benchtop Flow Cytometer (Biosciences, Erembodegem, Belgium) was used to evaluate the cell-cycle profile. FlowJo^TM^ v10.9 was used to analyse the data. Three experiments were conducted in triplicate as previously described [43].

### 4.10. Reactive Oxygen Species (ROS) Detection Assay

A DCFDA/H2DCFDA-cellular ROS Assay Kit (Abcam, Cambridge, USA) was utilised, as previously described [24,43,133,178], to examine the production of ROS in treated cells. In 96-well plates, 2500 HT29 cells/well were cultured and incubated overnight. The cells were washed with 1× kit buffer, followed by a 45 min incubation with 25 μM 2′,7′-dichlorofluorescein diacetate (DCFH-DA). Following the removal of DCFH-DA, the cells were washed with 1× kit buffer before phenol red-free medium was added. Cells were next treated using IC_50_ concentration (Table 1) from 72 h for each prodrug, CLB, and cisplatin. At an excitation/emission of 485/535 nm, the plates were scanned using the Glo-Max^®^-Multimode microplate scanner (Promega Corporation, Alexandra, Australia) to measure fluorescence in relative fluorescence units (RFU) at various time points. To produce the positive control (20 μM tert-butyl hydroperoxide; TBHP), cells were first stained with DCFDA as described above then TBHP was added in phenol red-free medium, and the mixture was then scanned. Three s experiments were implemented, and each sample was run in triplicates.

### 4.11. Mitochondrial Membrane Potential

A TMRE-MtMP Assay Kit (Abcam, Cambridge, USA) was used to study the MtMP changes, as previously described [38,43,178]. The 96-well plates were seeded with 2500 HT29 cells/well. Cells were treated with IC_50_ drug concentration (Table 1) for each prodrug, CLB and cisplatin. Cells were stained with 1 μM tetramethylrhodamine, ethyl ester (TMRE) for 30 min after being rinsed with PBS at 24, 48, or 72 h. After TMRE was removed, cells were washed with PBS containing 0.2% BSA thrice before phenol red-free DMEM was added. The Glo-Max^®^-Multimode microplate scanner (Promega Corporation, Alexandra, Australia) was then used to scan the plates at an excitation/emission of 549/575 nm to measure the fluorescence expressed as RFU. To produce the positive control, 20 μM carbonyl cyanide 4-(trifluoromethoxy) phenylhydrazone (FCCP)) was added to the cells and incubated for 10 min then stained with TMRE as described above. Three independent experiments were implemented, and each sample was run in triplicates.

### 4.12. Immunofluorescence Morphological Changes in Microtubule Organisation Using Confocal Microscopy

Millicell 8-well chamber slides (Merck, Darmstadt, Germany) were seeded with 1000 HT29 cells/well in order to examine the changes in actin and tubulin expression and morphology in treated cells as previously described [43]. Cells were treated with IC_50_ concentration (Table 1) of each investigated agent for 72 h. Cells were then washed with PB (75 mM disodium phosphate, 25 mM monosodium phosphate, pH. 7.4) for 5 min. Samples were then fixed with 4% paraformaldehyde (200 μL) for 10 min and permeabilized with 200 μL of 0.2% Triton X-100 in PBS for 10 min. Samples were then blocked with 1% Bovine Serum Albumin (Sigma-Aldrich) in PB for 30 min and then washed with 1× PBS and two washes with 0.1% PBS-Tween before incubation with primary conjugated antibodies for β-tubulin antibody (Rabbit mAb (9F3) Alexa Fluor(R) 488 Conjugate, Cell Signalling, Danvers, MA, USA) and phalloidin antibody (iFluor 555 reagent, ab176756, Abcam, Cambridge, USA) for 1 h each at room temperature. The cells were then washed with 0.1% PBS-Tween twice. The chamber was removed from the slide, and cells were mounted with ProLong^™^ Gold Antifade Mountant with DAPI (ThermoFisher, Eugene, OR, USA). Coverslips were sealed to the slide and allowed to air-dry. Samples were imaged with the Zeiss LSM 800 confocal microscope. For fluorescence intensity measurements, imaging parameters were constant for all conditions and were taken at a 20× magnification. Actin and tubulin expression were analysed using CellProfiler^™^ software 4.2.5. For morphological changes, images were taken at a 63× magnification. *n* = 30 cells were analysed. Images were acquired with an Airyscan detector and processed using the Zen Blue Airyscan processing module.

### 4.13. Wound Healing Assay

HT29 cells were seeded at a density of 10^5^ cells/well in an Incucyte^®^ Imagelock 96-well plate (Sartorius, Gottingen, Germany) and incubated overnight. Wounds were simultaneously made using the Incucyte^®^ 96-Well ESSEN Bioscience woundmaker tool (Sartorius, Gottingen, Germany) as previously explained [43,179,180,181,182,183]. Following wound formation, the media was removed, and cells were washed with PBS. DMEM was added and cells were treated with IC_50_ drug concentration (Table 1) of each complex for 72 h. The plate was then incubated in the Incucyte^®^ Live-Cell analysis system for scanning for 72 h (objective: 10×, channel selection: phase contrast, scan type: scratch wound, scan interval: every 4 h). Then, data was processed and interpreted using the Incucyte^®^ Scratch Wound Analysis Software Module (Cat. No. 9600-0012). Three separate experiments were implemented, and each sample was run in triplicates.

### 4.14. Western Blot Analysis

HT29 cells were seeded in 6-well plates 2 × 10^5^ cells/well and incubated with IC_50_ concentration (Table 1) of the investigated agents for 72 h. Supernatants were collected and centrifuged at 500× *g* for 5 min at 4 °C. Meanwhile, 200 μL of cell lysis RIPA buffer was used for protein extraction and protein content was measured using the Bio-Rad protein assay (Bio-Rad, Hercules, CA, USA) as previously described [43]. The proteins were subject to sodium dodecyl-sulfate polyacrylamide gel electrophoresis (SDS-PAGE) using Bolt 4–12% Bis-Tris gel (ThermoFisher, Eugene, USA) at 80 V for 30 min and then 120 V for 60 min with PageRuler™ Prestained Protein Ladder or SeeBlue Prestained Protein Standard (Invitrogen #LC5625) (ThermoFisher, Eugene, USA). Proteins were transferred using the semi-dry blot transfer on a 0.2 μm PVDF membrane (Cytiva, Amersham, UK) for 30 min. The membrane was blocked in 5% BSA in TBST, for 1 h and probed overnight with primary antibodies against apoptotic, anti-apoptotic, proliferative and autophagic protein markers (Table S6) at 4 °C as per manufacturers protocol listed in Table S1. After removing the primary antibody, the membranes were washed in TBST thrice for 10 min. Then, for 1 h and 30 min, membranes were probed with either mouse or rabbit HRP-coupled secondary antibodies (1/5000). Chemiluminescence ECL kit (ThermoFisher, Eugene, USA), was used for protein band detection. Blot images were obtained using the Odyssey^®^ FC imaging apparatus (LI-COR Biosciences, Lincoln, NE, USA) and band intensities were normalised to GAPDH and then normalised each replicate gel to its corresponding no-treatment control using the ImageJ v1.53t software (National Institutes of Health, Bethesda, MD, USA).

### 4.15. Proteomics

A total of 2 × 10^5^ HT29 cells/well were seeded in 6-well plates and incubated with IC_50_ concentration (Table 1) of the platinum complexes for 72 h for proteomic analysis as previously described [43]. The supernatant was collected, and cells detached using trypsin. Cells were then centrifuged at 500 × *g* for 5 min at 4 °C. Supernatant was discarded and PBS was used to wash the cell pellet, which was then resuspended in 300 µL RIPA for 30 min on ice. The cellular extract was next centrifuged at 12,000× *g* for 15 min at 4 °C, and the proteins (supernatant) were collected into Lo-Bind Eppendorfs. Protein content was assessed using Bio-Rad protein assay. The protein extraction and delipidation process involved adding 450 µL of methanol to 100 µL of cell homogenate and vortexed, followed by adding 150 µL of chloroform. Then 450 µL water was added, vortexed and centrifuged for 5 min at 12,000 × *g*, which formed the protein pellet at the organic/aqueous interface. After removing the chloroform layer, 400 µL of methanol was added and vortexed. The sample was centrifuged at 12,000 × *g* for 15 min and the supernatant was then aspirated and discarded. The pellet was washed with methanol (400 µL). The supernatant was removed, and the pellets were air-dried overnight. Next reduction, alkylation, and tryptic digestion of the protein were performed. An amount of 0.1% RapiGest (Waters, Milford, CT, USA) in 50 mM aqueous ammonium bicarbonate (20 µL) was added to the pellet and vortexed for effective suspension to dissolve the pellet. Then, a final concentration of 5 mM DTT in 50 mM aqueous ammonium bicarbonate was added. Samples were then heated for 30 min at 60 °C and cooled to room temperature. Next, a final concentration of 15 mM iodoacetamide in 50 mM aqueous ammonium bicarbonate was added. The mixture was left to react in the dark for 30 min. Trypsin Gold, Mass spectrometry grade (Promega, Sydney, Australia) (10 ng/µL) was then added for digestion at room temperature overnight. The following day, 50 µL of 4% (*v/v*) aqueous trifluoroacetic acid was added to each tube. Samples were heated for 45 min at 37 °C for effective decomposition of RapiGest. Samples were centrifuged at 12,000 × *g* for 10 min at 4 °C and supernatants were transferred to total recovery vials and run on a nanoAcquity Ultra Performance Liquid Chromatography (UPLC) coupled with a Synapt G2-Si instrument (Waters, Milford, USA) for label-free quantitative protein profiling. Nano-proteomics method is detailed in Method S2. Three independent experiments in triplicates were implemented, while all samples were analysed in a single run with a single injection (1 µL) for each trial. Mass spectrometry (MS) data was processed using Progenesis QI for proteomics (version 4.1, Nonlinear Dynamics, Newcastle upon Tyne, UK), as previously described [43].

### 4.16. Statistical Analysis

Data were presented as Mean ± SEM from three independent trials. Statistical analysis used for each data set is noted in Figure legends. Group differences were considered statistically significant if *p* < 0.05. * *p* < 0.05, ** *p* < 0.01, *** *p* < 0.001 and **** *p* < 0.0001 in comparison to the control group, unless otherwise stated.

## 5. Conclusions

HT29 cancer cells and spheroids were effectively treated with the chlorambucil-platinum(IV) prodrugs. The principal mechanism of cellular absorption was active transport, whereby **Pt^IV^56CLB** was predominantly accumulated intracellularly and all complexes were principally localised in the cytoskeleton. The prodrugs’ mechanisms of action caused considerable reduction in MtMP and formation of ROS. Additionally, alterations to the microtubule cytoskeleton were evident. In addition, the prodrugs caused apoptosis by inhibiting the cell proliferation pathways and causing an active interaction between the extrinsic and intrinsic routes. Additionally, there may be a role for autophagy-dependent cell death. Targeting important pathways of a disease and reducing resistance are important goals of multi-mechanistic drugs. According to the current research, the **Pt^IV^5CLB** and **Pt^IV^56CLB** prodrugs exhibit qualities of an effective and perhaps safer anti-tumour medication for colorectal cancers. Subsequent in vivo investigations are required to verify the safety, effectiveness, and selectivity of the chlorambucil-platinum(IV) prodrugs under investigation.

## 6. Patents

This work is part of Australian Provisional Patent Application 2022900110, Platinum(IV) complexes, February 2022, Western Sydney University, Sydney, Australia.

## Figures and Tables

**Table 1:** IC_50_ values of CLB, **Pt^IV^PCLB**, **Pt^IV^5CLB**, or **Pt^IV^56CLB** prodrugs, and cisplatin on HT29, HT29 spheroids, and MCF10A cell lines. The data for cisplatin were obtained from previously reported data run in parallel [43].

IC_50_ (µM)	Cisplatin	CLB	Pt^IV^PCLB	Pt^IV^5CLB	Pt^IV^56CLB
MCF10A	4.71 ± 1.47 ^a^	120.70 ±1.95 ****^b^	5.57 ±1.48	2.82 ± 1.29	0.97 ±1.34
HT29	77.76± 1.97	117.10 ± 1.87 ***	0.68 ± 1.61 ****	0.41 ± 1.22 ****	0.28 ± 1.62 ****
HT29 Spheroids	444.80± 5.09	172.1 ± 2.07 ****	3.29 ±1.35 ****	2.27 ± 1.29 ****	0.70 ± 1.34 ****

^a^ Data are mean ± SEM of three independent determinations. ^b^ Significance of IC_50_s compared to cisplatin are identified by *** *p* < 0.001 and **** *p* < 0.0001, as measured by an unpaired Student’s *t*-test.

**Table 2:** Selective cytotoxicity index of CLB, **Pt^IV^PCLB**, **Pt^IV^5CLB**, or **Pt^IV^56CLB** prodrugs, and cisplatin in cancer cells.

* SCI	Cisplatin	CLB	Pt^IV^PCLB	Pt^IV^5CLB	Pt^IV^56CLB
HT29	0.06	1.03	8.19	6.88	3.46

* SCI = IC_50_ (normal epithelial cell line, MCF10A)/IC_50_ (cancer cell line).

## Data Availability

All data relevant to the publication are included.

## Supplementary Materials

The following supporting information can be downloaded at: https://www.mdpi.com/article/10.3390/ijms25158252/s1.
