# Supplementary material for: Chemotherapeutic Potential of Chlorambucil-Platinum(IV) Prodrugs against Cisplatin-Resistant Colorectal Cancer Cells
Source: Int J Mol Sci. 2024 Jul 28;25(15):8252. doi: 10.3390/ijms25158252 (PMC11312340; doi:10.3390/ijms25158252)
Supplement: Supplementary file 1 [file ijms-25-08252-s001.zip › ijms-3078877-supplementary.pdf]

Supplementary

Chemotherapeutic Potential of Chlorambucil-Platinum(IV) Prodrugs Against Cisplatin-Resistant Colorectal Cancer Cells

Maria George Elias [1,2], Angelico D. Aputen [1], Shadma Fatima [2,3], Timothy J. Mann [2,3], Shawan Karan [1], Meena Mikhael [4], Paul de Souza [5], Christopher P. Gordon [1], Kieran F. Scott [2,3] and Janice R. Aldrich-Wright [1,2,*]

[1] School of Science, Western Sydney University, Sydney, NSW 2751, Australia; m.elias3@westernsydney.edu.au (M.G.E.); a.aputen@westernsydney.edu.au (A.D.A.); shawan.karan@westernsydney.edu.au (S.K.); c.gordon@westernsydney.edu.au (C.P.G.)

[2] Medical Oncology, Ingham Institute for Applied Medical Research, Liverpool Hospital, Liverpool, NSW 2170, Australia; s.fatima@westernsydney.edu.au (S.F.); tim.mann@unsw.edu.au (T.J.M.); kieran.scott@westernsydney.edu.au (K.F.S.)

[3] School of Medicine, Western Sydney University, Sydney, NSW 2751, Australia

[4] Mass Spectrometry Facility, Western Sydney University, Sydney, NSW 2751, Australia; m.mikhael@westernsydney.edu.au (M.M.)

[5] Nepean Clinical School, Faculty of Medicine and Health, University of Sydney, Kingswood, NSW 2747, Australia; paul.desouza@sydney.edu.au

* Correspondence: j.aldrich-wright@westernsydney.edu.au; Tel.: +61-2-4620-3218

Method S1. Syntheses of Chlorambucil-platinum(IV) prodrugs

The general syntheses of Chlorambucil anhydride

The anhydride of Chlorambucil (CLB) was synthesised according to literature [1,2]. CLB (400 mg; 1.20 mmol) and 1 mol eq. of *N*,*N*-dicyclohexylcarbodiimide (DCC; 275 mg; 1.20 mmol) were dissolved in acetone (30 mL) for 72 h at room temperature (RT). Then, vacuum filtration was used to remove the by-product dicyclohexylurea (DCU) leaving a colourless filtrate that was reduced under vacuum using a rotary evaporator, which formed a sticky precipitate. The precipitate was diluted with ethyl acetate (EtOAc; 20 mL) and settled for 48 h at 4 ºC to remove any excess DCU. The resulting solution was filtered with a syringe and reduced under vacuum into a thick transparent oil.

The general syntheses of [Pt(P_L)(1*S*,2*S*-diaminocyclohexane)Chlorambucil(OH)](NO_3)$_2$

[Pt(P_L)(1*S*,2*S*-diaminocyclohexane)$(OH)_2$](NO_3)$_2$ was coordinated to CLB, a bioactive ligand (B_x), by substitution of either one of the hydroxido axial ligands to obtain [Pt(P_L)(1*S*,2*S*-diaminocyclohexane)B_x(OH)](NO_3)$_2$ [2].

Firstly, [Pt(P_L)(1*S*,2*S*-diaminocyclohexane)$(OH)_2$](NO_3)$_2$ was reacted with 2 – 5 mol eq. of anhydride of CLB in DMSO (3 – 4 mL) for 96 h at room temperature in the dark. Subsequently, excess diethyl ether (Et_2O) was added to the reaction solution and mixed. The solution was centrifuged to isolate a brown oil, which was then collected and diluted with minimal MeOH, followed by the addition of excess Et_2O to induce the precipitation of raw [Pt(P_L)(1*S*,2*S*-diaminocyclohexane)(CLB)(OH)](NO_3)$_2$. To isolate the precipitate, the solution was centrifuged at 2500xg. To remove excess CLB acid and CLB anhydride, excess acetone was mixed with the precipitate and sonicated, resulting in an almost pure and solidified precipitate (dark red-orange) that was obtained after centrifuging at 2500xg. A Biotage Isolera™ One flash chromatography system equipped with a Biotage® Sfär C18 D (Duo 100 Å 30 µm 30 g) was utilised to purify the CLB-platinum(IV) prodrugs. The mobile phase consisted of solvents, A (d.i.H_2O) and B (MeOH). The samples were dissolved in d.i.H_2O /MeOH (50:50) and eluted through the column with a 0–30% linear gradient for 50 min with a flow rate of 4 mL/min, collected within the set wavelengths of 200–400 nm. The successful syntheses and purity of the CLB-platinum(IV) prodrugs (**Pt^{IV}PCLB**, **Pt^{IV}5CLB** and **Pt^{IV}56CLB**; Scheme 1) was confirmed by high performance liquid chromatography (HPLC), ^{1}H nuclear magnetic resonance (^{1}H NMR), ^{1}H-^{195}Pt heteronuclear multiple quantum coherence (^{1}H-^{195}Pt-HMQC) NMR and circular dichroism (CD) spectroscopy [2-4].

HPLC chromatograms were determined by eluting the CLB-platinum(IV) prodrugs through an Agilent Technologies Infinity HPLC machine, equipped with a Phenomenx Onyx™ Monolithic C_{18}-reverse phase column (100 × 4.6 mm, 5 µm pore size). For organic compounds an Agilent ZORBAX RX-C_{18} column (100 × 4.6 mm, 3.5 µm pore size) was used. The mobile phase consisted of solvents, A (0.06% trifluoroacetic acid (TFA) in deionised water (d.i.H_2O)) and B (0.06% TFA in ACN/d.i.H_2O (90:10)). An injection volume of 5 µL was utilised and eluted with a 0 – 100% linear gradient over 15 min with a flow rate of 1 mL/min, within the set wavelengths of 214 and 254 nm. ^{1}H NMR was determined for each prodrug by dissolving the prodrug (5 mg) in deuterium oxide (D_2O) (500 µL). ^{1}H NMR was carried out on a 400 MHz Bruker Avance Spectrometer at 298 K. All samples were prepared to a concentration of 10 – 20 mM in 450 – 600 µL using D_2O. ^{1}H NMR was set to 10 ppm and 16 scans with a spectral width of 8250 Hz and 65536 data points. ^{1}H-^{195}Pt HMQC was carried out using a spectral width of 214436 Hz and 256 data points for ^{195}Pt nucleus, F1 dimension, also a spectral width of 4808 Hz with 2048 data points for ^{1}H nucleus, F2 dimension. All resonance recorded were presented as chemical shifts in parts per million (δ ppm). A Jasco J-810 CD spectrophotometer was used

to measure the CD spectra of the purified platinum(IV) prodrugs. The samples were prepared in d.i.H_2O, with a concentration of 0.05 mM and a 1 mm optical glass cuvette was used. CD experiments were performed in the range of 350–200 nm (20 accumulations) with a bandwidth of 1 nm, data pitch of 0.5 nm, a response time of 1 sec, and a 100 nm/min scan speed. The flowrate of nitrogen gas was 8 L/min. Excel was used to process the spectral data.

Result S2. Characterisation and yields of Chlorambucil anhydride and Chlorambucil-platinum(IV) prodrugs

HPLC, NMR (^{1}H and ^{1}H-^{195}Pt-HMQC) and CD were utilised to confirm the structures and purity of the CLB-platinum(IV) prodrugs for biological testing, passing for greater than 90% purity (Table H.S1). The obtained yields of the CLB-platinum(IV) prodrugs are reported in Table H.S1 and HPLC chromatograms, which include the retention time (t_R) of the prodrugs are also reported in Figures H.S2-S4. The obtained yield of the CLB anhydride was 587 mg, HPLC t_R: 17.044 min (Figure H.S1) [2]. The obtained ^{1}H and ^{1}H-^{195}Pt-HMQC NMR are tabulated in Table N.S1 and the spectra are reported in Figures N.S1–S7. The HPLC chromatograms (Figure H.S1-S4) and NMR spectra (Figure N.S1-S7) matched those of literature data, which confirmed the successful syntheses and isolation of our products of interest[2]. The CD experiments confirmed the retention of chirality of the 1*S*,2*S*-diaminocyclohexane. The obtained results for the CLB-platinum(IV) prodrugs were comparable to previously reported data [2]. All the characteristic peaks from CD spectra of the purified complexes are summarised in Table H.S1.

Table H.S1. Summary of the characterisation data of CLB anhydride and the CLB-platinum(IV) prodrugs.

Compounds and Complexes	Yields [mg]	Yields [%]	t_R [min]	Purity [%]	CD λ_{max} [nm] (mdeg.mol / L × 10^1)
CLB anhydride	587	83	17.044	NA[a]	–
**Pt^{IV}PCLB**	210	93	9.131	95.12	206 (–404), 258 (–2.03), 277 (–68.5)
**Pt^{IV}5CLB**	217	87	9.245	97.43	208 (–233.41), 232 (–7.77), 260 (+60.90), 286 (+2.41)
**Pt^{IV}56CLB**	130	81	9.552	90.84	212 (–239.13), 241 (–48.29), 260 (+64.91), 291 (–18.59)

[a] NA: not applicable. CLB anhydride was not purified as explained in Section 2.1.5.

Table N.S1. Summary of ^{1}H and ^{1}H-^{195}Pt-HMQC data of CLB anhydride and CLB-platinum(IV) prodrugs, showing the chemical shifts (ppm), integration, multiplicity and *J*-coupling constants. [a]

**Proton Labels**	**Compounds and Complexes**			
	CLB anhdyride	**Pt^{IV}PCLB**	**Pt^{IV}5CLB**	**Pt^{IV}56CLB**
H2/H9[b]	–	9.4 (d, 1H, J = 5.5 Hz)	9.3 (qd, 2H)	9.2 (d, 1H, J = 5.4 Hz)
H4/H7	–	8.9 (dd, 2H, J_1 = 8.3Hz, J_2 = 3.3 Hz)	H4: 9.1 (dd, 1H, J_1 = 8.4 Hz, J_2 = 4.3 Hz); H7: 8.9 (dd, 1H, J_1 = 8.3 Hz, J_2 = 3.6 Hz)	9.1 (dd, 2H, J_1 = 8.6 Hz, J_2 = 5.0 Hz)
H5/H6	–	8.3 (s, 2H)	7.9 (d, H6, 1H, J = 4.5 Hz)	–
H3/H8	–	8.4 (m, 2H)	8.2 (m, 2H)	8.2 (q, 2H, J = 5.5 Hz)
a and b, c and d	6.37 (q, J = 8.8 Hz)	6.4 (q, 4H, J = 8.8 Hz) (resonance overlapped)	6.3 (q, 4H, J = 8.3 Hz) (small distance between a and b and c and d resonances)	6.2 (q, 4H, J = 8.6 Hz) (greater distance between a and b and c and d resonances)
e, f, g and h	3.51 (m, 8H)	3.6 (m, 8H) (sharp resonance)	3.5 (s, 8H)	3.5 (s, 8H)
CH_3	–	–	2.72 (s, CH_3, 3H)	2.63 (d, 6H, J = 3.6 Hz)
H1'/H2'	–	3.2 (m, 2H)	3.2 (m, 2H)	3.2 (m, 2H)
H3'/H6' equatorial	–	2.5 (d, 2H)	2.4 (d, 2H)	2.4 (d, 2H)
α		1.9 (t, 2H)	1.9 (t, 2H)	1.9 (t, 2H)
β		Overlapping with H4'/H5' axial	Overlapping with H4'/H5' axial	Overlapping with H4'/H5' axial
γ		Overlapping with H4'/H5' equatorial and H3'/H6' axial	1.46 (m, 2H)	Overlapping with H4'/H5' axial
H4'/H5' equatorial and H3'/H6' axial	–	Overlapping with γ	1.68 (m, 4H)	–
H4'/H5' axial	–	Overlapping with β	Overlapping with β	–
H4'/H5' equatorial and H3'/H6' axial	–	–	1.68 (m, 4H)	1.69 (m, 4H)
H4'/H5' equatorial and H3'/H6' axial; γ	–	1.7 (m, 6H)	–	–
H4'/H5' axial; β	–	1.3 (m, 4H).	1.25 (m, 4H).	–
H4'/H5' axial; β; γ		–	–	1.26 (m, 6H)

$^{1}H/^{195}Pt$	–	9.30/540, 8.24/540	9.26/542	9.25/530, 9.22/530, 8.23/530

[a] Experiments were performed in DMSO-d_6 or D_2O. Accordingly, ammine or hydroxido resonances are not observed due to proton exchange. [b] Symmetrical complexes, thus H2/9, H3/8, H4/7 and H5/6 are equivalent protons.

HPLC Chromatograms

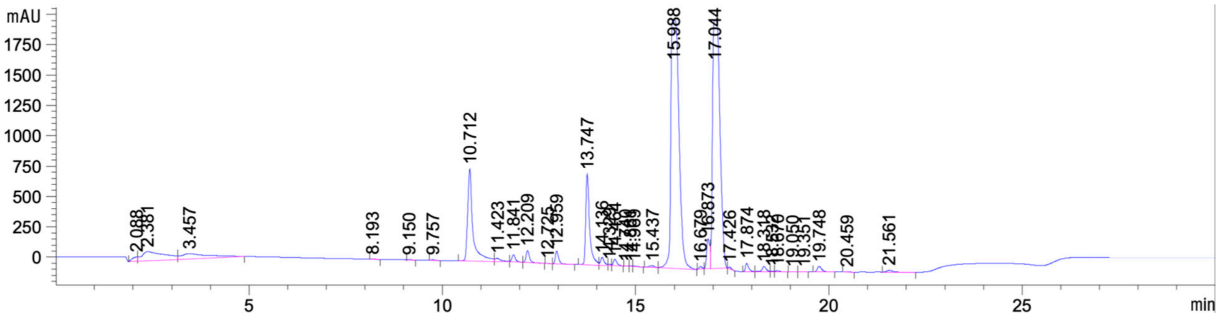

Figure H.S1. The HPLC chromatogram of raw CLB anhydride within the collection wavelength of 254 nm acquired at 298 K by an Agilent ZORBAX RX-C18 column (100 × 4.6 mm, 3.5 μm pore size). t_R at 17.044 min.

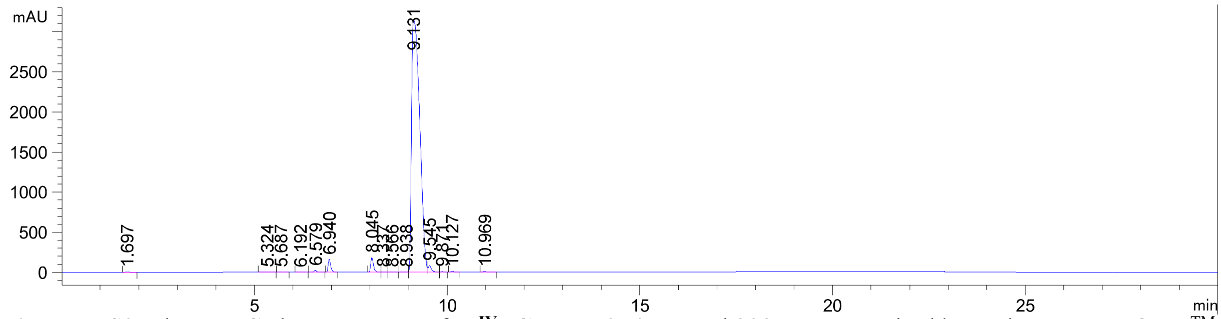

Figure H.S2. The HPLC chromatogram of **Pt^{IV}PCLB**, at 254 nm and 298K was acquired by a Phenomenex Onyx^{TM} Monolithic C18-reverse phase column (100 × 4.6 mm, 5 μm pore size). t_R at 9.131.

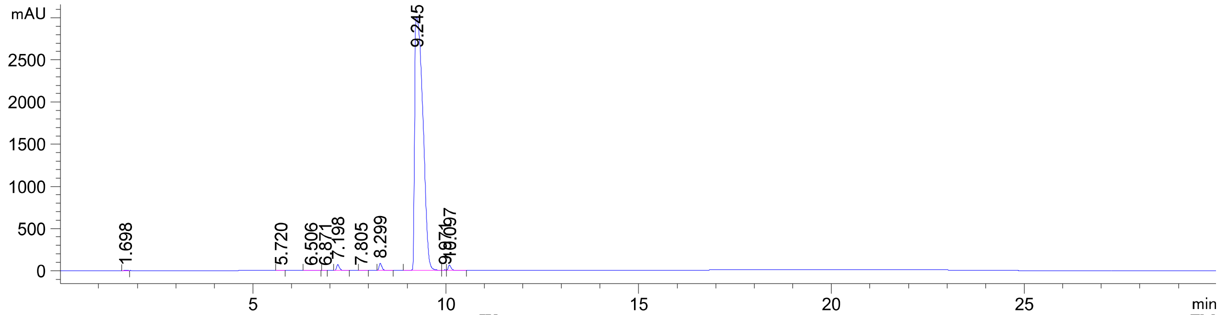

Figure H.S3. The HPLC chromatogram of **Pt^{IV}5CLB**, at 254 nm and 298K was acquired by a Phenomenex Onyx^{TM} Monolithic C18-reverse phase column (100 × 4.6 mm, 5 μm pore size). t_R at 9.245.

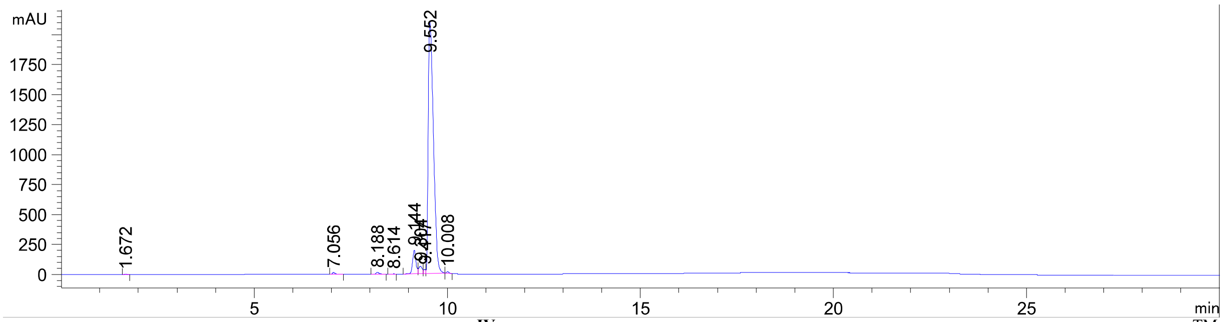

Figure H.S4. The HPLC chromatogram of **Pt^{IV}56CLB**, at 254 nm and 298K was acquired by a Phenomenex Onyx^{TM} Monolithic C18-reverse phase column (100 × 4.6 mm, 5 μm pore size). t_R at 9.552.

^{1}H NMR and ^{1}H-^{195}Pt HMQC Spectra

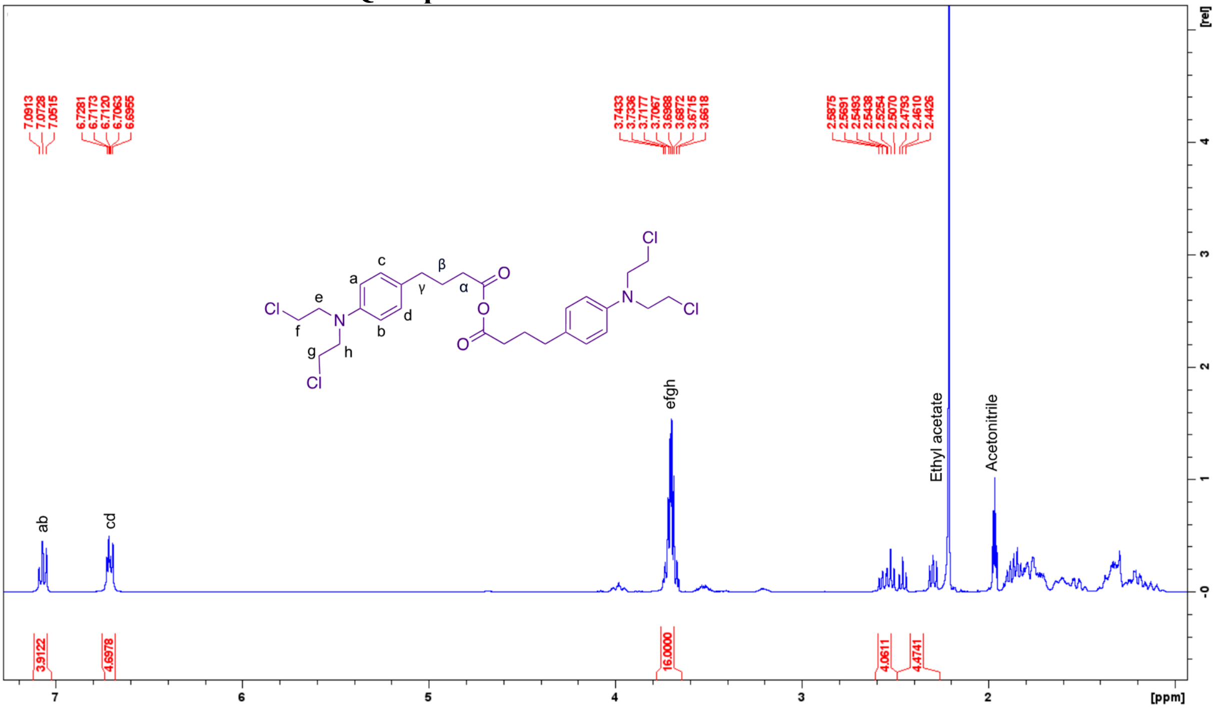

Figure N.S1. ^{1}H NMR spectra of CLB anhydride in DMSO-d6 obtained at 298 K. Inset: structure of CLB anhydride, with proton labelling system.

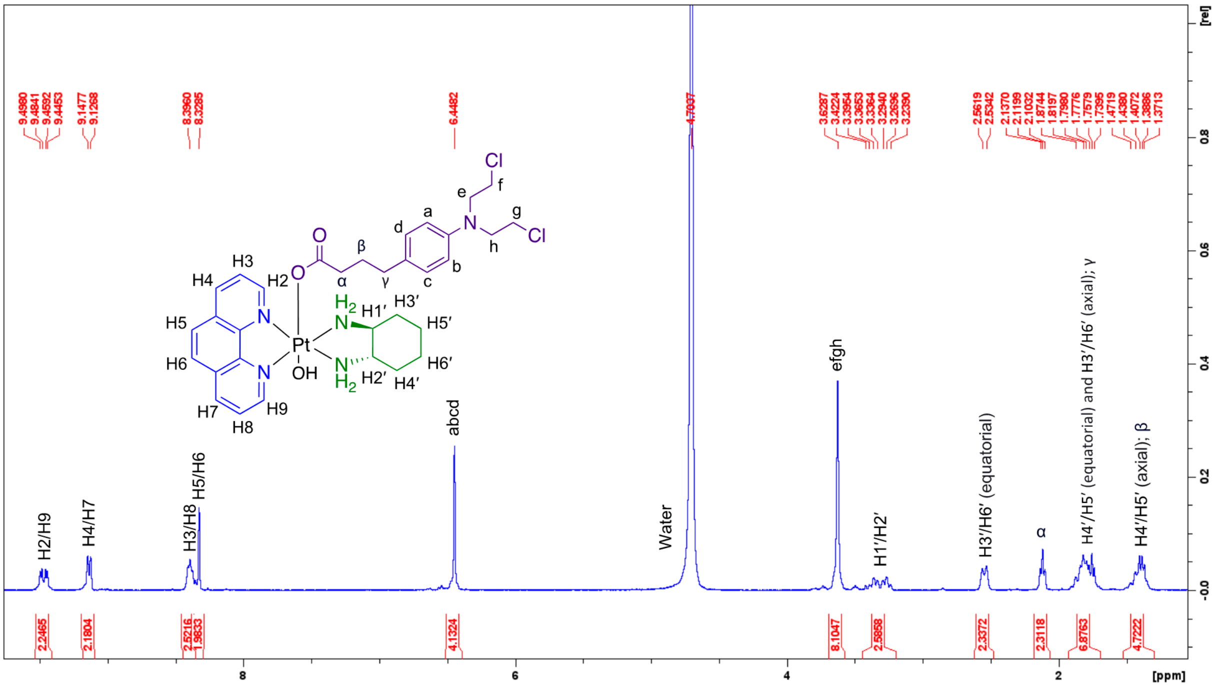

Figure N.S2. ^{1}H NMR spectra of **Pt^{IV}PCLB** in D_2O obtained at 298 K. Inset: structure of **Pt^{IV}PCLB**, with proton labelling system.

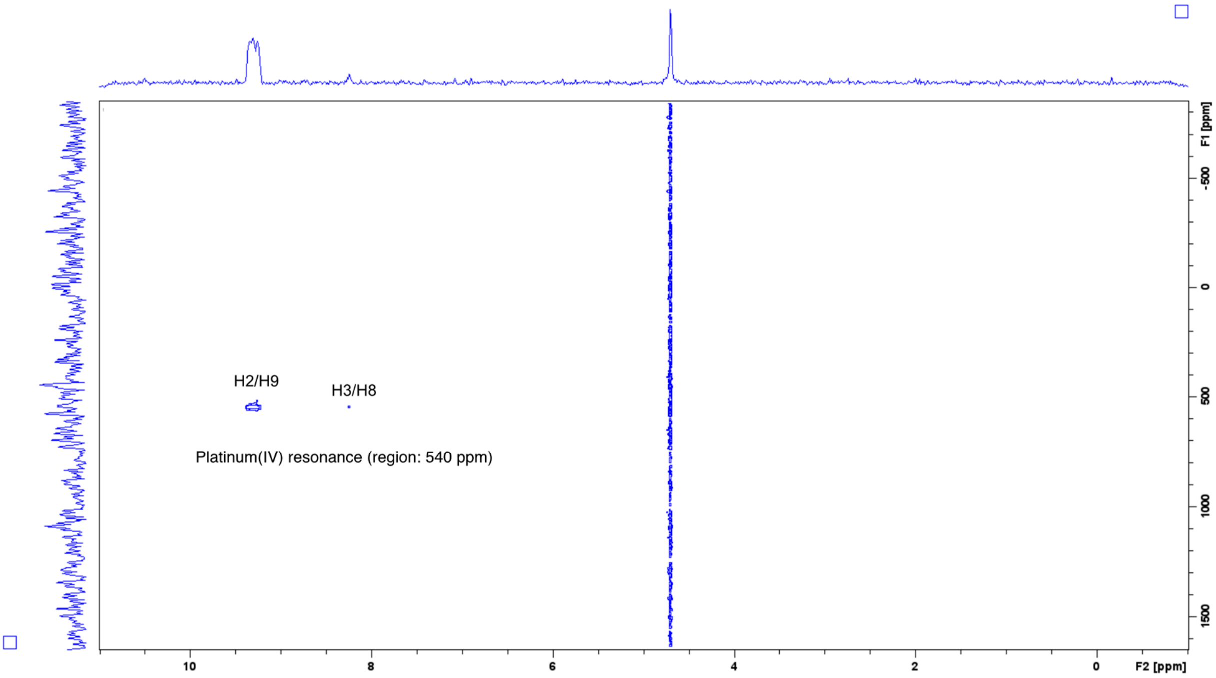

Figure N.S3. ^{1}H-^{195}Pt HMQC spectra of **Pt^{IV}PCLB** in D_2O obtained at 298 K.

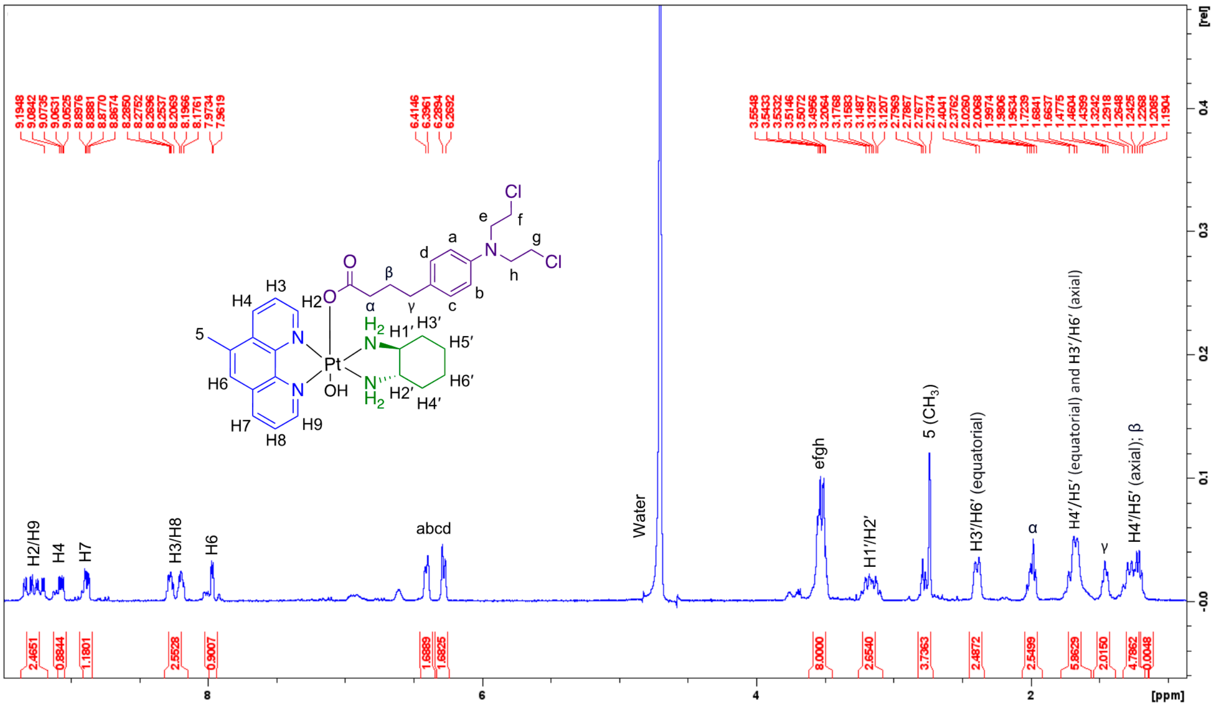

Figure N.S4. ^{1}H NMR spectra of **Pt^{IV}5CLB** in D_2O obtained at 298 K. Inset: structure of **Pt^{IV}5CLB**, with proton labelling system.

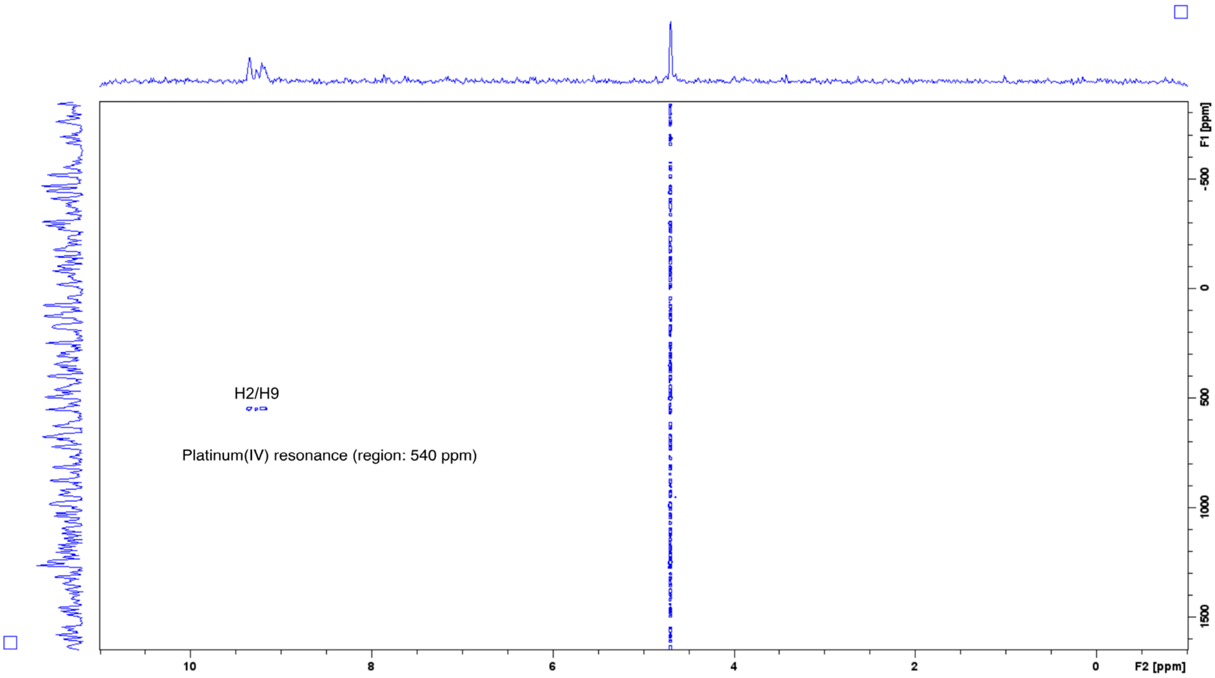

Figure N.S5. ^{1}H-^{195}Pt HMQC spectra of **Pt^{IV}5CLB** in D_2O obtained at 298 K.

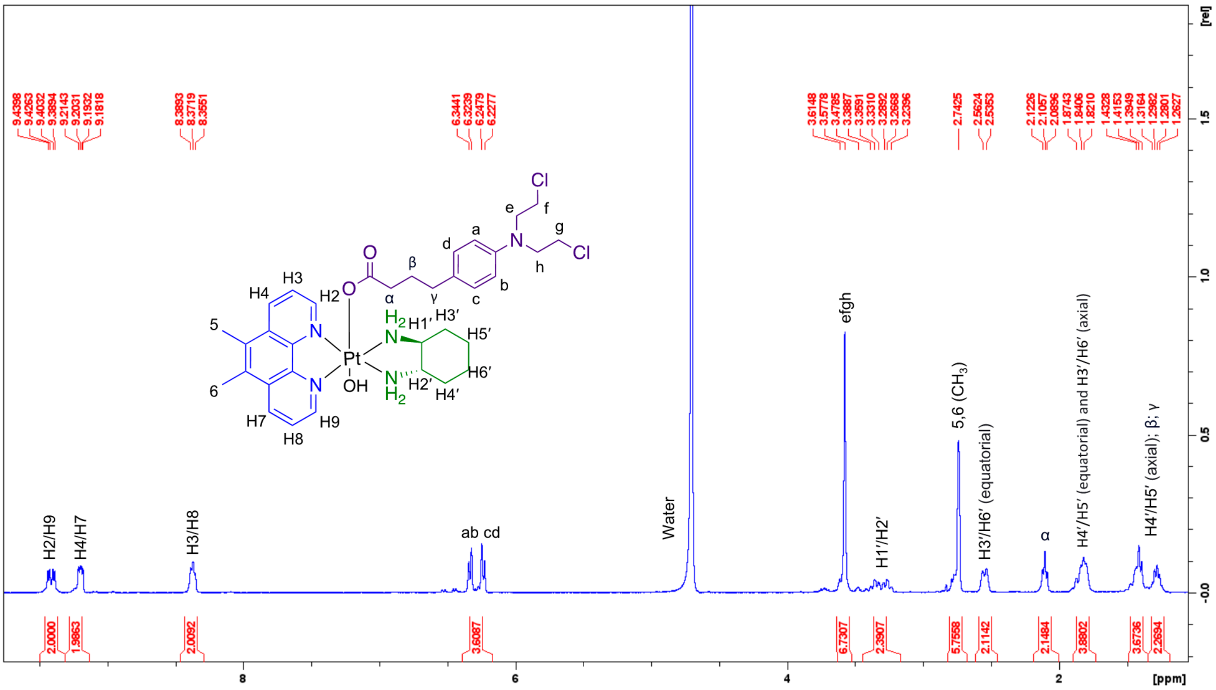

Figure N.S6. ^{1}H NMR spectra of **Pt^{IV}56CLB** in D_2O obtained at 298 K. Inset: structure of **Pt^{IV}56CLB**, with proton labelling system.

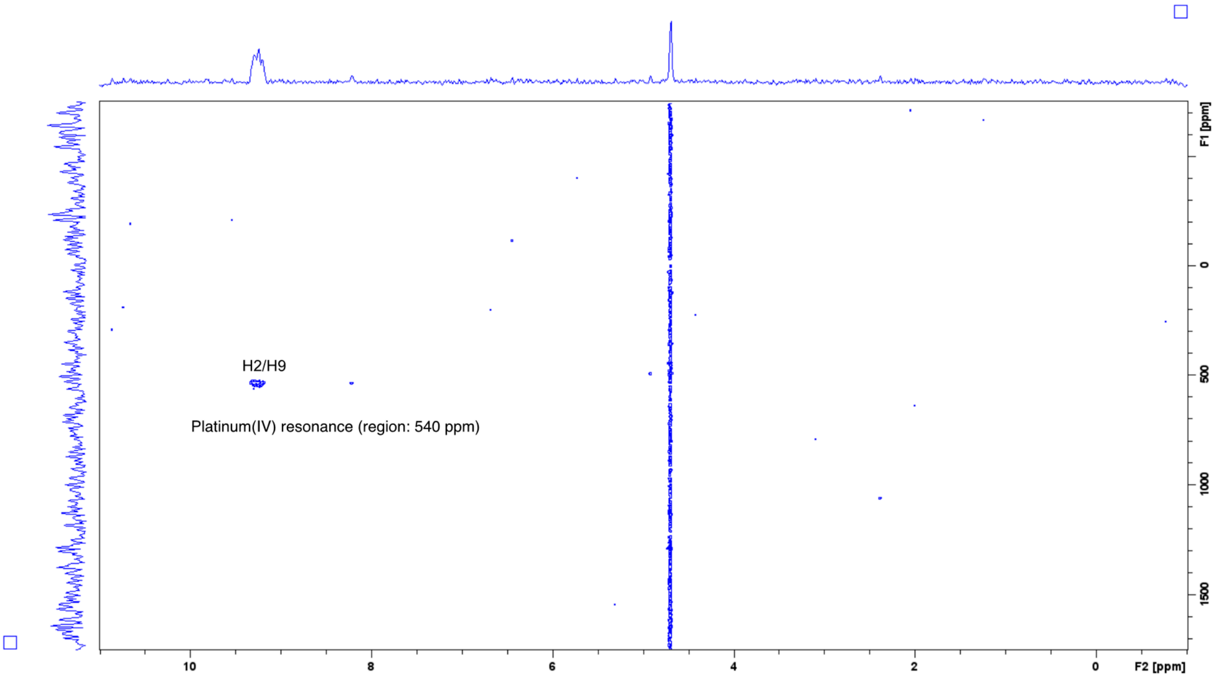

Figure N.S7. ^{1}H-^{195}Pt HMQC spectra of **Pt^{IV}56CLB** in D_2O obtained at 298 K.

Method S2. Nano Proteomics Method

The digested protein samples were analysed by UPLC-MS using a Waters nanoAcquity UPLC sample manager fitted with a binary solvent manager. Mass spectrometric detection was conducted using a Waters Synapt G2-Si. Separation consisted of two mobile phases. Mobile Phase A (0.1% formic acid in Milli-Q water) and Mobile Phase B (0.1% formic acid in ACN). The trapping column was a Waters nanoEase M/Z Symmetry C18 trap column (180 µm x 20 mm) and the analytical column was a Waters nanoAcquity UPLC 1.7um BEH130 C18 column (75 µm x 100 mm) thermostatted to 40 °C. Elution was achieved at a flow rate of 0.3 µL/min with each sample run for 55 minutes. The gradient was 0 min 1% B; 2 min 10% B; 40 min 40% B; 42 min 85% B and 50 min 85% B. System specific cleaning and equilibration protocols were run before each sample.

Mass spectrometry was conducted in positive ion mode with a capillary voltage of 3 kV and a sampling cone voltage of 30 V as well as a source offset of 30 V for electrospray ionizations. The source temperature was set at 80 °C. A desolvation source of nitrogen gas at 20 L/h and a desolvation temperature of 350 °C was used. Lock spray ion acquisition was conducted every 300 seconds with [Glu1]-fibrinopeptide B as the reference compound. Data acquisition was conducted over the mass to charge range of 50–2000. The data independent acquisition used an HDMSe experiment employing both low and high energy collision-induced dissociation of parent ions. Low energy collision was done at 6 V in the trap collision cell and at 4 V in the transfer collision cell. High energy collision used a collision energy ramp from 17 to 60 V in the transfer collision cell. Scan time was 0.5 seconds and after each scan the system would switch from high to low energy collision.

Protein identification was carried out using Progenesis QI for Proteomics and the UniProt Homo Sapiens reference database with the following conditions. The parent and fragment ion tolerances were set to automatic, the allowed maximum missed cleavages was set to 1, the allowed false discovery rate was set to 4% and the maximum protein size was set to 250 kDa. The peptide modifications were carbamidomethyl C (fixed) and oxidation M (variable). The ion matching requirements were fragments/peptide of 1 or more, fragments/protein of 3 or more and peptides/protein of 1 or more. Relative protein quantitation was performed with the Hi-N method using the top 3 peptides. Progenesis QI automatically selects unique reporter peptides to quantify a protein and measures their UPLC peak areas in the total ion chromatogram.

Table S1. Matrix Conditions selected for RASTRUM bioprinting.

Matrix Code	Formulation	Stiffness (kPa, storage modulus)	F-code (Bioink)	F-code (Activator)
Px02.09	GFOGER, RGD	1.1	F236	F177

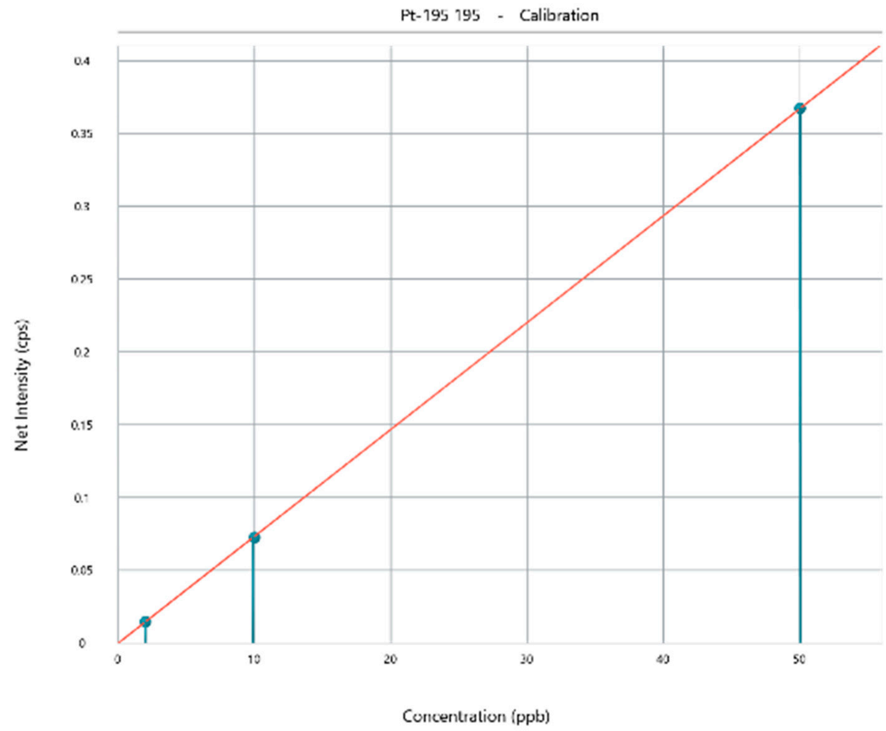

Statistics

Eqn. :	y = 0.007x + 0.000	
Cor.Coeff. :	0.999997	
BEC :	1.156310	ppb
DL :	0.009261	ppb

Calibration Table

	Net Intensity Pt-195 195 (cps)	Apparent Conc. Pt-195 195 (ppb)
Blank	6487.1	
Cal. Std.1	0.0	2.005
Cal. Std.2	0.1	9.881
Cal. Std.3	0.4	50.024

Figure S1. Calibration curve generated by plotting the peak areas measured by the ICP-MS against known concentrations (^{195}Pt(STD)). This curve was used to quantify the cellular uptake of the Pt complexes; y=0.007x + 0.000 and R^2= 0.999997

Table S2. Conditions and parameters selected on the ICP-MS machine.

**Parameter**	**Value**
Plasma RF power	1500 W
Nebulizer gas flow rate	0.92-0.94 $L.min^{-1}$
Auxiliary gas flow rate	1.2 $L.min^{-1}$
Collision gas flow rate (He)	4.5 $L.min^{-1}$
KED Cell Entrance/Exit voltage	-8/-25 V
KED CRO/QRO Voltage	-15/-12 V
Deflector Voltage	-9V
Isotope monitored	^{195}Pt
Dwell times	50 ms
Integration Time	4500 ms

RF, radio frequency; He, helium; KED, kinetic energy discrimination

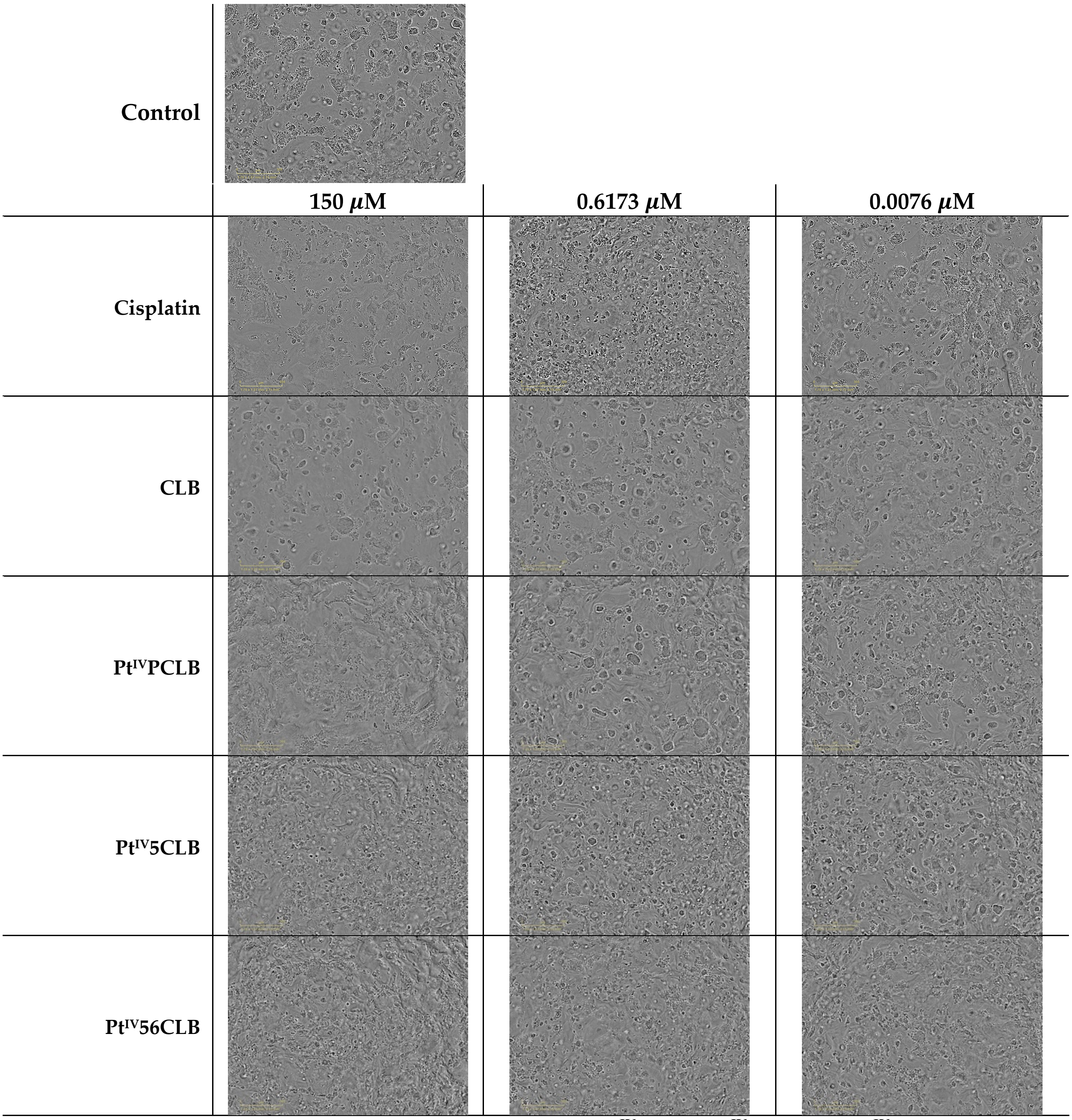

Figure S2. HT29 spheroids. Cell viability upon treatment with **Pt^{IV}PCLB**, **Pt^{IV}5CLB** and **Pt^{IV}56CLB** complexes, as well as CLB and cisplatin in **HT29** spheroids at 72 h. Incucyte® phase contrast microscope used to collect bright-field live images using 10× objective.

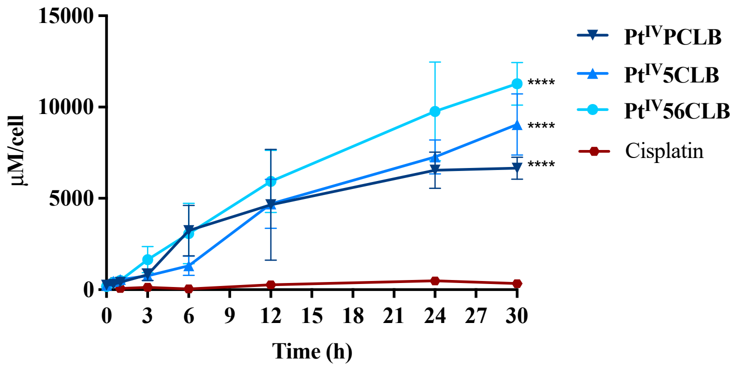

Figure S3. ICP-MS analysis for the uptake of Pt^{IV}PCLB, Pt^{IV}5CLB and **Pt^{IV}56CLB in HT29 at 0, 0.5, 1, 3, 6, 12, 24 and 30 h.** The **Pt^{II}56ME*SS*** precursor has the greatest cellular uptake in HT29. *n* = 3 from three independent experiments where samples were run in triplicates. Data points denote mean ± SEM and expressed in µM/ cell.

Table S3. Kinetic Cellular uptake of Pt complexes by HT29. Table shows the average value of cellular concentration in nmol/10^6 cells (top) and the ratio of intracellular concentration to extracellular concentrations (bottom) for each complex. *n* = 3 from three independent experiments where samples were run in triplicates. Data points denote mean ± SEM

		**Time (h)**							
	**Complex**	**0**	**0.5**	**1**	**3**	**6**	**12**	**24**	**30**
	**Pt^{IV}PCLB**	0.51	0.64	0.88	1.74	6.88	9.86	13.88	14.12
		± 0.13	± 0.15	± 0.25	± 1.13	± 5.07	± 11.15	± 3.63	± 2.21
**Intracellular Concentration (nmol/10^6 cells)**	**Pt^{IV}5CLB**	0.51	0.96	1.17	1.62	2.77	9.98	15.42	19.18
		± 0.42	± 0.51	±0.61	±0.98	±1.92	±4.95	±3.39	± 6.17
	**Pt^{IV}56CLB**	0.39	0.86	1.08	3.51	6.51	12.59	20.70	23.93
		± 0.14	± 0.49	± 0.35	± 2.63	± 6.08	± 6.29	± 9.92	± 4.32
	Cisplatin	0.36	0.94	0.14	0.28	0.10	0.57	1.02	0.71
		± 0.04	± 0.55	± 0.02	± 0.09	± 0.01	± 0.23	± 0.57	± 0.18
	**Pt^{IV}PCLB**	79.38	100.05	137.68	273.41	1080.57	1549.92	2181.45	2218.45
		±61.38	±69.32	± 69.55	± 308.52	±1381.03	±3032.95	± 987.03	±601.49
**Intracellular/ extracellular ratio**	**Pt^{IV}5CLB**	80.43	151.05	184.52	254.17	435.57	1568.11	2422.92	3014.54
		± 116.85	±138.48	±166.17	±265.87	±523.37	±1346.75	±921.21	±1678.96
	**Pt^{IV}56CLB**	60.95	135.52	169.83	550.73	1023.30	1977.32	3253.02	3760.15
		± 36.92	±132.33	± 93.95	± 716.39	±1654.58	±1711.82	±2701.73	±1174.25
	Cisplatin	57.31	147.74	22.65	43.31	16.07	89.42	160.54	112.14
		± 20.91	±258.72	± 9.82	± 46.90	± 6.78	± 106.10	± 271.72	± 83.99

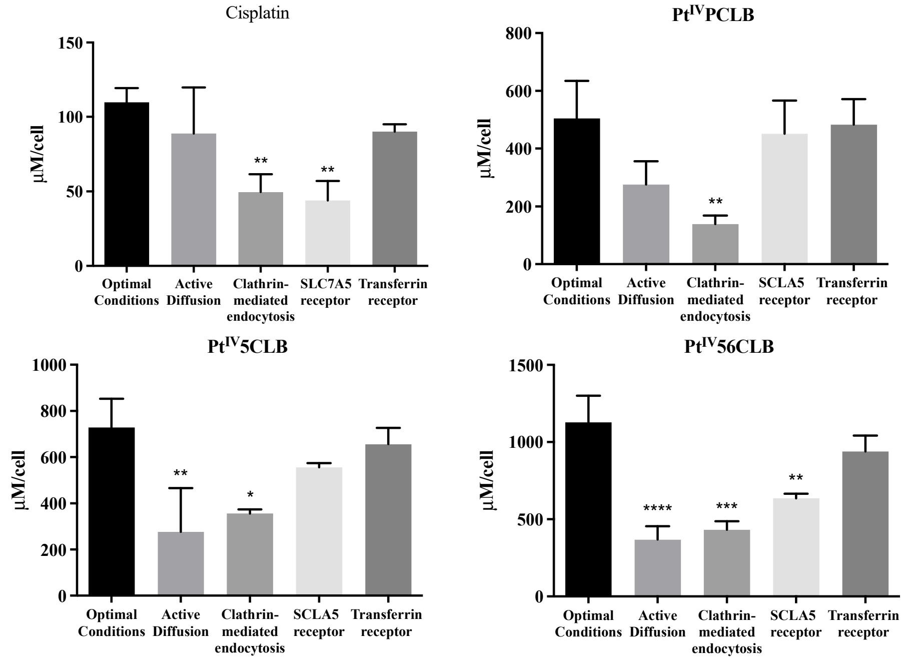

Figure S4. Mode of uptake of Platinum in HT29. The intracellular amount of Pt was measured by ICP-MS after incubation at 37 °C or 4 °C, as well as following inhibition of clathrin-mediated endocytosis, SLC7A5 receptor or transferrin receptor. Data denote mean ± SEM of three independent experiments where samples were run in triplicates and expressed in µM/ cell. **p* <0.05, ***p* < 0.01, ****p* < 0.001 and *****p* < 0.0001 in comparison to the optimal conditions.

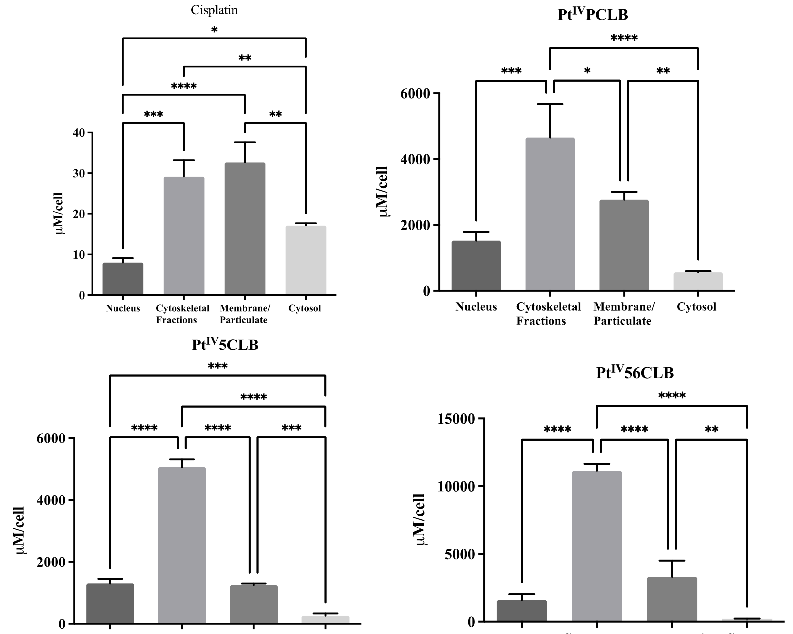

Figure S5. Cellular Localisation of Platinum in HT29. The intracellular amount of Pt (*µ*M/cell) was measured by ICP-MS after cellular fractionation. Data denote mean ± SEM of three independent experiments where samples were run in triplicates. **p* < 0.05, ***p* < 0.01, ****p* < 0.001 and *****p* < 0.0001 in comparison to the fractions.

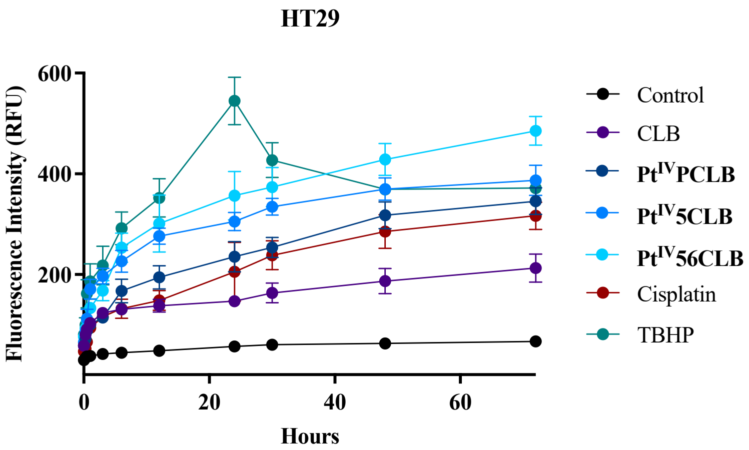

Figure. S6. ROS production upon treatment with CLB, Pt^{IV}PCLB, Pt^{IV}5CLB and Pt^{IV}56CLB complexes in HT29 at 0, 0.25, 0.5, 1, 3, 6, 12, 24, 48 and 72 hours. PCLB, **5CLB** and **56CLB** prodrugs, CLB, Cisplatin and TBHP: t-butyl hydroperoxide. Data points denote mean ± SEM. *n* = 3 from three independent experiments where samples were run in triplicate.

Table S4. ROS production upon treatment with complexes **Pt^{IV}PCLB**, **Pt^{IV}5CLB**, **Pt^{IV}56CLB** prodrugs, CLB, cisplatin and TBHP: t-butyl hydroperoxide in HT29 cells at 24, 48 and 72 h.

Complex	ROS production in different time intervals (RFU)		
	HT29		
	**24 h**	**48 h**	**72 h**
Control	57.22 ± 0.34	63.22 ± 0.30	67.33 ± 0.21
Cisplatin	205.39 ± 11.26	285.11 ± 6.49	316.55 ± 5.21
TBHP	544.67 ± 9.04	369.22 ± 2.14	371.44 ± 1.74
CLB	147.00 ± 2.25	187.00 ± 4.77	213.11 ± 5.39
**Pt^{IV}PCLB**	235.44 ± 5.78	317.77 ± 5.15	345.67 ± 5.09
**Pt^{IV}5CLB**	305.22 ± 3.49	369.77 ± 4.22	387.00 ± 5.80
**Pt^{IV}56CLB**	356.77 ± 9.27	428.67 ± 6.04	485.56 ± 5.49

Table S5. Mitochondrial membrane potential upon treatment with **Pt^{IV}PCLB**, **Pt^{IV}5CLB**, **Pt^{IV}56CLB** prodrugs, CLB, cisplatin and FCCP: carbonyl cyanide 4-(trifluoromethoxy) phenylhydrazone in HT29 cells at 24, 48 and 72 h.

Complex	MtMP at different time intervals (RFU)		
	HT29		
	**24 h**	**48 h**	**72 h**
Control	471.52 ± 19.62	398.14 ± 15.77	409.06 ± 6.18
Cisplatin	270.02 ± 10.56	237.68 ± 4.61	188.59 ± 17.95
FCCP	184.32 ± 9.51	143.31 ± 4.61	83.58 ± 3.99
CLB	368.45 ± 17.75	325.32 ± 17.89	283.67 ± 9.52
**Pt^{IV}PCLB**	214.19 ± 32.71	192.94 ± 23.96	163.35 ± 28.12
**Pt^{IV}5CLB**	163.41 ± 20.79	146.79 ± 18.33	131.55 ± 21.72
**Pt^{IV}56CLB**	121.12 ± 1.67	107.90 ± 4.09	114.31 ± 26.83

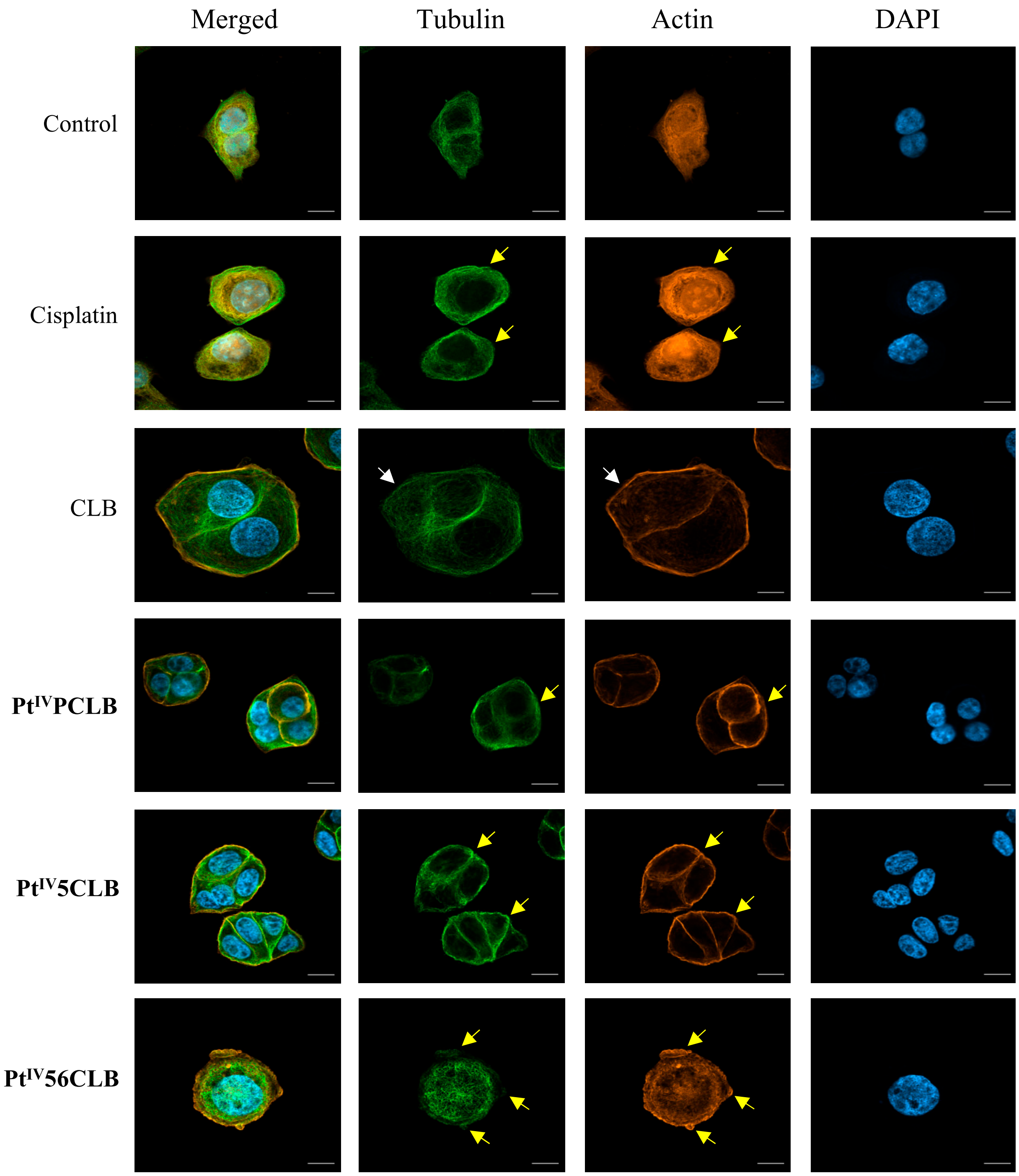

Figure S7. Effect of platinum complexes and cisplatin on β-tubulin and F-actin. Immunofluorescence upon treatment with platinum(II) and platinum(IV) complexes, as well as cisplatin in HT29. Airyscan images were collected at 63×. Yellow arrows indicate the membrane blebbing observed across treatments. White arrows indicate necrotic cell swelling across treatments. Data were analysed from triplicate experiments at n = 30 cells per replicate for each treatment.

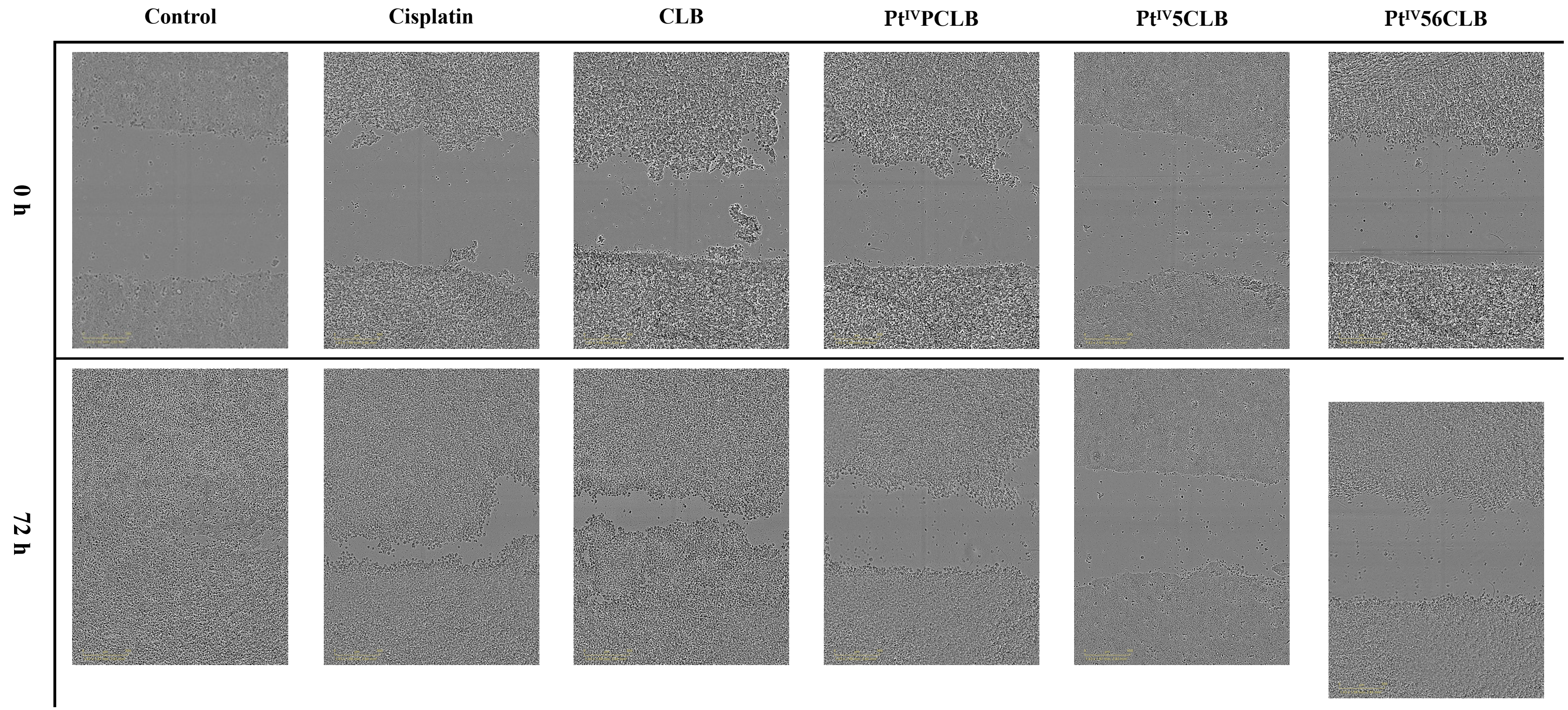

Figure S8. Cell wound healing post treatment. Represenattive wound images at 0 and 72 h of treatemnte with cisplatin, CLB, **Pt^{IV}PCLB**, **Pt^{IV}5CLB** and **Pt^{IV}56CLB** in **HT29** cells. Incucyte® phase contrast microscope used to collect bright-field live images using 10× objective.

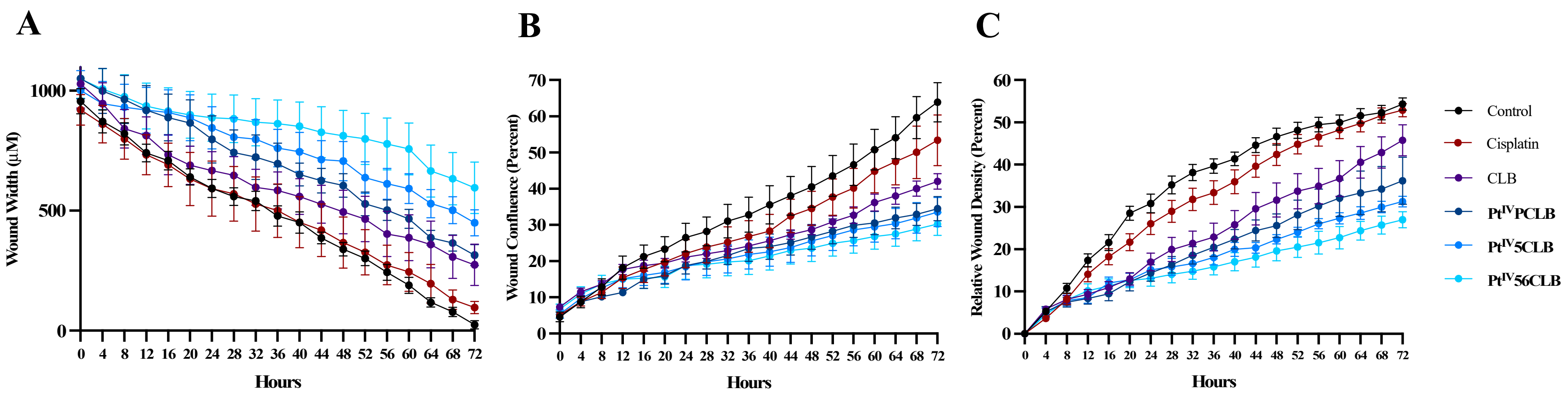

Figure S9. Wound Closure. Percent wound width (A), wound confluence (B) and relative wound density (C) measured upon treatment with **Pt^{IV}PCLB**, **Pt^{IV}5CLB** and **Pt^{IV}56CLB** prodrugs, as well as CLB and cisplatin in HT29 cells quantified every 4 h for up to 72 h

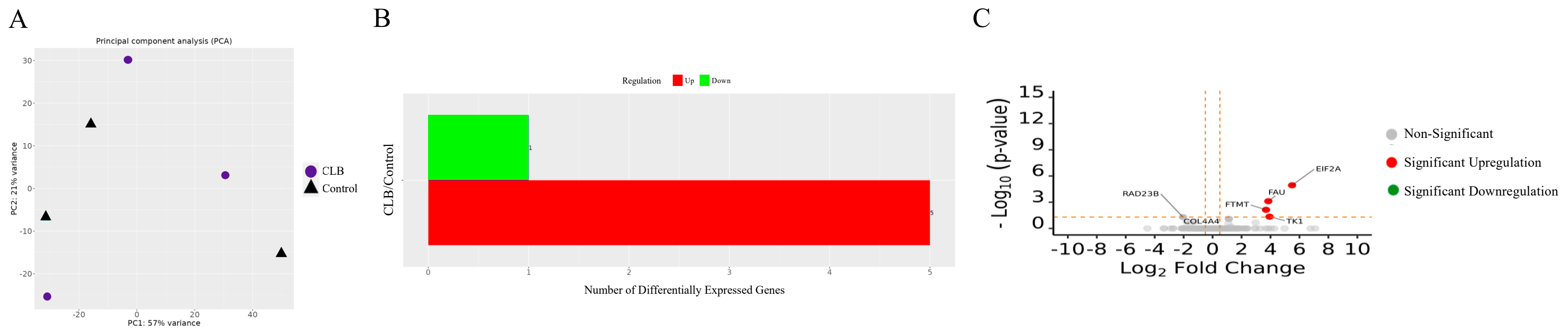

Figure S10. Proteomic analysis of HT29 upon treatment with CLB. A. principal component analysis. B. Number of differentially expressed proteins (DEPs). C. Volcano plot of DEPs upregulated (red) and down regulated (green) proteins.

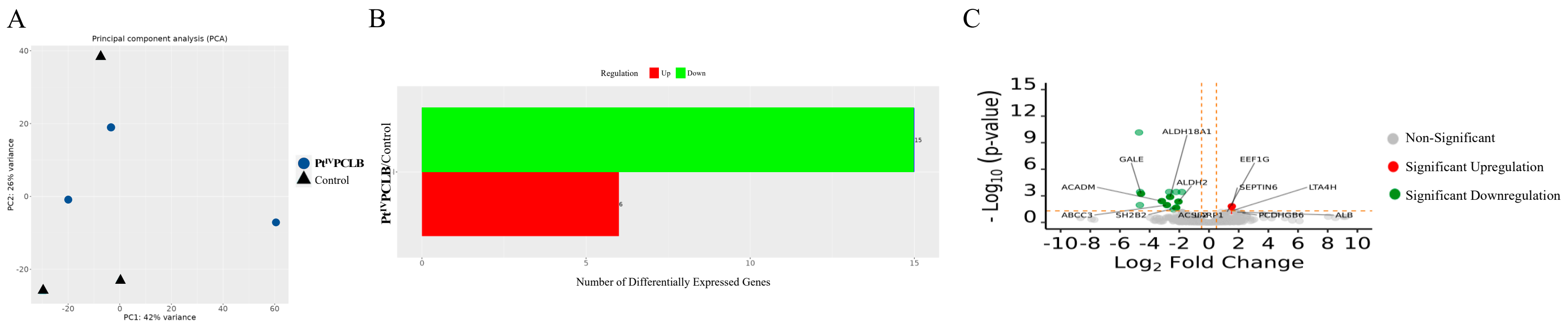

Figure S11. Proteomic analysis of HT29 upon treatment with **Pt^{IV}PCLB**. A. principal component analysis. B. Number of differentially expressed proteins (DEPs). C. Volcano plot of DEPs upregulated (red) and down regulated (green) proteins.

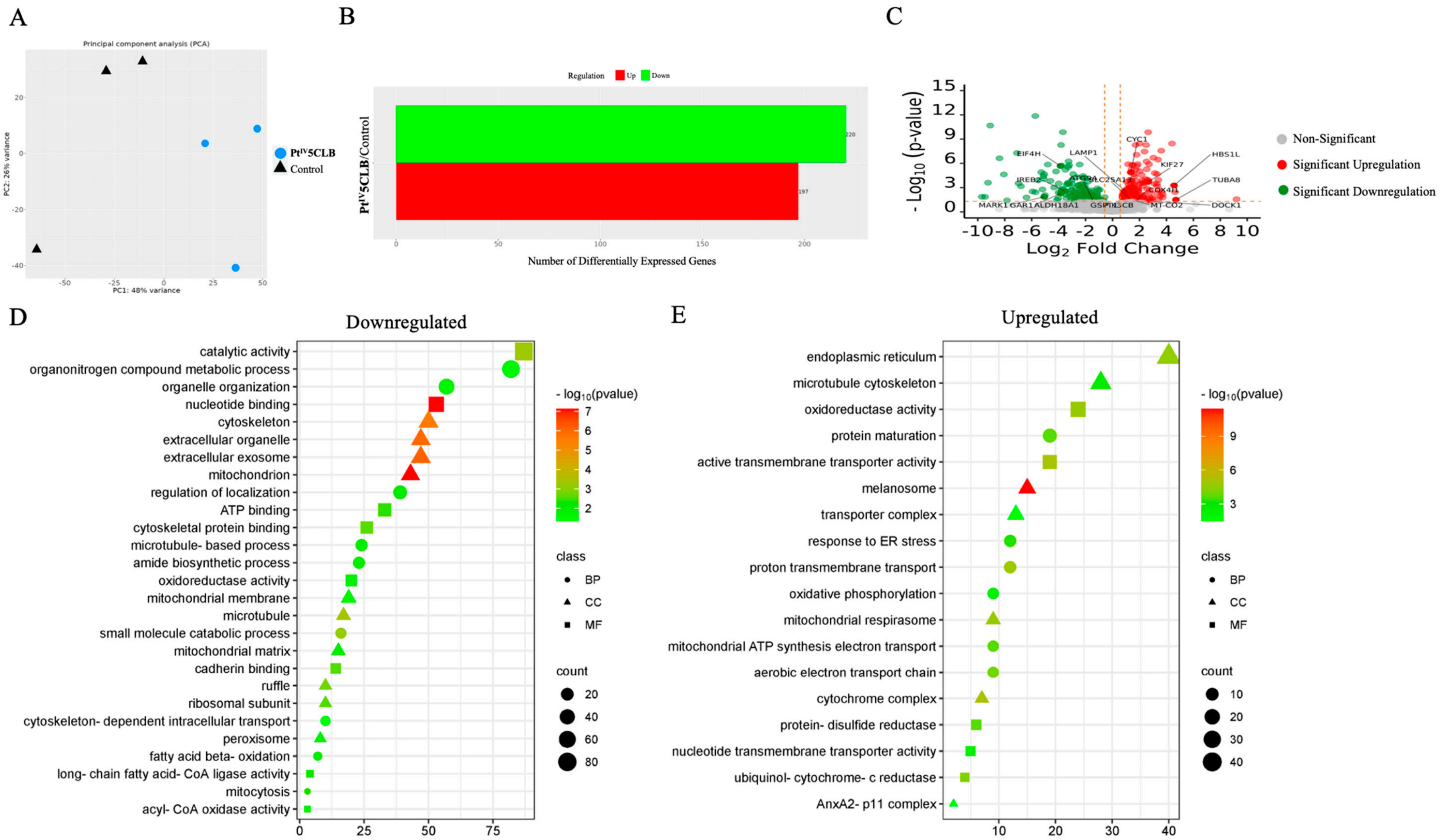

A
Principal component analysis (PCA)
PC1: 48% variance
PC2: 26% variance
Pt^IV5CLB
Control
B
Regulation Up Down
Pt^IV5CLB/Control
Number of Differentially Expressed Genes
220
197
C
- Log10 (p-value)
Log2 Fold Change
Non-Significant
Significant Upregulation
Significant Downregulation
EIF4H
LAMP1
CYC1
HBS1L
KIF27
TUBA8
IREB2
COX4I1
MARK1
GAR1
ALDH18A1
MT-CO2
DOCK1
D
Downregulated
catalytic activity
organonitrogen compound metabolic process
organelle organization
nucleotide binding
cytoskeleton
extracellular organelle
extracellular exosome
mitochondrion
regulation of localization
ATP binding
cytoskeletal protein binding
microtubule- based process
amide biosynthetic process
oxidoreductase activity
mitochondrial membrane
microtubule
small molecule catabolic process
mitochondrial matrix
cadherin binding
ruffle
ribosomal subunit
cytoskeleton- dependent intracellular transport
peroxisome
fatty acid beta- oxidation
long- chain fatty acid- CoA ligase activity
mitocytosis
acyl- CoA oxidase activity
- log10(pvalue)
class
BP
CC
MF
count
E
Upregulated
endoplasmic reticulum
microtubule cytoskeleton
oxidoreductase activity
protein maturation
active transmembrane transporter activity
melanosome
transporter complex
response to ER stress
proton transmembrane transport
oxidative phosphorylation
mitochondrial respirasome
mitochondrial ATP synthesis electron transport
aerobic electron transport chain
cytochrome complex
protein- disulfide reductase
nucleotide transmembrane transporter activity
ubiquinol- cytochrome- c reductase
AnxA2- p11 complex
- log10(pvalue)
class
BP
CC
MF
count

F

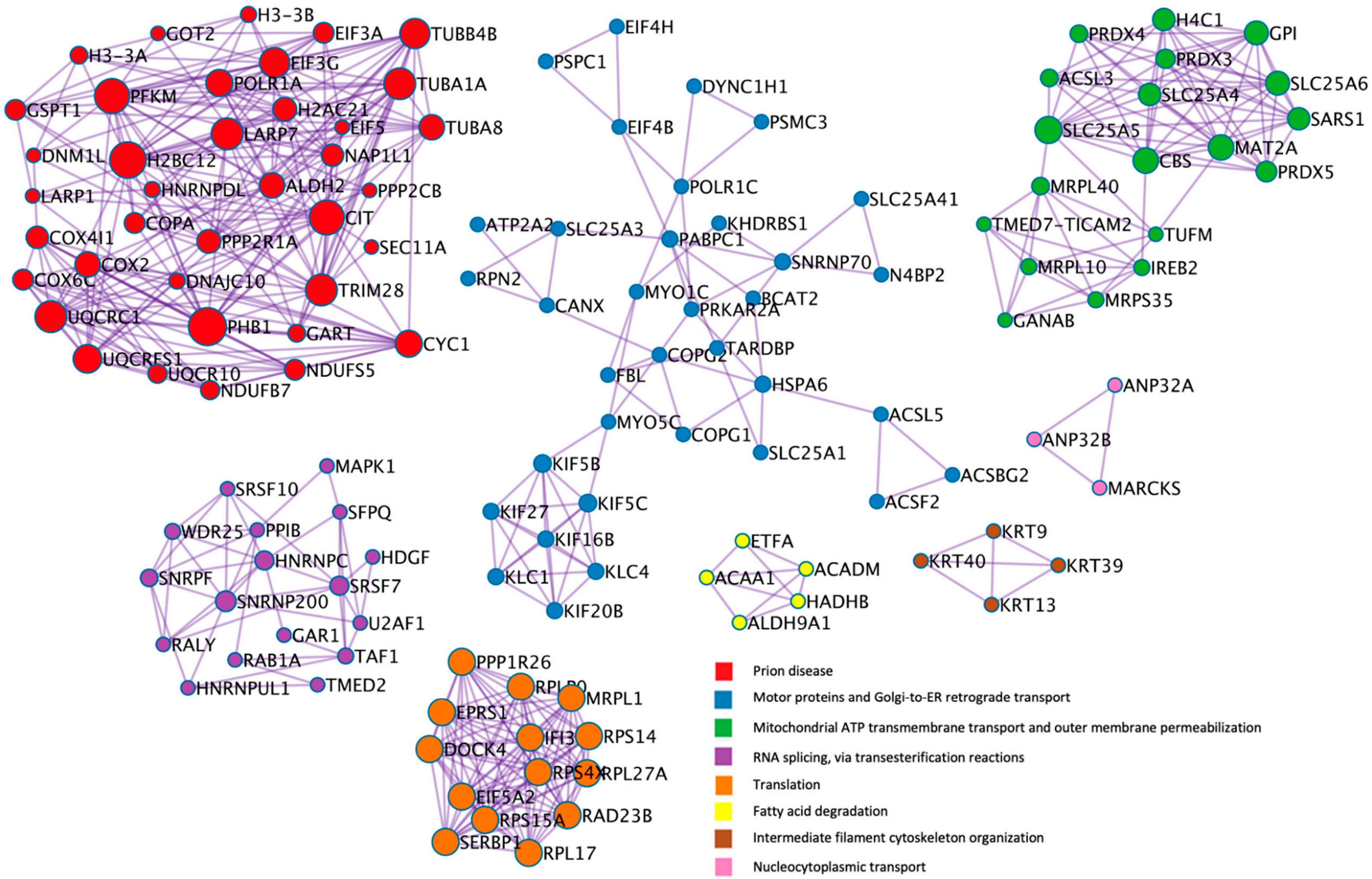

Figure S12. Proteomic analysis of HT29 upon treatment with Pt^{IV}5CLB. A. HT29 principal component analysis. B. HT29 number of differentially expressed proteins (DEPs). C. HT29 volcano plot of DEPs upregulated (red) and down regulated (green). GO enriched biological processes, cellular components, and molecular function in HT29, D. Downregulated and E. Upregulated proteins. F. HT29 pathway enrichment and gene act network analysis in most significance of dysregulated pathways. Data points denote mean ± SEM. *n* = 3 from three separate experiments.

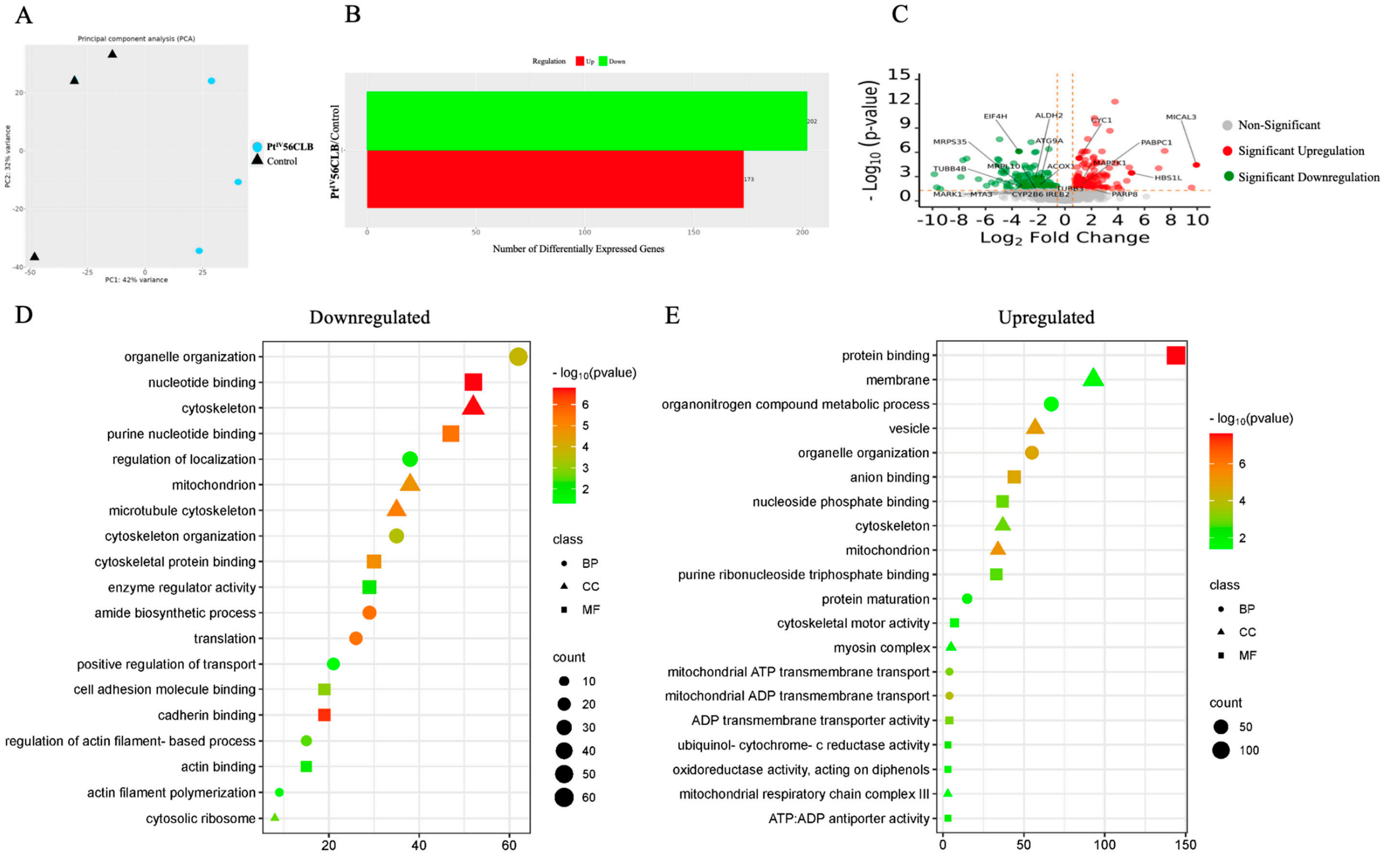
A
Principal component analysis (PCA)
PC1: 42% variance
PC2: 32% variance
Pt^IV56CLB
Control
B
Regulation
Up
Down
Pt^IV56CLB/Control
202
173
Number of Differentially Expressed Genes
C
- Log10 (p-value)
Log2 Fold Change
EIF4H
ALDH2
CYC1
MICAL3
MRPS35
ATG9A
PABPC1
TUBB4B
MRPL10
ACOX1
MAP2K1
HBS1L
MARK1
MTA3
CYP2B6
IREB2
TUBB3
PARP8
Non-Significant
Significant Upregulation
Significant Downregulation
D
Downregulated
organelle organization
nucleotide binding
cytoskeleton
purine nucleotide binding
regulation of localization
mitochondrion
microtubule cytoskeleton
cytoskeleton organization
cytoskeletal protein binding
enzyme regulator activity
amide biosynthetic process
translation
positive regulation of transport
cell adhesion molecule binding
cadherin binding
regulation of actin filament- based process
actin binding
actin filament polymerization
cytosolic ribosome
- log10(pvalue)
class
BP
CC
MF
count
E
Upregulated
protein binding
membrane
organonitrogen compound metabolic process
vesicle
organelle organization
anion binding
nucleoside phosphate binding
cytoskeleton
mitochondrion
purine ribonucleoside triphosphate binding
protein maturation
cytoskeletal motor activity
myosin complex
mitochondrial ATP transmembrane transport
mitochondrial ADP transmembrane transport
ADP transmembrane transporter activity
ubiquinol- cytochrome- c reductase activity
oxidoreductase activity, acting on diphenols
mitochondrial respiratory chain complex III
ATP:ADP antiporter activity
- log10(pvalue)
class
BP
CC
MF
count

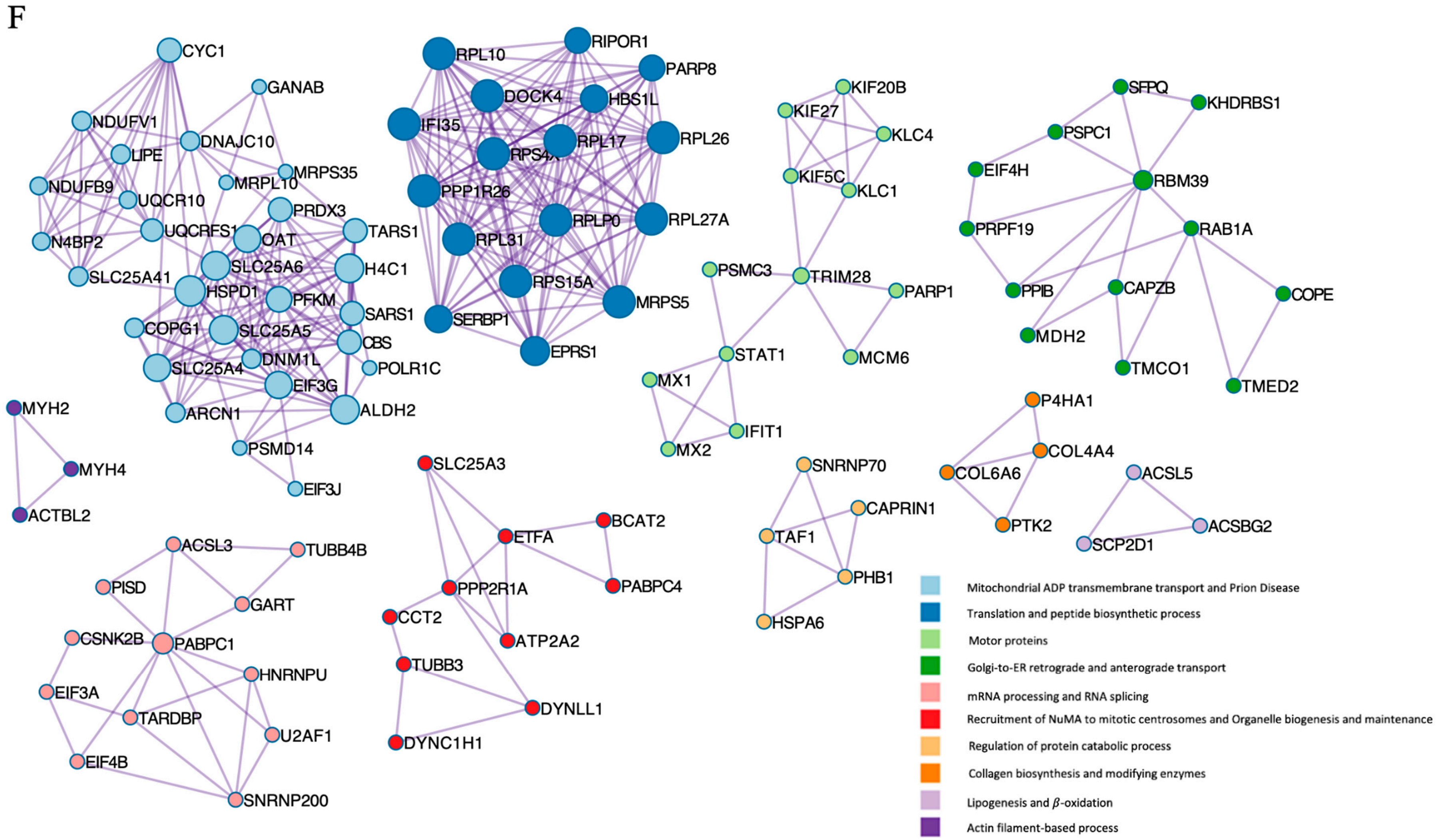

Figure S13. Proteomic analysis of HT29 upon treatment with Pt^{IV}56CLB. A. HT29 principal component analysis. B. HT29 number of differentially expressed proteins (DEPs). C. HT29 volcano plot of DEPs upregulated (red) and down regulated (green). GO enriched biological processes, cellular components, and molecular function in HT29, D. Downregulated and E. Upregulated proteins. F. HT29 pathway enrichment and gene act network analysis in most significance of dysregulated pathways. Data points denote mean ± SEM. n = 3 from three separate experiments.

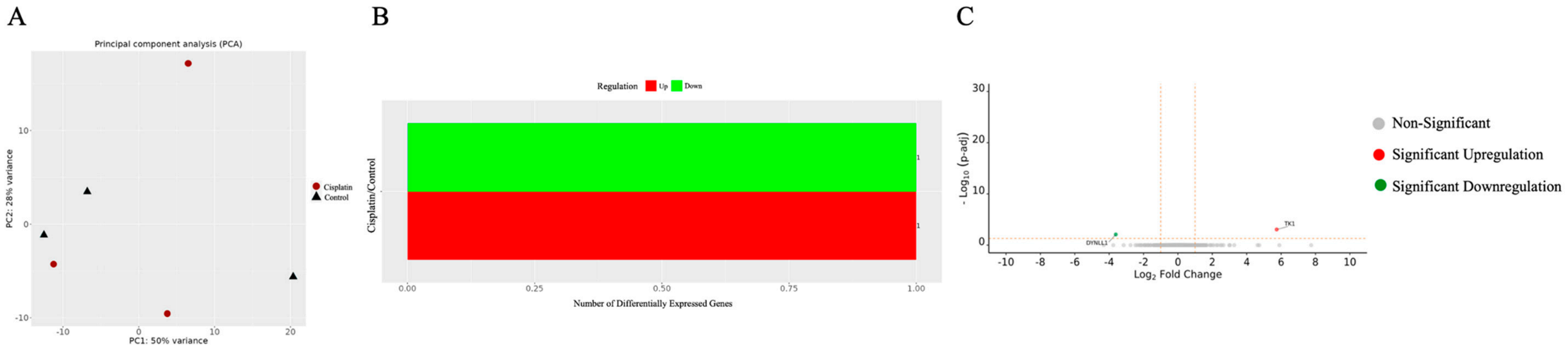

Figure S14. Proteomic analysis of HT29 upon treatment with cisplatin. A. HT29 principal component analysis. B. HT29 number of differentially expressed proteins (DEPs). C. HT29 volcano plot of DEPs upregulated (red) and down regulated (green). Data points denote mean ± SEM. n = 3 from three separate experiment.

Table S6. Antibody Concentrations used in Western Blot

Supplier	Clone no.	Clone	Primary Antibody Target	Molecular Weight
Abcam	ab32503	Rabbit monoclonal	BAX	21 KDa
Abcam	ab196495	Rabbit polyclonal	Bcl2	26 KDa
Abcam	ab32138	Rabbit monoclonal	PARP-1	pro-form 113 Kda and *25* Kda (N-terminal catalytic domain) *cleaved* form of *PARP1*
Abcam	ab133504	Rabbit monoclonal	Cytochrome C	11 or 14 KDa
Abcam	ab214430	Rabbit monoclonal	Caspase 3	32 Kda and cleaved fragments at 17, 19 or 24 Kda
Abcam	ab108333	Rabbit monoclonal	Caspase 8	55 KDa
Abcam	ab202068	Rabbit monoclonal	Caspase 9	46, KDa
Abcam	ab8227	Rabbit polyclonal	Beta Actin	42 KDa
Abcam	ab7291	Mouse monoclonal	Alpha Tubulin	50 KDa
Abcam	ab6046	Rabbit Polyclonal	Beta Tubulin	50 KDa
Abcam	ab32389	Rabbit monoclonal	p53	44 KDa
Abcam	ab109520	Rabbit monoclonal	p21	21 KDa
Abcam	ab192591	Rabbit polyclonal	p-ERK	41-44 KDa
Abcam	ab184699	Rabbit monoclonal	ERK	42 KDa
Abcam	ab38449	Rabbit polyclonal	p-AKT	56 KDa
Abcam	ab8805	Rabbit polyclonal	AKT	56 KDa
Abcam	ab8245	Mouse monoclonal	GAPDH	36 or 40 KDa
Abcam	ab228668	Rabbit polyclonal	APG5L/ATG5	predicted at 32 KDA but seen at 55 Kda
Abcam	ab228525	Rabbit polyclonal	ATG16L1 - N-terminal	68-70 Kda
			ATG4B	44 Kda
			ATG9A	94-100 Kda
			Beclin-1	52 Kda
			LC3B	14-16 Kda
Abcam	ab10640	Rabbit polyclonal	Bid Cleavage Site	15 KDa

The representative full blots below include proteins from several projects run in parallel. Proteins related to this study are labelled per lane for the relevant treatment.

A

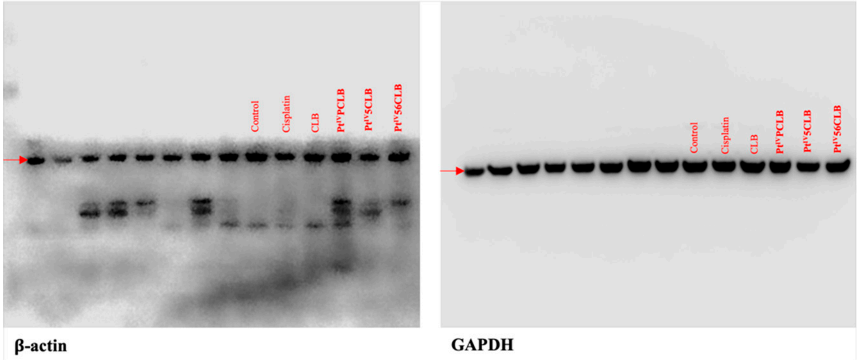

β-actin/ GAPDH normalized to control	**Control**	**Cisplatin**	**CLB**	**Pt^{IV}PCLB**	**Pt^{IV}5CLB**	**Pt^{IV}56CLB**
	1	1.044601	1.071699	1.107864	1.085357	1.115991

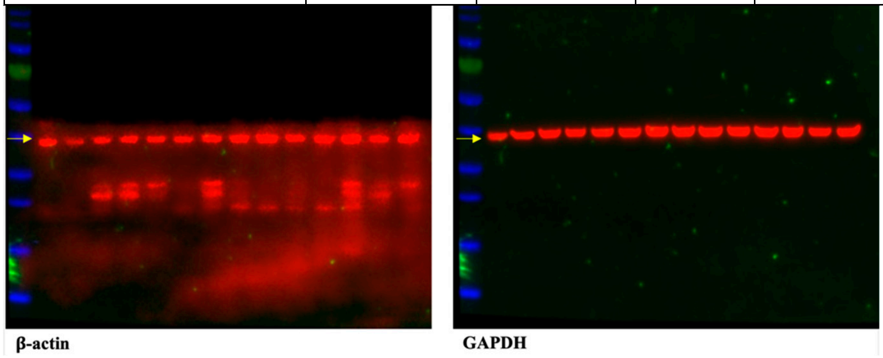

Representative uncropped western blot is presented with protein of interest directed with a red arrow and lanes marked left to right, control, cisplatin, CLB, **Pt^{IV}PCLB**, **Pt^{IV}5CLB** and **Pt^{IV}56CLB** in red, on the chemiluminescence blot. Representative fluorescent western blot with molecular marker (PageRuler Prestained Protein Ladder (Invitrogen #26617)) was detected using laser 600 and 700 on the Odyssey® FC imaging system and protein of interest directed with a yellow arrow.

B

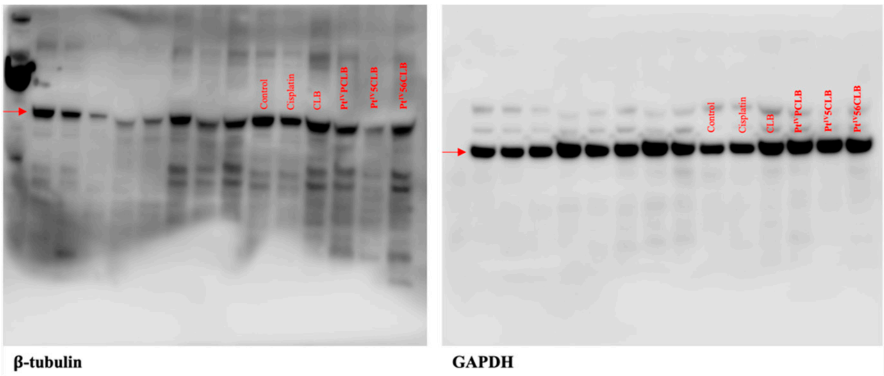

β-tubulin/ GAPDH normalized to control	**Control**	**Cisplatin**	**CLB**	**Pt^{IV}PCLB**	**Pt^{IV}5CLB**	**Pt^{IV}56CLB**
	1	0.797417	0.924209	0.613801	0.249065	0.551983

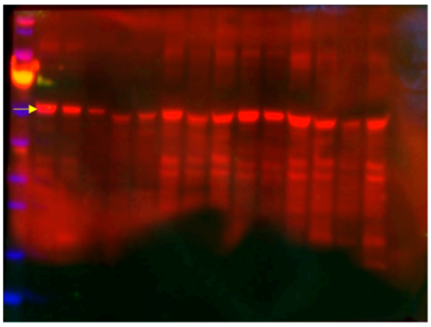

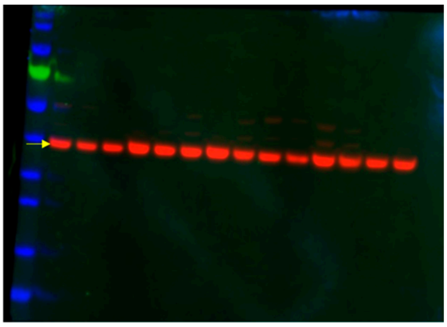

β-tubulin **GAPDH**

Representative uncropped western blot is presented with protein of interest directed with a red arrow and lanes marked left to right, control, cisplatin, CLB, **Pt^{IV}PCLB**, **Pt^{IV}5CLB** and **Pt^{IV}56CLB** in red, on the chemiluminescence blot. Representative fluorescent western blot with molecular marker (PageRuler Prestained Protein Ladder (Invitrogen #26617)) was detected using laser 600 and 700 on the Odyssey® FC imaging system and protein of interest directed with a yellow arrow.

C

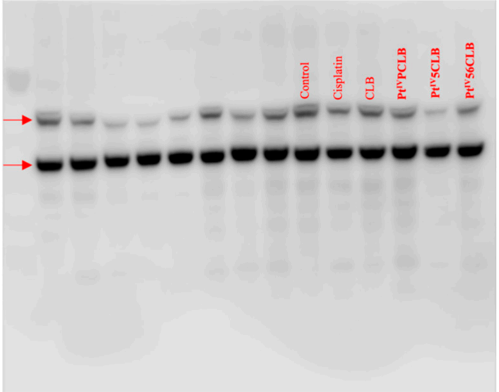

α-tubulin –top
GAPDH -bottom

α-tubulin/ GAPDH normalized to control	**Control**	**Cisplatin**	**CLB**	**Pt^{IV}PCLB**	**Pt^{IV}5CLB**	**Pt^{IV}56CLB**
	1	0.608245	0.914767	0.653298	0.226217	0.546711

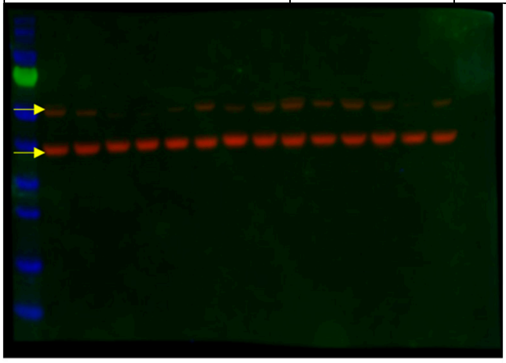

α-tubulin –top
GAPDH -bottom

Representative uncropped western blot is presented with protein of interest directed with a red arrow and lanes marked left to right, control, cisplatin, CLB, **Pt^{IV}PCLB**, **Pt^{IV}5CLB** and **Pt^{IV}56CLB** in red, on the chemiluminescence blot. Representative fluorescent western blot with molecular marker (PageRuler Prestained Protein Ladder (Invitrogen #26617)) was detected using laser 600 and 700 on the Odyssey® FC imaging system and protein of interest directed with a yellow arrow.

Figure S15. Full representative western blot of microtubule cytoskeleton protein markers in HT29. Represented data normalized to GAPDH relative to control. A. β-actin, B. β-tubulin and C. α-tubulin. Protein expression upon treatment with CLB ligand, **Pt^{IV}PCLB**, **Pt^{IV}5CLB** and **Pt^{IV}56CLB** prodrugs, as well as cisplatin in HT29 cells at 72 h compared with control.

A

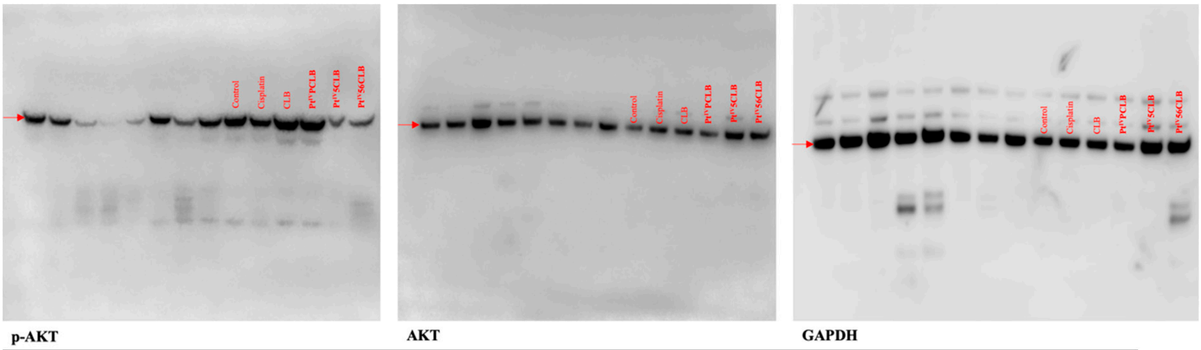

p-AKT/AKT/GAPDH normalized to control	Control	Cisplatin	CLB	Pt^{IV}PCLB	Pt^{IV}5CLB	Pt^{IV}56CLB
	1	0.679269	0.750394	0.652583	0.148024	0.209717

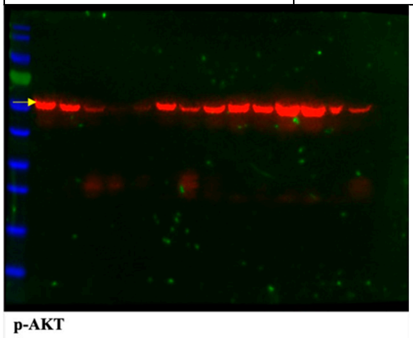

Representative uncropped western blot is presented with protein of interest directed with a red arrow and lanes marked left to right, control, cisplatin, CLB, **Pt^{IV}PCLB**, **Pt^{IV}5CLB** and **Pt^{IV}56CLB** in red, on the chemiluminescence blot. Representative fluorescent western blot of GAPDH with molecular marker (PageRuler Prestained Protein Ladder (Invitrogen #26617)) was detected using laser 600 and 700 on the Odyssey® FC imaging system and protein of interest directed with a yellow arrow. AKT and GAPDH molecular marker can be seen on the chemiluminescence blot and tracked using the p-AKT fluorescent blot, given AKT will fall at the same molecular weight and GAPDH will fall below.

B

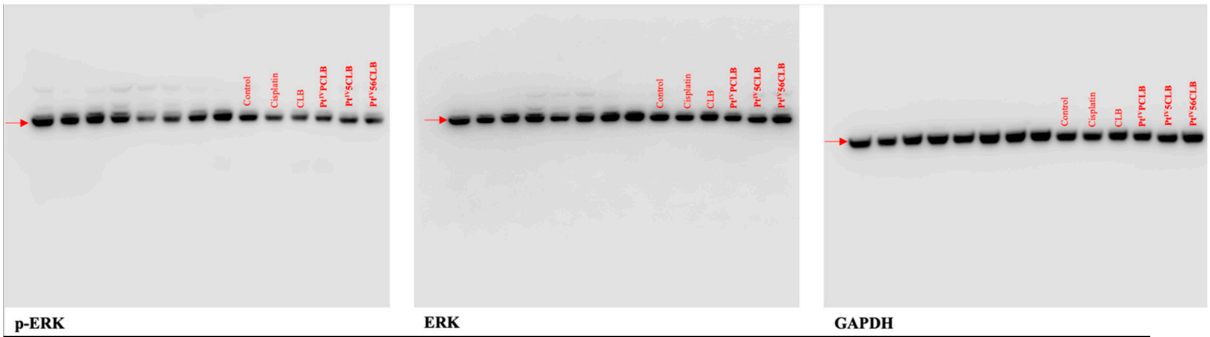

p-ERK/ERK/GAPDH normalized to control	Control	Cisplatin	CLB	Pt^{IV}PCLB	Pt^{IV}5CLB	Pt^{IV}56CLB
	1	0.891089	1.060057	0.840479	0.708521	0.820284

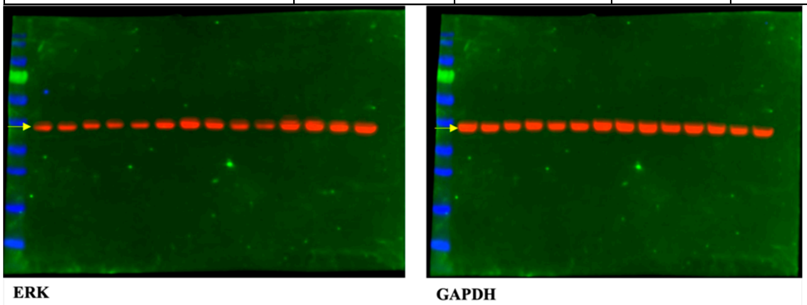

Representative uncropped western blot is presented with protein of interest directed with a red arrow and lanes marked left to right, control, cisplatin, CLB, **Pt^{IV}PCLB**, **Pt^{IV}5CLB** and **Pt^{IV}56CLB** in red, on the chemiluminescence blot. Representative fluorescent western blot of ERK and GAPDH with molecular marker

(PageRuler Prestained Protein Ladder (Invitrogen #26617)) was detected using laser 600 and 700on the Odyssey® FC imaging system and protein of interest directed with a yellow arrow. p-ERK molecular marker can be seen on the chemiluminescence blot and tracked using the ERK fluorescent blot, given p-ERK will fall at the same molecular weight.

C

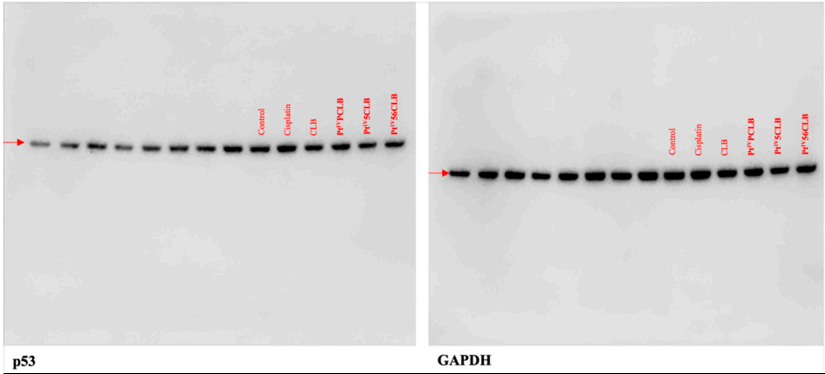

p53/GAPDH normalized to control	**Control**	**Cisplatin**	**CLB**	**Pt^{IV}PCLB**	**Pt^{IV}5CLB**	**Pt^{IV}56CLB**
	1	0.996447	0.897443	1.269731	1.030841	0.960249

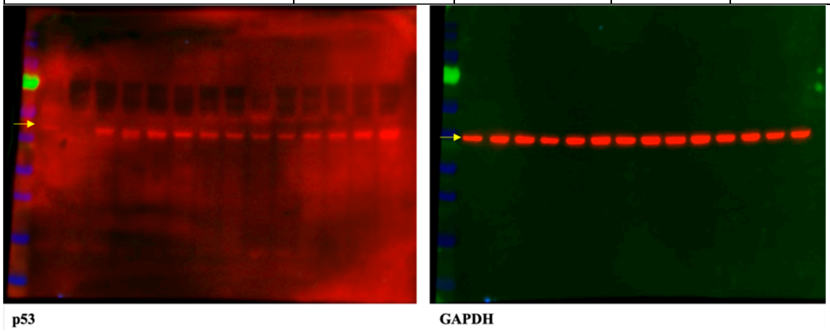

Representative uncropped western blot is presented with protein of interest directed with a red arrow and lanes marked left to right, control, cisplatin, CLB, **Pt^{IV}PCLB**, **Pt^{IV}5CLB** and **Pt^{IV}56CLB** in red, on the chemiluminescence blot. Representative fluorescent western blot with molecular marker (PageRuler Prestained Protein Ladder (Invitrogen #26617)) was detected using laser 600 and 700 on the Odyssey® FC imaging system and protein of interest directed with a yellow arrow.

D

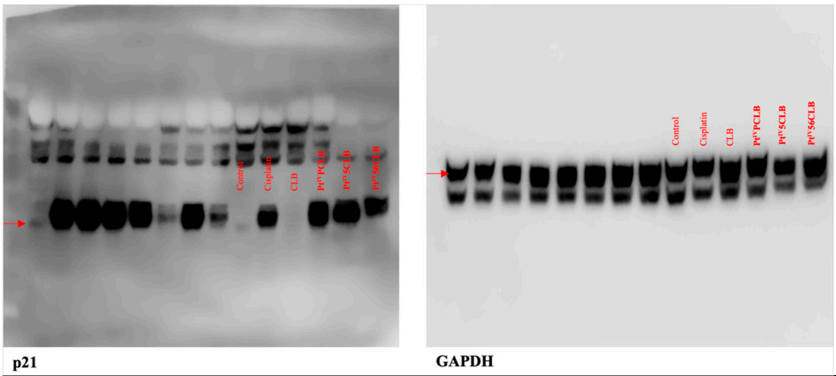

p21/GAPDH normalized to control	**Control**	**Cisplatin**	**CLB**	**Pt^{IV}PCLB**	**Pt^{IV}5CLB**	**Pt^{IV}56CLB**
	1	17.897862	1.213588	19.088203	22.959062	20.046792

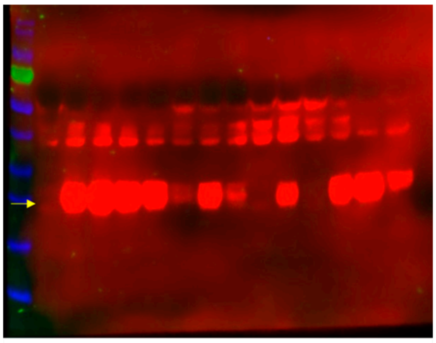

p21

Representative uncropped western blot is presented with protein of interest directed with a red arrow and lanes marked left to right, control, cisplatin, CLB, **Pt^{IV}PCLB**, **Pt^{IV}5CLB** and **Pt^{IV}56CLB** in red, on the chemiluminescence blot. Representative fluorescent western blot of p21 with molecular marker (PageRuler Prestained Protein Ladder (Invitrogen #26617)) was detected using laser 600 and 700 on the Odyssey® FC imaging system and protein of interest directed with a yellow arrow. GAPDH molecular marker fluorescent blot was not obtained but can be seen on the chemiluminescence blot and tracked using the Bcl2 fluorescent blot, given GAPDH will fall above.

Figure S16. Full representative western blot of cell proliferation protein markers in HT29. Represented data normalized to GAPDH relative to control. A. p-AKT/AKT, B. p-ERK/ERK, C. p53 and D. p21. Protein expression upon treatment with CLB ligand, **Pt^{IV}PCLB**, **Pt^{IV}5CLB** and **Pt^{IV}56CLB** prodrugs, as well as cisplatin in HT29 cells at 72 h compared with control.

A

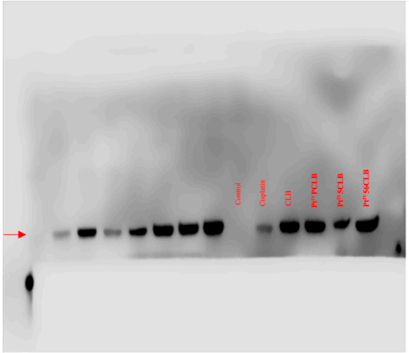

Bax

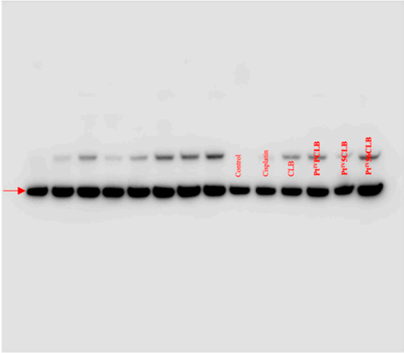

GAPDH

Bax/GAPDH normalized to control	**Control**	**Cisplatin**	**CLB**	**Pt^{IV}PCLB**	**Pt^{IV}5CLB**	**Pt^{IV}56CLB**
	1	3.575529	11.299514	13.200309	9.344941	15.002152

Representative uncropped western blot is presented with protein of interest directed with a red arrow and lanes marked left to right, control, cisplatin, CLB, **Pt^{IV}PCLB**, **Pt^{IV}5CLB** and **Pt^{IV}56CLB** in red, on the chemiluminescence blot. Representative fluorescent western blot could not be detected with fault in lasers 600 and 700 of Odyssey® FC imaging system requiring replacement. However, manual confirmation was done against the molecular marker (SeeBlue Prestained Protein Standard (Invitrogen #LC5625)) loaded to the left to confirm the detected band position, given previous knowledge of where the correct band falls for Bax protein detection [5]. This was additionally referenced to antibodies description in Table S6, given previous observational knowledge of GAPDH fluorescent blots.

B

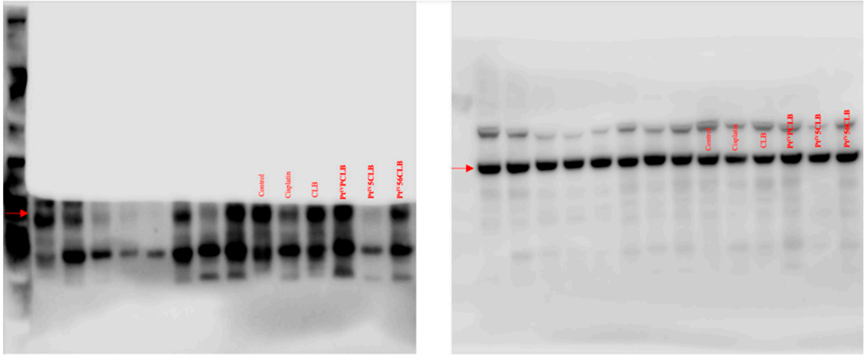

Bcl2 GAPDH

Bcl2/GAPDH normalized to control	Control	Cisplatin	CLB	Pt^{IV}PCLB	Pt^{IV}5CLB	Pt^{IV}56CLB
	1	0.361737	0.854015	0.819861	0.119659	0.367151

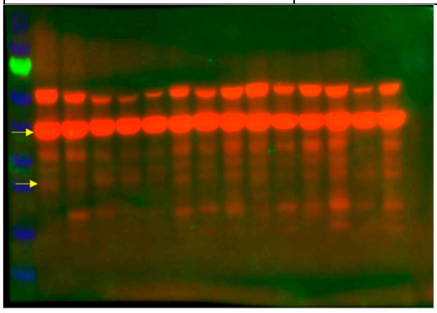

Bcl2 -top
GAPDH -bottom

Representative uncropped western blot is presented with protein of interest directed with a red arrow and lanes marked left to right, control, cisplatin, CLB, **Pt^{IV}PCLB**, **Pt^{IV}5CLB** and **Pt^{IV}56CLB** in red, on the chemiluminescence blot. Representative fluorescent western blot with molecular marker (PageRuler Prestained Protein Ladder (Invitrogen #26617)) was detected using laser 600 and 700 on the Odyssey® FC imaging system and protein of interest directed with a yellow arrow.

C

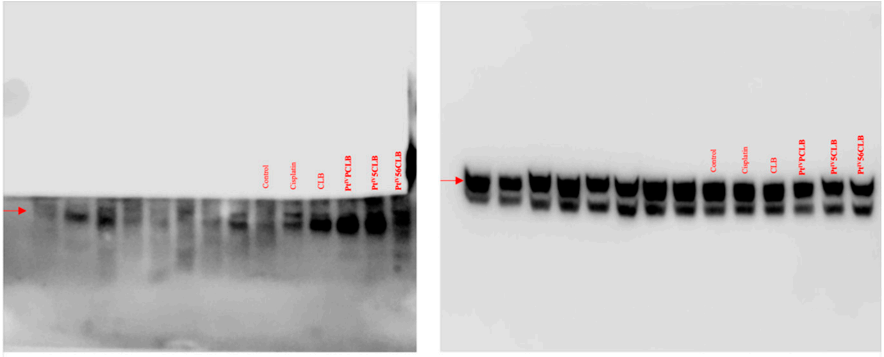

Cytochrome C GAPDH

Cytochrome C/GAPDH normalized to control	Control	Cisplatin	CLB	Pt^{IV}PCLB	Pt^{IV}5CLB	Pt^{IV}56CLB
	1	2.728916	7.911619	9.641249	11.014899	7.068662

Representative uncropped western blot is presented with protein of interest directed with a red arrow and lanes marked left to right, control, cisplatin, CLB, **Pt^{IV}PCLB**, **Pt^{IV}5CLB** and **Pt^{IV}56CLB** in red, on the chemiluminescence blot. Representative fluorescent western blot could not be detected with fault in lasers 600 and 700 of Odyssey® FC imaging system requiring replacement. However, manual confirmation was done against the molecular marker (SeeBlue Prestained Protein Standard (Invitrogen #LC5625)) loaded to the left to confirm the detected band position, given previous knowledge of where the correct band falls for Cytochrome C protein detection [5]. This was additionally referenced to antibodies description in Table S6, given previous observational knowledge of GAPDH fluorescent blots.

D

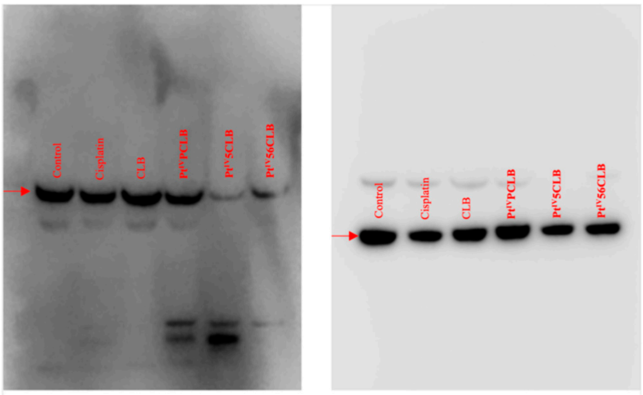

Procaspase 8 **GAPDH**

Procaspase 8/GAPDH normalized to control	**Control**	**Cisplatin**	**CLB**	**Pt^{IV}PCLB**	**Pt^{IV}5CLB**	**Pt^{IV}56CLB**
	1	0.940767	1.079379	1.096038	0.184541	0.260416

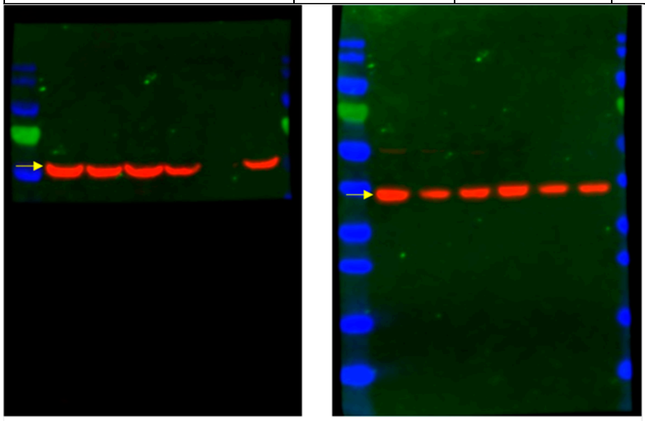

Procaspase 8 **GAPDH**

Representative uncropped western blot is presented with protein of interest directed with a red arrow and lanes marked left to right, control, cisplatin, CLB, **Pt^{IV}PCLB**, **Pt^{IV}5CLB** and **Pt^{IV}56CLB** in red, on the chemiluminescence blot. Representative fluorescent western blot with molecular marker (PageRuler Prestained Protein Ladder (Invitrogen #26617)) was detected using laser 600 and 700 on the Odyssey® FC imaging system and protein of interest directed with a yellow arrow.

E

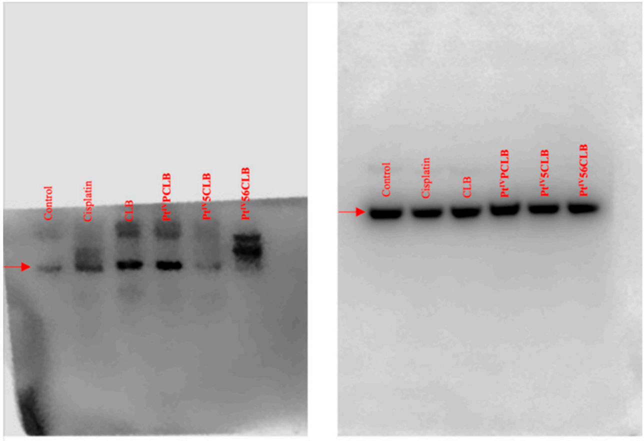

Cleaved BID **GAPDH**

Cleaved BID/GAPDH normalized to control	**Control**	**Cisplatin**	**CLB**	**Pt^{IV}PCLB**	**Pt^{IV}5CLB**	**Pt^{IV}56CLB**
	1	1.385367	3.589048	3.796081	1.199393	4.880931

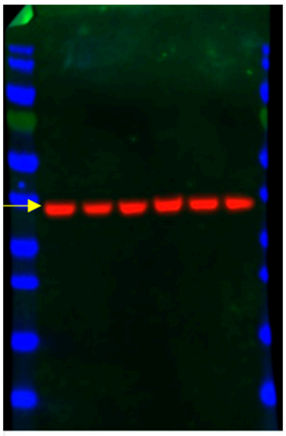

GAPDH

Representative uncropped western blot is presented with protein of interest directed with a red arrow and lanes marked left to right, control, cisplatin, CLB, **Pt^{IV}PCLB**, **Pt^{IV}5CLB** and **Pt^{IV}56CLB** in red, on the chemiluminescence blot. Representative fluorescent western blot of GAPDH with molecular marker (PageRuler Prestained Protein Ladder (Invitrogen #26617)) was detected using laser 600 and 700 on the Odyssey® FC imaging system and protein of interest directed with a yellow arrow. Cleaved BID molecular marker can be seen on the chemiluminescence blot and tracked using the GAPDH fluorescent blot, given Cleaved BID below above GAPDH.

F

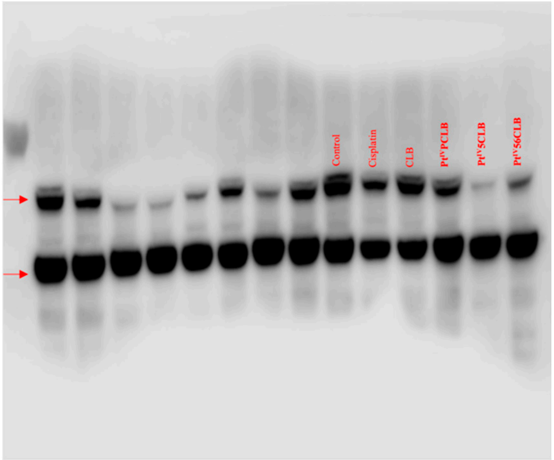

Procaspase 9 –top
GAPDH -bottom

Procaspase 9/GAPDH normalized to control	**Control**	**Cisplatin**	**CLB**	**Pt^{IV}PCLB**	**Pt^{IV}5CLB**	**Pt^{IV}56CLB**
	1	0.524727	0.832875	0.578748	0.095461	0.215279

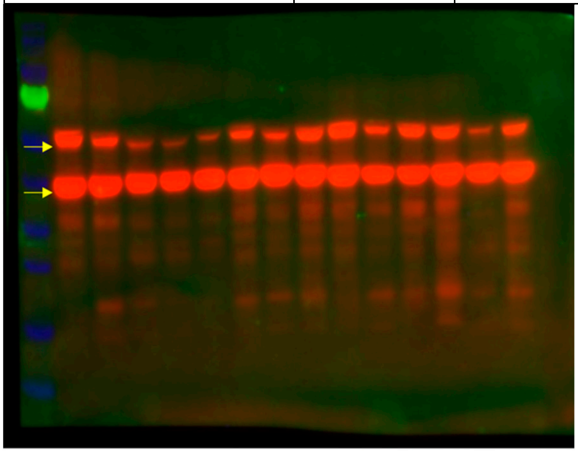

Procaspase 9 -top
GAPDH -bottom

Representative uncropped western blot is presented with protein of interest directed with a red arrow and lanes marked left to right, control, cisplatin, CLB, **Pt^{IV}PCLB**, **Pt^{IV}5CLB** and **Pt^{IV}56CLB** in red, on the chemiluminescence blot. Representative fluorescent western blot with molecular marker (PageRuler Prestained Protein Ladder (Invitrogen #26617)) was detected using laser 600 and 700 on the Odyssey® FC imaging system and protein of interest directed with a yellow arrow.

G

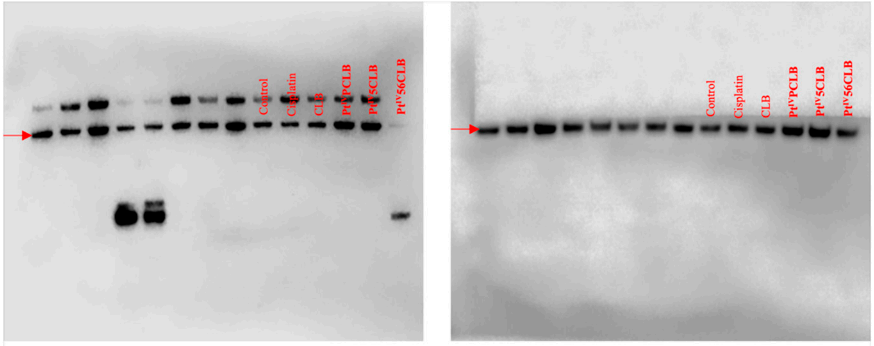

Procaspase 3 **GAPDH**

Procaspase 3/GAPDH normalized to control	Control	Cisplatin	CLB	Pt^{IV}PCLB	Pt^{IV}5CLB	Pt^{IV}56CLB
	1	0.677967	0.761422	0.800269	0.818111	0.097681

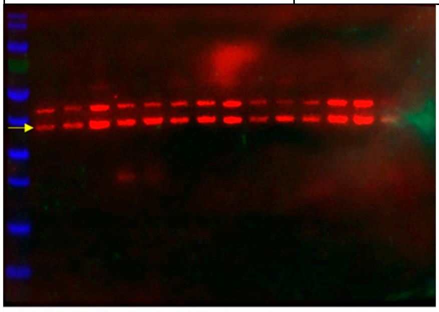

Procaspase 3

Representative uncropped western blot is presented with protein of interest directed with a red arrow and lanes marked left to right, control, cisplatin, CLB, **Pt^{IV}PCLB**, **Pt^{IV}5CLB** and **Pt^{IV}56CLB** in red, on the chemiluminescence blot. Representative fluorescent western blot of GAPDH with molecular marker (PageRuler Prestained Protein Ladder (Invitrogen #26617)) was detected using laser 600 and 700 on the Odyssey® FC imaging system and protein of interest directed with a yellow arrow. GAPDH molecular marker fluorescent blot was not obtained but can be seen on the chemiluminescence blot and tracked using the procaspase 3 fluorescent blot, given GAPDH falls within the same molecular weight.

H

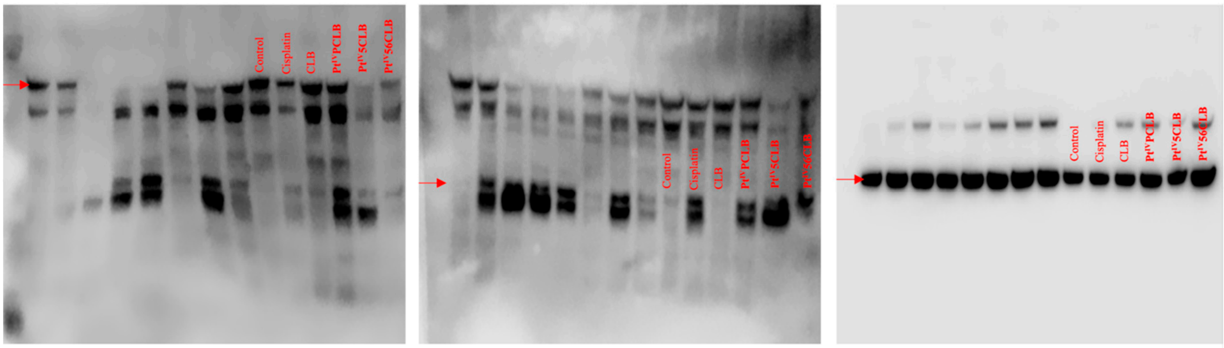

PARP-1 **Cleaved PARP-1** **GAPDH**

	Control	Cisplatin	CLB	Pt^{IV}PCLB	Pt^{IV}5CLB	Pt^{IV}56CLB
PARP-1 /GAPDH normalized to control	1	0.600878	0.880993	0.704975	0.173875	0.344928

Cleaved PARP-1 /GAPDH normalized to control	1	4.660568	0.929223	6.234813	8.362242	6.298707

Representative uncropped western blot is presented with protein of interest directed with a red arrow and lanes marked left to right, control, cisplatin, CLB, **Pt^{IV}PCLB**, **Pt^{IV}5CLB** and **Pt^{IV}56CLB** in red, on the chemiluminescence blot. Representative fluorescent western blot could not be detected with fault in lasers 600 and 700 of Odyssey® FC imaging system requiring replacement. However, manual confirmation was done against the molecular marker (PageRuler Prestained Protein Ladder (Invitrogen #26617)) loaded to the left to confirm the detected band position, given previous knowledge of where the correct band falls for PARP-1 protein detection [5]. This was additionally referenced to antibodies description in Table S6, given previous observational knowledge of GAPDH fluorescent blots.

Figure S17. Full representative western blot of intrinsic and extrinsic apoptotic cell death markers in HT29. Represented data normalized to GAPDH relative to control. A. Bax, B. Bcl2, C. Cytochrome C, D.Procaspase 8, E. Cleaved BID, F. Procaspase 9, G. Procaspase 3 and H. PARP-1. Protein expression upon treatment with CLB ligand **Pt^{IV}PCLB**, **Pt^{IV}5CLB** and **Pt^{IV}56CLB** prodrugs, as well as cisplatin in HT29 cells at 72 h compared with control.

A

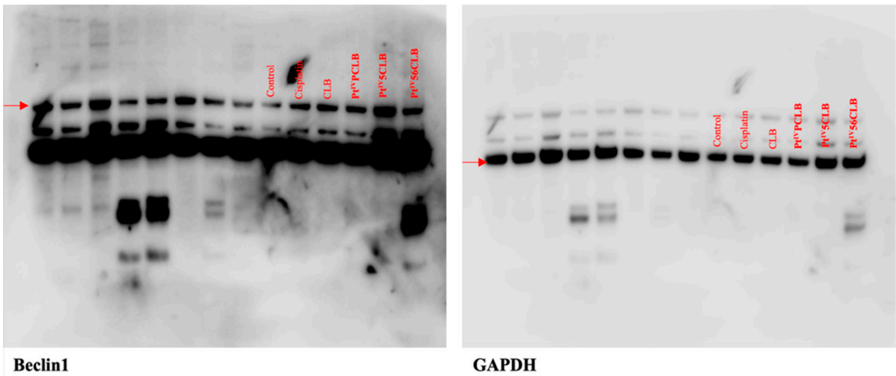

Beclin1/GAPDH normalized to control	Control	Cisplatin	CLB	Pt^{IV}PCLB	Pt^{IV}5CLB	Pt^{IV}56CLB
	1	2.190542	2.791144	2.717229	5.492273	3.705955

Representative uncropped western blot is presented with protein of interest directed with a red arrow and lanes marked left to right, control, cisplatin, CLB, **Pt^{IV}PCLB**, **Pt^{IV}5CLB** and **Pt^{IV}56CLB** in red, on the chemiluminescence blot. Representative fluorescent western blot could not be detected with fault in lasers 600 and 700 of Odyssey® FC imaging system requiring replacement. However, manual confirmation was done against the molecular marker (PageRuler Prestained Protein Ladder (Invitrogen #26617)) loaded to the left to confirm the detected band position, given previous knowledge of where the correct band falls for Beclin1 protein detection [5]. This was additionally referenced to antibodies description in Table S6, given previous observational knowledge of GAPDH fluorescent blots.

B

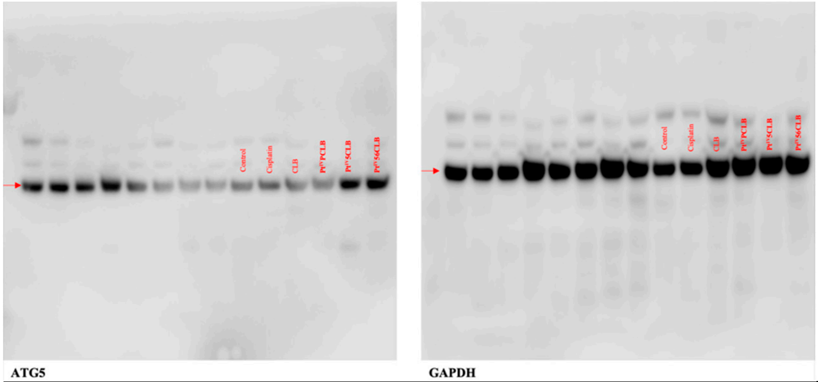

ATG5/GAPDH normalized to control	Control	Cisplatin	CLB	Pt^{IV}PCLB	Pt^{IV}5CLB	Pt^{IV}56CLB
	1	0.919528	1.093785	1.308597	4.404207	4.206563

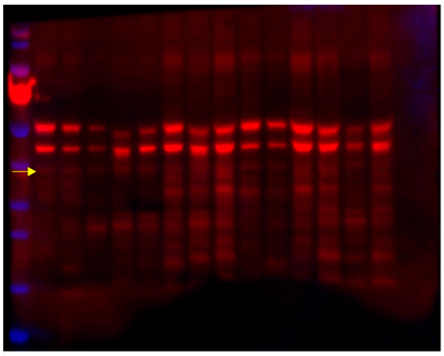

ATG5

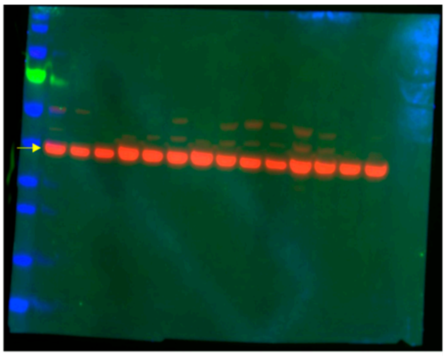

GAPDH

Representative uncropped western blot is presented with protein of interest directed with a red arrow and lanes marked left to right, control, cisplatin, CLB, **Pt^{IV}PCLB**, **Pt^{IV}5CLB** and **Pt^{IV}56CLB** in red, on the chemiluminescence blot. Representative fluorescent western blot with molecular marker (PageRuler Prestained Protein Ladder (Invitrogen #26617)) was detected using laser 600 and 700 on the Odyssey® FC imaging system and protein of interest directed with a yellow arrow.

C

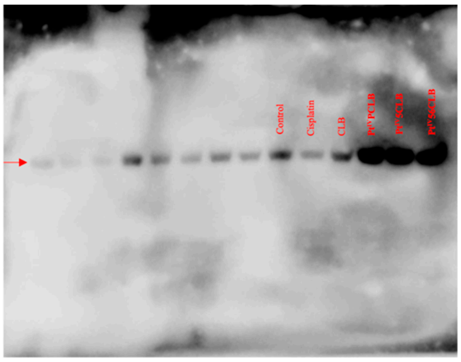

ATG16L1

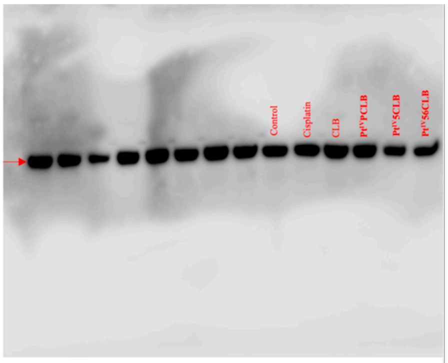

GAPDH

ATG16L1/GAPDH	**Control**	**Cisplatin**	**CLB**	**Pt^{IV}PCLB**	**Pt^{IV}5CLB**	**Pt^{IV}56CLB**
normalized to control	1	0.349283	0.954894	4.993404	5.770186	6.157111

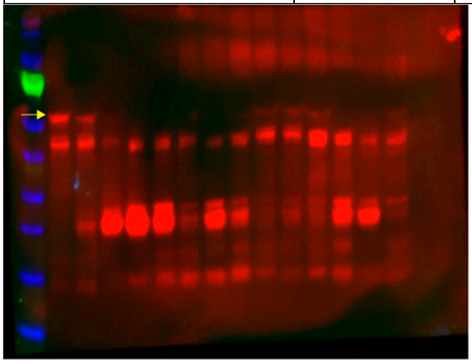

ATG16L1

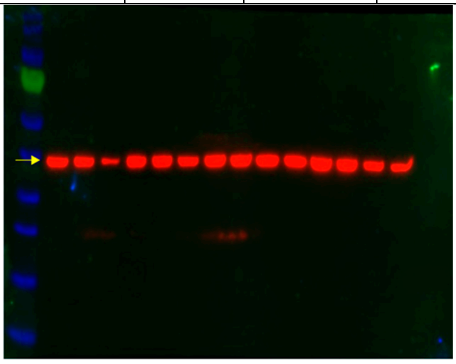

GAPDH

Representative uncropped western blot is presented with protein of interest directed with a red arrow and lanes marked left to right, control, cisplatin, CLB, **Pt^{IV}PCLB**, **Pt^{IV}5CLB** and **Pt^{IV}56CLB** in red, on the chemiluminescence blot. Representative fluorescent western blot with molecular marker (PageRuler Prestained Protein Ladder (Invitrogen #26617)) was detected using laser 600 and 700 on the Odyssey® FC imaging system and protein of interest directed with a yellow arrow. Note: presented ATG16L1 blot was subsequently probed with other markers but presented here to inform the detected band location of ATg16L1 in reference to antibodies description in Table S6

D

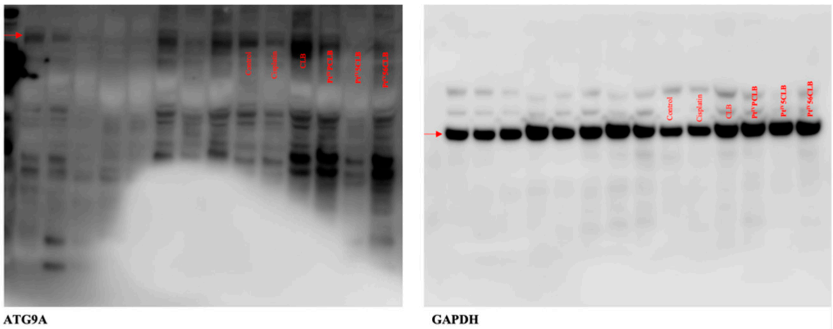

ATG9A/GAPDH normalized to control	**Control**	**Cisplatin**	**CLB**	**Pt^{IV}PCLB**	**Pt^{IV}5CLB**	**Pt^{IV}56CLB**
	1	0.864420	2.901015	0.964259	0.420619	0.760348

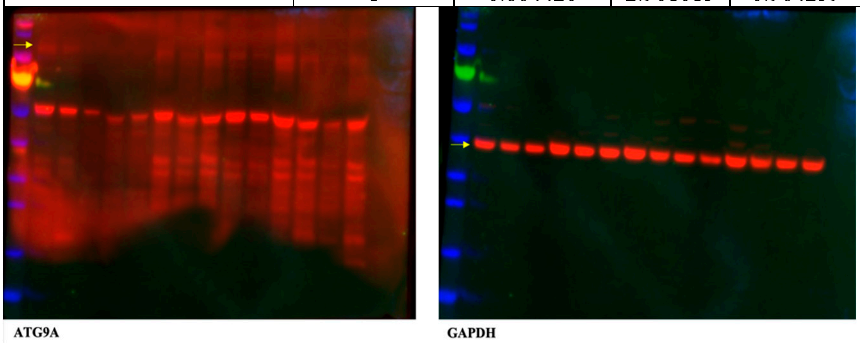

Representative uncropped western blot is presented with protein of interest directed with a red arrow and lanes marked left to right, control, cisplatin, CLB, **Pt^{IV}PCLB**, **Pt^{IV}5CLB** and **Pt^{IV}56CLB** in red, on the chemiluminescence blot. Representative fluorescent western blot with molecular marker (PageRuler Prestained Protein Ladder (Invitrogen #26617)) was detected using laser 600 and 700 on the Odyssey® FC imaging system and protein of interest directed with a yellow arrow.

E

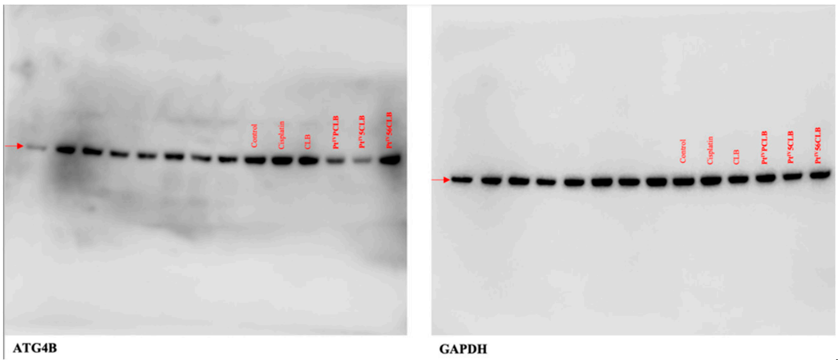

ATG4B/GAPDH normalized to control	**Control**	**Cisplatin**	**CLB**	**Pt^{IV}PCLB**	**Pt^{IV}5CLB**	**Pt^{IV}56CLB**
	1	1.265020	1.317005	0.9856440	0.902425	1.880839

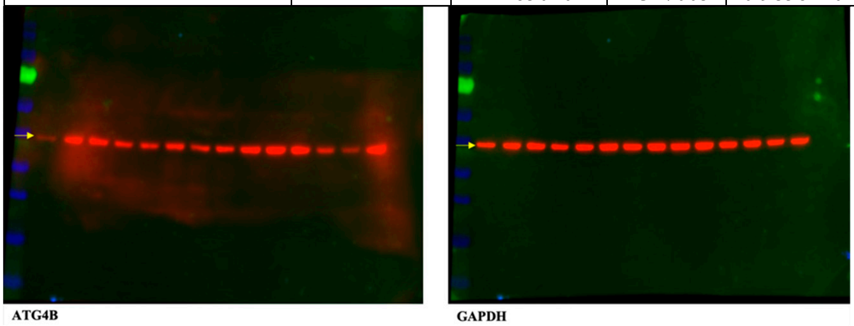

Representative uncropped western blot is presented with protein of interest directed with a red arrow and lanes marked left to right, control, cisplatin, CLB, **Pt^{IV}PCLB**, **Pt^{IV}5CLB** and **Pt^{IV}56CLB** in red, on the chemiluminescence blot. Representative fluorescent western blot with molecular marker (PageRuler Prestained

Protein Ladder (Invitrogen #26617)) was detected using laser 600 and 700 on the Odyssey® FC imaging system and protein of interest directed with a yellow arrow.

F

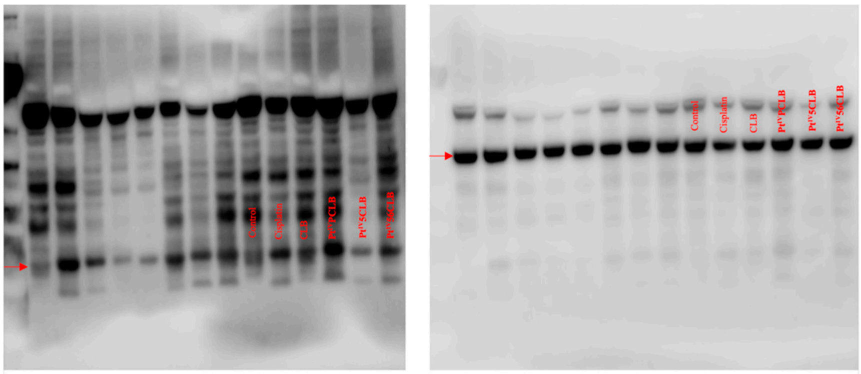

LC3B **GAPDH**

LC3B/GAPDH normalized to control	**Control**	**Cisplatin**	**CLB**	**Pt^{IV}PCLB**	**Pt^{IV}5CLB**	**Pt^{IV}56CLB**
	1	3.101690	1.787327	2.780206	1.893634	2.506115

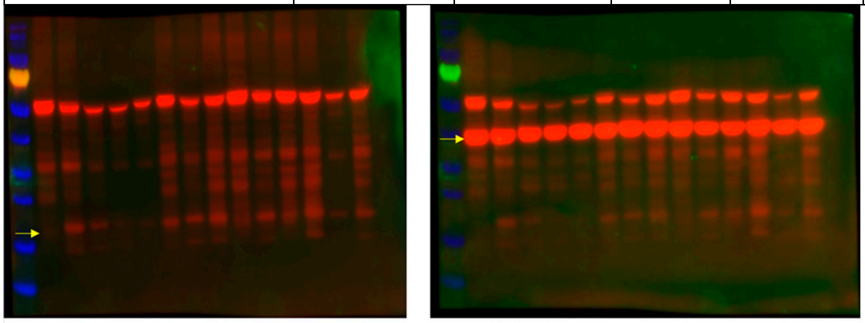

LC3B **GAPDH**

Representative uncropped western blot is presented with protein of interest directed with a red arrow and lanes marked left to right, control, cisplatin, CLB, **Pt^{IV}PCLB**, **Pt^{IV}5CLB** and **Pt^{IV}56CLB** in red, on the chemiluminescence blot. Representative fluorescent western blot with molecular marker (PageRuler Prestained Protein Ladder (Invitrogen #26617)) was detected using laser 600 and 700 on the Odyssey® FC imaging system and protein of interest directed with a yellow arrow.

Figure S18. Full representative western blot of autophagy markers in HT29. Represented data normalized to GAPDH relative to control. A. Beclin1, B. APG5L/ATG5, C. ATG16L1, D. ATG9A, E. ATG4B and F. LC3B. Protein expression upon treatment with CLB ligand, **Pt^{IV}PCLB**, **Pt^{IV}5CLB** and **Pt^{IV}56CLB** prodrugs, as well as cisplatin in HT29 cells at 72 h compared with control.

References

1. Pathak, R.K.; Wen, R.; Kolishetti, N.; Dhar, S. A Prodrug of Two Approved Drugs, Cisplatin and Chlorambucil, for Chemo War Against Cancer. *Mol Cancer Ther* **2017**, *16*, 625-636, doi:10.1158/1535-7163.Mct-16-0445.
2. Aputen, A.D.; Elias, M.G.; Gilbert, J.; Sakoff, J.A.; Gordon, C.P.; Scott, K.F.; Aldrich-Wright, J.R. Potent Chlorambucil-Platinum(IV) Prodrugs. *Int J Mol Sci* **2022**, *23*, doi:10.3390/ijms231810471.
3. Deo, K.M.; Sakoff, J.; Gilbert, J.; Zhang, Y.; Aldrich Wright, J.R. Synthesis, characterisation and potent cytotoxicity of unconventional platinum(iv) complexes with modified lipophilicity. *Dalton Trans* **2019**, *48*, 17217-17227, doi:10.1039/c9dt03339d.
4. Macias, F.J.; Deo, K.M.; Pages, B.J.; Wormell, P.; Clegg, J.K.; Zhang, Y.; Li, F.; Zheng, G.; Sakoff, J.; Gilbert, J.; et al. Synthesis and Analysis of the Structure, Diffusion and Cytotoxicity of Heterocyclic Platinum(IV) Complexes. *Chemistry* **2015**, *21*, 16990-17001, doi:10.1002/chem.201502159.
5. Elias, M.G., Fatima, S., Mann, T., Karan, S., Mikhael, M., Souza, P., Gordon, C.P, Scott, K. F., Aldrich-Wright, J.R. Anticancer Effect of PtIIPHENSS, PtII5MESS, PtII56MESS and their Platinum(IV)-dihydroxy derivatives Against Triple-Negative Breast Cancer and Cisplatin-Resistant Colorectal Cancer *Cancers* **2024**, *16*, doi:https://doi.org/10.3390/cancers16142544.
